# Genomes of leafy and leafless *Platanthera* orchids illuminate the evolution of mycoheterotrophy

**DOI:** 10.1038/s41477-022-01127-9

**Published:** 2022-04-21

**Authors:** Ming-He Li, Ke-Wei Liu, Zhen Li, Hsiang-Chia Lu, Qin-Liang Ye, Diyang Zhang, Jie-Yu Wang, Yu-Feng Li, Zhi-Ming Zhong, Xuedie Liu, Xia Yu, Ding-Kun Liu, Xiong-De Tu, Bin Liu, Yang Hao, Xing-Yu Liao, Yu-Ting Jiang, Wei-Hong Sun, Jinliao Chen, Yan-Qiong Chen, Ye Ai, Jun-Wen Zhai, Sha-Sha Wu, Zhuang Zhou, Yu-Yun Hsiao, Wan-Lin Wu, You-Yi Chen, Yu-Fu Lin, Jui-Ling Hsu, Chia-Ying Li, Zhi-Wen Wang, Xiang Zhao, Wen-Ying Zhong, Xiao-Kai Ma, Liang Ma, Jie Huang, Gui-Zhen Chen, Ming-Zhong Huang, Laiqiang Huang, Dong-Hui Peng, Yi-Bo Luo, Shuang-Quan Zou, Shi-Pin Chen, Siren Lan, Wen-Chieh Tsai, Yves Van de Peer, Zhong-Jian Liu

**Affiliations:** 1 Key Laboratory of Orchid Conservation and Utilization of National Forestry and Grassland Administration at College of Landscape Architecture, Fujian Agriculture and Forestry University, Fuzhou, China; 2 Fujian Colleges and Universities Engineering Research Institute of Conservation and Utilization of Natural Bioresources, College of Forestry, Fujian Agriculture and Forestry University, Fuzhou, China; 3 Tsinghua-Berkeley Shenzhen Institute (TBSI), Center for Biotechnology and Biomedicine, Shenzhen Key Laboratory of Gene and Antibody Therapy, State Key Laboratory of Chemical Oncogenomics, State Key Laboratory of Health Sciences and Technology, Institute of Biopharmaceutical and Health Engineering (iBHE), Shenzhen International Graduate School, Tsinghua University, Shenzhen, China; 4 Department of Plant Biotechnology and Bioinformatics, Ghent University, Ghent, Belgium; 5 VIB Center for Plant Systems Biology, Ghent, Belgium; 6 Institute of Tropical Plant Sciences, National Cheng Kung University, Tainan, Taiwan; 7 Zijin Baixi Provincial Nature Reserve of Guangdong, Heyuan, China; 8 Key Laboratory of Plant Resources Conservation and Sustainable Utilization, South China Botanical Garden, Chinese Academy of Sciences, Guangzhou, China; 9 Orchid Research and Development Center, National Cheng Kung University, Tainan, Taiwan; 10 Department of Life Sciences, National Cheng Kung University, Tainan, Taiwan; 11 Department of Applied Chemistry, National Pingtung University, Pingtung, Taiwan; 12 PubBio-Tech, Wuhan, China; 13 State Key Laboratory of Systematic and Evolutionary Botany, Institute of Botany, Chinese Academy of Sciences, Beijing, China; 14 Center for Microbial Ecology and Genomics, Department of Biochemistry, Genetics and Microbiology, University of Pretoria, Pretoria, South Africa; 15 College of Horticulture, Academy for Advanced Interdisciplinary Studies, Nanjing Agricultural University, Nanjing, China; 16 Henry Fok College of Biology and Agriculture, Shaoguan University, Shaoguan, China

**Keywords:** Plant evolution, Genome evolution

## Abstract

To improve our understanding of the origin and evolution of mycoheterotrophic plants, we here present the chromosome-scale genome assemblies of two sibling orchid species: partially mycoheterotrophic *Platanthera zijinensis* and holomycoheterotrophic *Platanthera guangdongensis*. Comparative analysis shows that mycoheterotrophy is associated with increased substitution rates and gene loss, and the deletion of most photoreceptor genes and auxin transporter genes might be linked to the unique phenotypes of fully mycoheterotrophic orchids. Conversely, trehalase genes that catalyse the conversion of trehalose into glucose have expanded in most sequenced orchids, in line with the fact that the germination of orchid non-endosperm seeds needs carbohydrates from fungi during the protocorm stage. We further show that the mature plant of *P. guangdongensis*, different from photosynthetic orchids, keeps expressing trehalase genes to hijack trehalose from fungi. Therefore, we propose that mycoheterotrophy in mature orchids is a continuation of the protocorm stage by sustaining the expression of trehalase genes. Our results shed light on the molecular mechanism underlying initial, partial and full mycoheterotrophy.

## Abstract (web summary)

Analyses of the genome sequences and expression data for two closely related mycoheterotrophic orchid species provide insights into the genomic basis underlying the evolution of mycoheterotrophy.

## Main

Most plants obtain energy via carbohydrates through photosynthesis, but some plant lineages can make use of carbohydrates through other organisms. These two different life strategies are often referred to as “autotrophy” and “heterotrophy,” respectively. “Mycoheterotrophy” refers to a plant’s ability to obtain carbohydrates from fungi rather than from photosynthesis^1^. Mycoheterotrophic plants can be one of three types: “fully mycoheterotrophic” plants solely depending on ‘fungal carbon’ during their entire life cycle; “initially mycoheterotrophic” plants utilizing fungal carbon during the early stages of their development; and “partially mycoheterotrophic” or “mixotrophic” plants combining mycoheterotrophy and autotrophy to obtain carbon during at least one stage of their life cycle^2^. More than 30,000 plant species are mycoheterotrophic, among which are 880 full mycoheterotrophs. Mycoheterotrophic plants exist in most if not all major land plant lineages, including liverworts, ferns, lycophytes, gymnosperms and angiosperms^2^. In angiosperms, seven monocot families, namely Orchidaceae, Petrosaviaceae, Triuridaceae, Burmanniaceae, Thismiaceae, Corsiaceae and Iridaceae, and three eudicot families, namely Polygalaceae, Ericaceae and Gentianaceae, include mycoheterotrophic species^1,2^. Although most of the fully mycoheterotrophic flowering plants are restricted to tropical regions, some of them (from Ericaceae and Orchidaceae) are also found in temperate forests^3^.

Mycoheterotrophic plants have long attracted the interest of botanists and mycologists and have been the subject of unabated controversy and speculation for over two centuries^4^. Previous studies on mycoheterotrophy have focused on physiological ecology^5–7^, the associated mycorrhizal fungi^8^ and chloroplast genome evolution^9–13^. However, it is not clear how mycoheterotrophy has evolved within various autotrophic lineages. To solve this question, Orchidaceae may be a critical family that could shed light on the evolution of mycoheterotrophy, because at least 30 out of more than 40 documented independent transitions to full mycoheterotrophy in land plants have occurred in Orchidaceae^14^. Moreover, partial mycoheterotrophy is also common in Orchidaceae, where species keep obtaining carbohydrates from fungi even after they can perform photosynthesis. Examples are *Apostasia* of the subfamily Apostasioideae^15^, *Cephalanthera*, *Epipactis* and *Cymbidium* of the subfamily Epidendroideae^5,16,17^ and *Platanthera* of the subfamily Orchidoideae^18^. Actually, all species in Orchidaceae, one of the largest and most diverse plant families with more than 25,000 species^19^, are initial mycoheterotrophs during their protocorm (germination) stage. All orchids have small, dust-like seeds with no endosperm, and hence limited nutrition for germination^2^ and need fungi as a source for carbon. Consequently, partial or full mycoheterotrophy in mature orchids may be a continued or derived form of their protocorm stage.

**Fig. 1:**
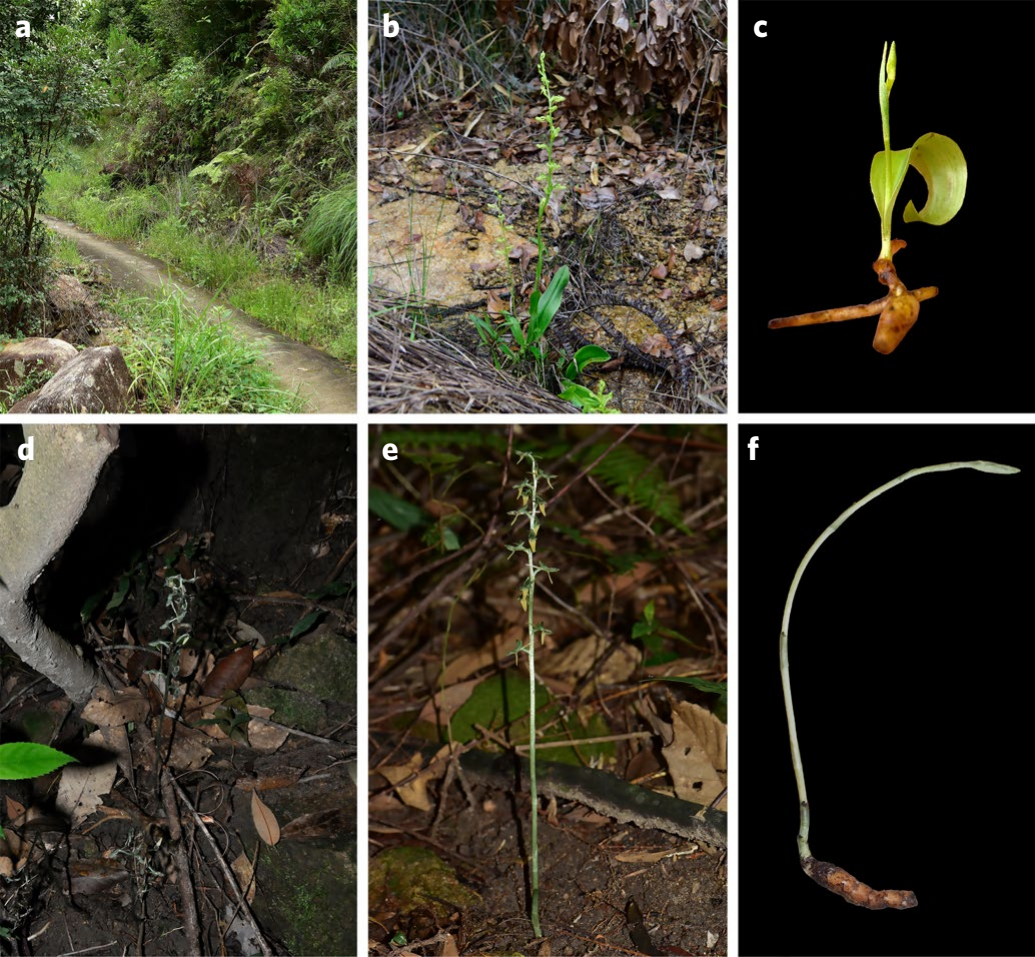
The natural habitat and appearance of *P. zijinensis* and *P. guangdongensis*. **a**, The natural habitat of *P. zijinensis*. **b**, Flowering plant of *P. zijinensis* growing on the open terrain. **c**, *P. zijinensis* plant with tubercles, root and leaf. **d**, The natural habitat of *P. guangdongensis*. **e**, Flowering plant of *P. guangdongensis* growing in a shady forest understory. **f**, *P. guangdongensis* plant without root and leaf but with an underground stem (tuber).

Here we present chromosome-scale assembled genomes of two closely related orchids^20^: a partially mycoheterotrophic orchid, *Platanthera zijinensis*, and a fully mycoheterotrophic orchid, *Platanthera guangdongensis*^2,18^. The two species are only found in one location in Zijin County, Guangdong Province, China, where they live alongside each other but in quite different habitats, with *P. zijinensis* mainly growing on light-abundant rocky hills and *P. guangdongensis* in the adjacent forest with a dense canopy (Fig. 1). Also, *P. zijinensis* has leaves and a root system with tubercles, whereas *P. guangdongensis* has neither leaves nor roots, but has a tuber (vertical, underground stem), the organ to which the mycorrhizal fungi, critical to its holomycoheterotrophic lifestyle, are attached (Fig. 1). Comparing the high-quality genomes of *P. zijinensis* and *P. guangdongensis*, together with other sequenced orchids, would hence enable us to study the evolution of initial, partial and full mycoheterotrophy in orchids. The two sequenced genomes of *P. zijinensis* and *P. guangdongensis* would also fill the gap between sequenced orchid genomes of photosynthetic orchids^21–23^ on one side and non-photosynthetic orchids on the other side^24^, paving a way to identify possible genes related to mycoheterotrophy in Orchidaceae. Further, we investigate genes related to mycoheterotrophy at different developmental stages of both photosynthetic and non-photosynthetic orchids, providing insights into the origin and evolution of mycoheterotrophy in Orchidaceae.

## Results and discussion

### Genome sequencing and genome characteristics

*P. zijinensis* and *P. guangdongensis* both have a karyotype of 2*n* = 2X = 42 chromosomes. To completely sequence the genomes, we generated a total of 441.16 Gb and 414.20 Gb data for *P. zijinensis* and *P. guangdongensis*, respectively, with multiple insert libraries using PacBio technologies (Supplementary Tables 1 and 2). With *k*-mer analyses we estimated a genome size of 4.15 Gb for the *P. zijinensis* genome with a heterozygosity of 1.80% and a genome size of 4.27 Gb for the *P. guangdongensis* genome with a heterozygosity of 1.89% (Supplementary Figs. 1, 2), thus indicating relatively high heterozygosity levels for both genomes. The total length of the genome assembly was 4.19 Gb with a contig N50 value of 1.77 Mb for the *P. zijinensis* genome and 4.20 Gb with a contig N50 value of 1.57 Mb for the *P. guangdongensis* genome (Supplementary Table 3). Benchmarking Universal Single-Copy Orthologs (BUSCO) analysis^25^ indicated that genome assembly completeness is 88.66% and 72.06% for *P. zijinensis* and *P. guangdongensis*, respectively (Supplementary Table 4). We further used Illumina sequencing reads from HiC libraries to reconstruct physical maps by ordering and clustering the assembled scaffolds into 21 pseudomolecules in each species, to represent the 21 chromosomes in each haploid genome of *P. zijinensis* and *P. guangdongensis* (Supplementary Fig. 3). The pseudochromosome sizes of *P. zijinensis* ranged from 144.78 Mb to 288.56 Mb with N50 of 192.35 Mb, and the pseudochromosome sizes of *P. guangdongensis* ranged from 143.09 Mb to 306.18 Mb with N50 of 193.14 Mb (Supplementary Tables 5, 6). The chromatin interaction data suggest a high quality of the HiC assemblies.

The genomes of *P. zijinensis* and *P. guangdongensis* have, so far, been the largest assembled genomes among all the sequenced orchid species. Their large genome sizes are due to the large numbers of repetitive elements in the genomes. Using a combination of structural information and homology prediction, we identified a total of 3.24 Gb and 3.45 Gb of repetitive elements occupying about 77.38% and 82.18% of the *P. zijinensis* and *P. guangdongensis* genomes, respectively. Long terminal repeats (LTRs), specifically *LTR/Gypsy* and *LTR/Copia*, are the most abundant retrotransposons, accounting for over half of the genomes of *P. zijinensis* (71.78%) and *P. guangdongensis* (73.24%) (Supplementary Table 7). Interestingly, not only do *P. zijinensis* and *P. guangdongensis* have the largest genomes, but their genomes also have the highest LTR contents compared with other sequenced orchid genomes such as *Phalaenopsis equestris* (44.19%), *Dendrobium catenatum* (39.88%), *Apostasia shenzhenica* (16.81%) and *Gastrodia elata* (54.75%) (Supplementary Table 8). Analysis of the ‘insertion time’ of LTR, *Copia* and *Gypsy* elements of *P. zijinensis* showed that LTR insertion has been a continuous process, although 79.45% of the insertions occurred before 0.2 million years ago (Ma) (Methods, Supplementary Fig. 4a and Supplementary Table 9). For *P. guangdongensis*, the insertion of total LTR, *Copia* and *Gypsy* elements was also shown to have been a continuous process that accounted for 60.95% of the total insertions occurring before 0.8 Ma (Methods, Supplementary Fig. 4b and Supplementary Table 10).

We confidently annotated 24,513 and 22,559 protein-coding genes in the genomes of *P. zijinensis* and *P. guangdongensis*, respectively (Supplementary Table 11), of which 87.17% of the *P. zijinensis* and 86.07% of the *P. guangdongensis* genes had functional annotations (Supplementary Table 12). To compare the *P. guangdongensis* and *P. zijinensis* genomes with another, previously published, fully mycoheterotrophic orchid genome, that is, the *G. elata* genome^24^, we re-annotated 18,019 protein-coding genes in the *G. elata* genome (Supplementary Table 11). In addition, we identified 31 and 33 micro-ribonucleic acids (microRNAs), 994 and 1,015 transfer RNAs, 4,187 and 2,533 ribosomal RNAs and 615 and 152 small nuclear RNAs in the *P. zijinensis* and *P. guangdongensis* genomes, respectively (Supplementary Table 13).

The gene numbers of *P. zijinensis* and *P. guangdongensis* were smaller than those of the previously sequenced photosynthetic orchids *Pha. equestris* (26,471) and *D. catenatum* (26,791) but not *A. shenzhenica* (20,560)^23^, and larger than that of the fully mycoheterotrophic *G. elata*. BUSCO assessment indicated that, compared with the complete BUSCO genes in *A. shenzhenica* (82.96%), *Pha. equestris* (76.82%) and *D. catenatum* (76.95%), the partial mycoheterotrophic *P. zijinensis* has a comparable set of 1,288 (79.80%) complete BUSCOs but the fully mycoheterotrophic *P. guangdongensis* has only 949 (58.80%) complete BUSCO genes, comparable with the 1,061 (65.74%) complete BUSCO genes in the *G. elata* genome (Supplementary Table 14), and suggestive of full mycoheterotrophs having lost a significant fraction of genes. Indeed, among the 488 (30.24%) BUSCO genes lost in *P. guangdongensis* and the 450 (27.88%) BUSCO genes lost in *G. elata*, 273 BUSCO genes (55.94% of 488 in *P. guangdongensis* and 60.67% of 450 in *G. elata*) are lost in common, suggesting that both sequenced, fully mycoheterotrophic orchids lost a significant fraction of (the same) genes (Supplementary Fig. 5).

### Genome evolution of *P. zijinensis* and *P. guangdongensis*

**Fig. 2:**
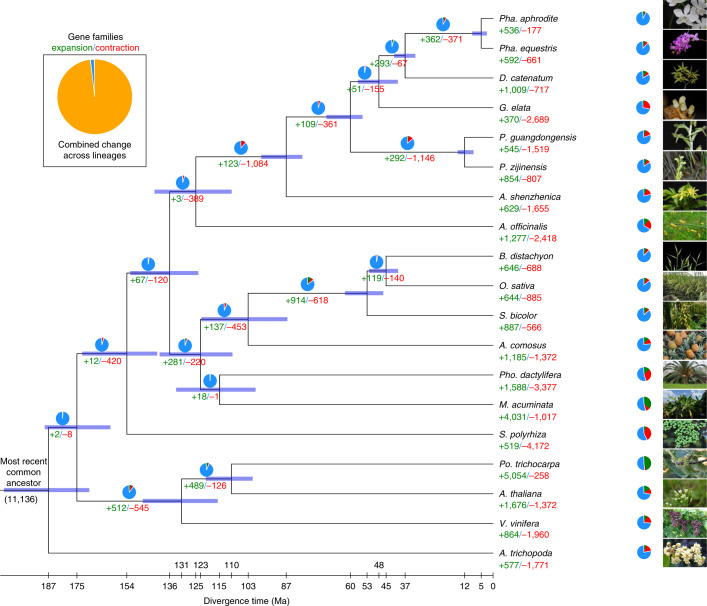
Phylogenetic tree showing divergence times and the evolution of gene-family sizes. Phylogenetic tree showing the topology and divergence times for 19 plant species. Divergence times are indicated by light blue bars at the internodes. The range of the light blue bars indicates the 95% highest posterior density of the divergence time. Numbers in green and red at branches indicate the expansion and contraction of gene families, respectively. The pie chart colours represent the changes of gene family (blue, no significant change; orange, expanded or contracted; green, expanded; red, contracted); each colour sector illustrates the proportion of each type of gene-family change. We set different birth and death rates for different branches (Poaceae, Orchidaceae, other monocots and the other branches) and used the likelihood ratio test to select the optimized sets of rates (Methods).

We constructed a high-confidence phylogenetic tree and estimated the divergence times of 19 different plant species based on genes extracted from a total of 234 single-copy families (Methods). As expected, *P. zijinensis* and *P. guangdongensis* are two species in the subfamily Orchidoideae, forming a sister group to the subfamily Epidendroideae in which *G. elata* successively clustered with *D. catenatum* and both sequenced *Phalaenopsis* genomes. The divergence time between the subfamily Orchidoideae (*Platanthera*) and the subfamily Epidendroideae (*G. elata*, *Pha. equestris*, *Phalaenopsis aphrodite* and *D. catenatum*) has been estimated to be approximately 60.38 Ma with a 95% highest posterior density of 54.29–69.31 Ma, while the divergence time between *P. zijinensis* and *P. guangdongensis* has been estimated at approximately 11.63 Ma with a 95% highest posterior density of 8.15–14.71 Ma (Fig. 2).

**Fig. 3:**
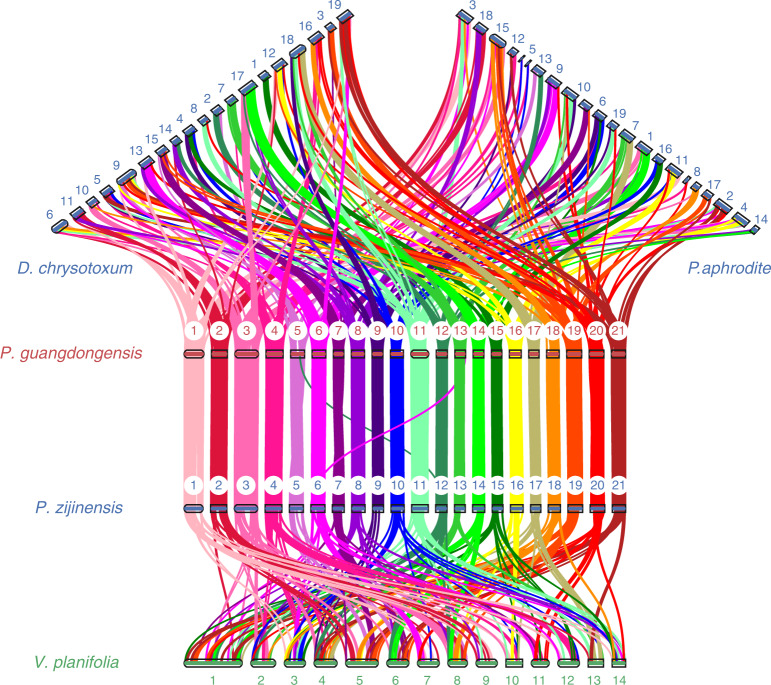
Chromosome structure and collinearity of *Pha. aphrodite*, *D. chrysotoxum*, *V. planifolia*, *P. zijinensis* and *P. guangdongensis*. Chromosome comparison for *P. zijinensis* and *P. guangdongensis* shows almost perfect collinearity except for two rearrangements within Chr 5 and Chr 7, and two translocations (see also Supplementary Fig. 6). In comparison with *P. zijinensis* and *P. guangdongensis*, the differences in chromosome number of three other orchid genomes exhibited major chromosome rearrangement events.

Comparative analysis shows that the genomes of *P. zijinensis* and *P. guangdongensis* are almost perfectly collinear (Supplementary Fig. 6 and Supplementary Table 15), except for two rearrangements within chromosome (Chr) 5 and Chr 7, and two translocations between Chr 6 of *P. zijinensis* and Chr 13 of *P. guangdongensis* and Chr 12 of *P. zijinensis* and Chr 5 of *P. guangdongensis* (Fig. 3 and Supplementary Fig. 6). Other chromosome-level assembled orchid genomes have different chromosome numbers, for example, 14 chromosomes for *Vanilla planifolia*^26^, 19 for *Pha. aphrodite*^27^ and 19 for *Dendrobium chrysotoxum*^28^. However, the differences in chromosome numbers are not due to a few chromosome fusions and fissions, but, as our collinear results indicate, major chromosome rearrangement events must have occurred in different lineages after the whole-genome duplication before the divergence of Orchidaceae^23^ (Supplementary Figs. 7 and 8).

### Mycoheterotrophy is associated with increased substitution rates

**Fig. 4:**
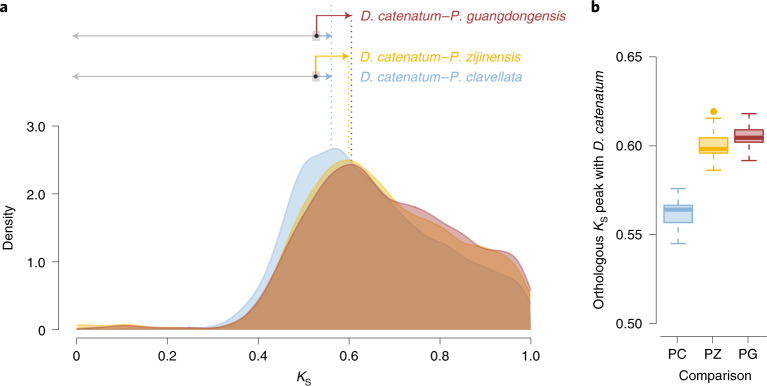
Increased synonymous substitution rates in *P. zijinensis* and *P. guangdongensis*. **a**, Kernel-density estimates (KDEs) of *K*_S_ distributions for one-to-one orthologues between *D. catenatum* and three *Platanthera* orchids, namely *P. guangdongensis*, *P. zijinensis* and *P. clavellata*. As the modes (peaks) of the KDE all represent the distances between *D. catenatum* and the compared orchids, the differences observed among the *K*_S_ values of the modes indicate substitution rate variations among these Orchidaceae lineages. The segments with arrows above the KDE denote, from bottom to top, the *K*_S_ distances calculated for *P. zijinensis* (yellow) and *P. guangdongensis* (red) using *P. clavellata* (blue) as the reference and *D. catenatum* as the outgroup (Methods). The dotted lines align with the observed modes in the KDE of the ortholog *K*_S_ distributions. The black dots on the segments represent the divergence between *P. clavellata* with the other two *Platanthera* orchids; hence, the lengths of segments pointed to the right show the *K*_S_ accumulated in *P. zijinensis* and *P. guangdongensis*, whereas the grey segments pointed to the left show the *K*_S_ distance between *D. catenatum* and the divergence of the three *Platanthera* orchids. The grey rectangles show the 95% confidence intervals of the *K*_S_ inferred from 200 bootstraps. **b**, Boxplots showing modes of the one-to-one orthologous *K*_S_ distributions between *D. catenatum* and *P. clavellata* (PC), *D. catenatum* and *P. zijinensis* (PZ) and *D. catenatum* and *P. guangdongensis* (PG) by resampling the corresponding *K*_S_ distributions 200 times. The line in the middle of a box represents the median value and the top and bottom borders of the boxes denote the 75th and 25th percentiles, respectively. The upper and lower bars show the largest value within 1.5 times the interquartile range above the 75th percentile and the smallest value within 1.5 times the interquartile range below the 25th percentile, respectively. A dot shows the outside value, which is >1.5 times and <3 times the interquartile range beyond either end of the box.

**Extended Data Fig. 1:**
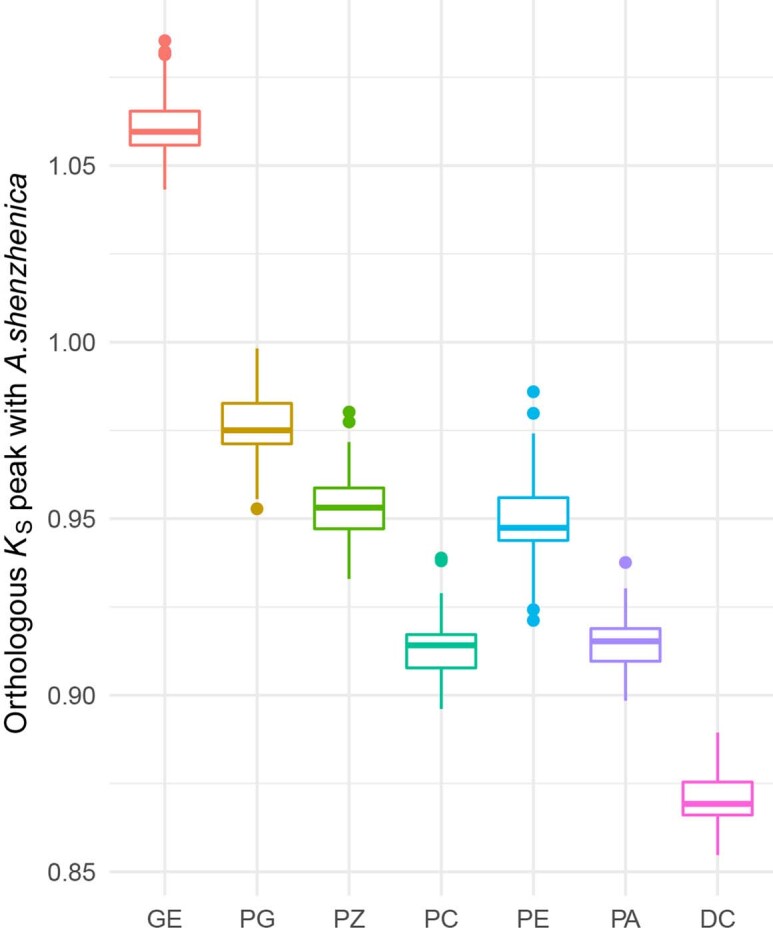
The orthologous *Ks* distributions. Modes of the one-to-one orthologous *K*s distributions between *A. shenzhenica* and each of *G. elata* (GE), *P. guangdongensis* (PG), *P. zijinensis* (PZ), *P. clavellate* (PC), *Pha. equestris* (PE), *Pha. aphrodite* (PA), and *D. catenatum* (DC) by resampling the corresponding *K*s distributions 200 times. The line in the middle of a box represents the median value and the up and bottom borders of the boxes denote the 75th and 25th percentiles, respectively. The upper and lower bars show the largest value within 1.5 times interquartile range above 75th percentile and the smallest value within 1.5 times interquartile range below 25th percentile, respectively. A dot shows outside value, which is > 1.5 times and < 3 times the interquartile range beyond either end of the box.

The phylogram of the 19 species mentioned earlier also shows that the branches leading to *P. zijinensis* and *P. guangdongensis*, after the divergence between Orchidoideae and Epidendroideae, are longer than the branches leading to *Pha. equestris*, *Pha. aphrodite* and *D. catenatum*, suggesting a potentially increased substitution rate of *P. zijinensis* and *P. guangdongensis* (Supplementary Fig. 9). To quantify the differences in substitution rates, we compared the number of synonymous substitutions per synonymous site (*K*_S_) of one-to-one orthologues between *A. shenzhenica* and *G. elata*, *Pha. equestris*, *D. catenatum*, *P. zijinensis*, *P. guangdongensis* and an autotrophic species, *Platanthera clavellata*^29^. Because the peaks (modes) of these orthologous *K*_S_ distributions all represent the same speciation event (*A*. *shenzhenica* and other sequenced orchids), the *K*_S_ values of orthologous peaks should be identical in case of identical substitution rates. Nevertheless, different orthologous *K*_S_ peaks indicate distinctly synonymous substitution rates in these orchid species, with the highest found in *G. elata* and the lowest in *D. catenatum*, suggesting that *G. elata*, as a fully mycoheterotrophic orchid, may have an accelerated substitution rate (Extended Data Fig. 1). This is supported by our observations for *Platanthera*. When comparing the orthologous *K*_S_ peaks between *D. catenatum* (or *A*. *shenzhenica* in Extended Data Fig. 1) and the three *Platanthera* species, that is, the autotrophic *P*. *clavellata*, the partially mycoheterotrophic *P. zijinensis* and the fully mycoheterotrophic *P. guangdongensis* (Fig. 4), we observe an increasing trend of *K*_S_ distances along with the change in trophic styles from autotrophy to mycoheterotrophy, suggesting increased substitution rates in the partially and fully mycoheterotrophic species. Similar patterns of increased substitution rates have been observed in other heterotrophic plants, for instance in obligate parasitic plants such as *Cuscuta australis*^30^ and *Cassytha filiformis*^31^.

### Mycoheterotrophy is associated with gene loss

**Fig. 5:**
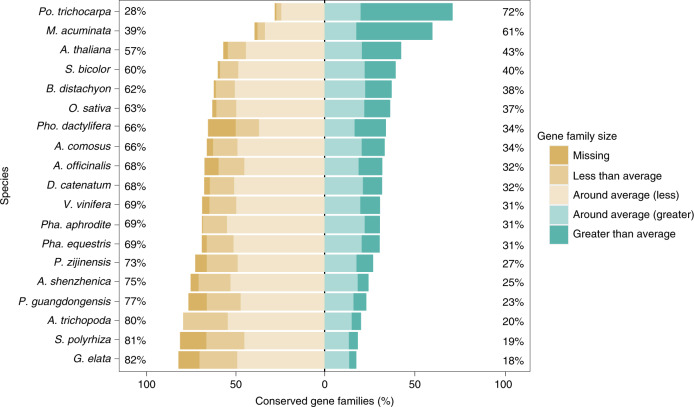
Gene loss in the genomes of *P. guangdongensis* and *G. elata*. For all the investigated species, we compared the size of each gene family with the average size of a gene family through calculating the *F* index (Methods). The *F* index for gene family ranges from 0 to 1, with 0.5 representing the size of a gene family in a species to be exactly equal to its average size. We hence classified 8,423 gene families that exist in 16 out of 19 angiosperm species and have homologues from *A. trichopoda* into five categories: “Missing” with *F* index equal to 0; “Less than average” with *F* index greater than 0 but less than or equal to 0.45; “Around average (less)” with *F* index greater than 0.45 but less than or equal to 0.5; “Around average (greater)” with *F* index greater than 0.5 but less than or equal to 0.55; and “Greater than average” with *F* index greater than 0.55. The bar plot illustrates the percentage of the conserved gene families in each category and the values on the left and right sides show the percentages of the gene families that are smaller and larger than the average size, respectively. See Extended Data Fig. 2 for the number of missing gene families in each species.

**Extended Data Fig. 2:**
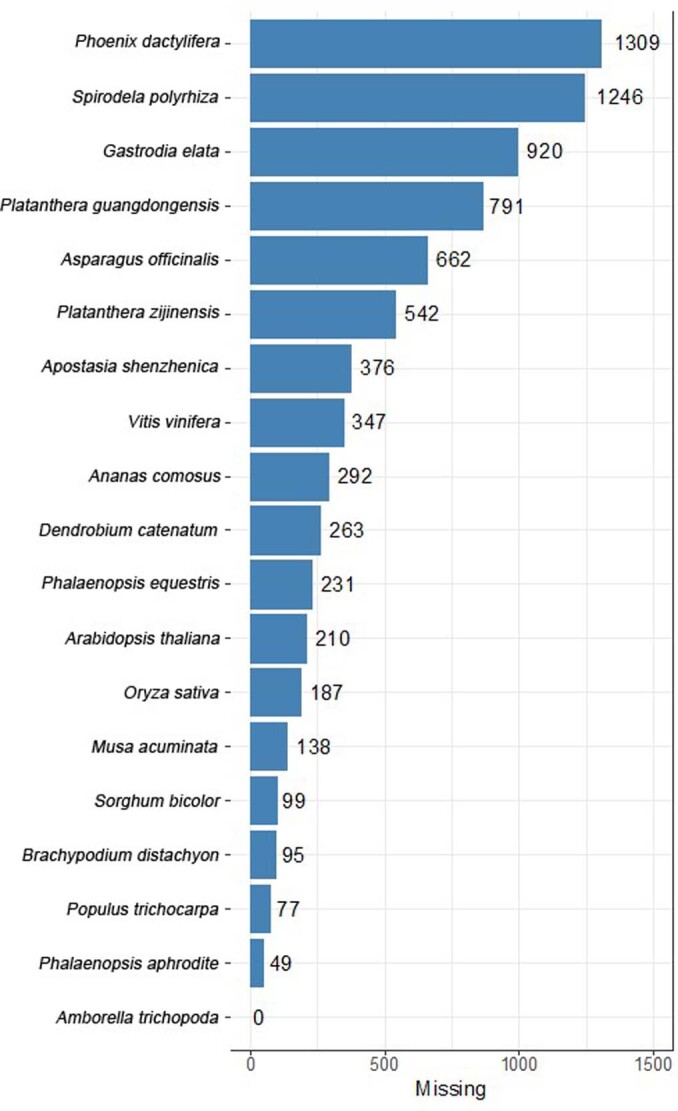
The missing gene families. Number of missing gene families in sequenced plant genomes.

Extensive gene loss has been observed in the genomes of parasitic^30^ and fully mycoheterotrophic^24^ plants. To quantify and compare the level of gene loss in partial and full mycoheterotrophs, we conducted homologous gene identification and gene family cluster analysis and obtained 35,560 clustered gene families for 19 sequenced plant species and 12,539 and 12,014 gene families for *P. zijinensis* and *P. guangdongensis*, respectively (Supplementary Table 16). We then selected 8,423 gene families that exist in at least 16 out of the 19 above-mentioned species and have homologues in *Amborella trichopoda*. For these gene families that are probably conserved across angiosperms, we compared the observed gene-family size in each species and the average gene-family size of each gene family (Methods and Fig. 5). Most of these gene families in each species have several genes close to the average size, but 791 gene families in *P. guangdongensis* and 920 gene families in *G. elata* were completely missing, with 241 gene families absent in both species (Supplementary Fig. 10). The number of missing gene families was higher in the two fully mycoheterotrophic orchids than in the majority of the photosynthetic plant genomes investigated here, except for *Spirodela polyrhiza* and *Phoenix dactylifera* (Fig. 5 and Extended Data Fig. 2).

**Extended Data Fig. 3:**
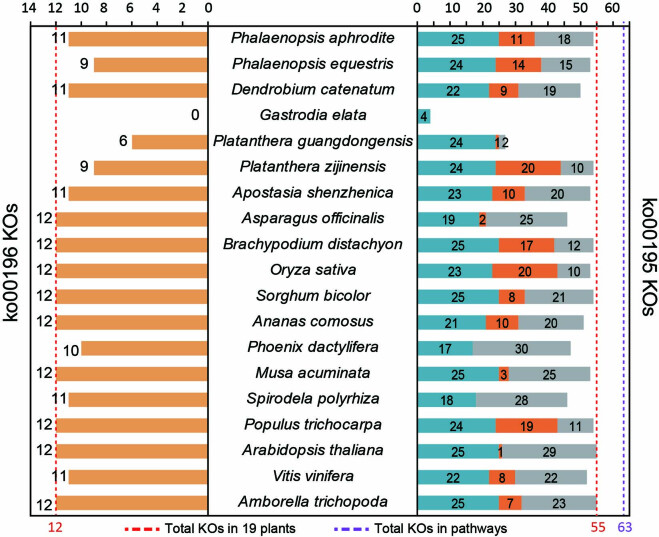
Orthologs in the pathways of Photosynthesis. Orthologs from 19 species in the pathways of Photosynthesis - antenna proteins (ko00196 for green plants, left) and Photosynthesis (ko00195, right). Bar colors from left to right: dark yellow, the KOs only found in nuclear genome in ko00196; cyan, the KOs that only found in nuclear genome in ko00195; orange, the KOs found in both nuclear and chloroplast genomes in ko00195; grey, the KOs only found in chloroplast genome in ko00195.

Further, we investigated functions of the missing gene families by performing Gene Ontology (GO) enrichment analyses of the missing genes in the genomes of *P. guangdongensis*, *G. elata*, *S. polyrhiza* and *Pho. dactylifera*. The genes missing in *S. polyrhiza* might be related to its aquatic habitat^32^ (Supplementary Table 17), while the missing genes in *Pho. dactylifera* are probably mainly due to the partial nature of its genome^33^ (Supplementary Table 18). Our GO enrichment analyses further show that the missing gene families in the two fully mycoheterotrophic orchids were highly similar to each other with respect to their GO terms, as many of the lost genes are involved in photosynthesis (Supplementary Tables 19 and 20), which is in line with their inability to perform photosynthesis. Analysing the photosynthesis pathways based on the Kyoto Encyclopedia of Genes and Genomes (KEGG) also shows that more photosynthesis-related orthologues are missing in *P. guangdongensis* and *G. elata* compared with species that can perform photosynthesis (Extended Data Fig. 3 and Supplementary Table 21). For example, compared with photosynthetic species that have at least nine antenna proteins (ko00196), *P. guangdongensis* and *G. elata* only have six and none, respectively. By analysing genes from both nuclear and chloroplast genomes, we found that *P. guangdongensis* has 27 genes and *G. elata* has 4 genes involved in the photosynthesis pathway (ko00195). In contrast, all the sequenced photosynthetic orchids have at least 50 genes functioning in the photosynthesis pathways, and *P. zijinensis*, a partial mycoheterotroph, even has 54 such genes. Further examining the chloroplast genomes of *P. guangdongensis* and *P. zijinensis*, we found that *P. guangdongensis* has a chloroplast genome of 88,060 bp with 60 genes, considerably smaller than that of *P. zijinensis* with a size of 151,858 bp and containing 128 genes. In addition, *G. elata* has a chloroplast genome with a size of only 35,304 bp and 28 genes^24^. Our results confirm that, similar to *C. australis* parasitically living on photosynthetic plants^30^, both fully mycoheterotrophic orchids studied here lost many genes involved in photosynthesis in the nuclear and chloroplast genomes (Supplementary Note 1, Supplementary Figs. 11–14 and Supplementary Table 22).

Because both *P. guangdongensis* and *G. elata* must have evolved a fully mycoheterotrophic lifestyle independently from initially mycoheterotrophic ancestors, our results suggest parallel evolution during the evolution of mycoheterotrophy. Interestingly, both the nuclear and chloroplast genomes of *G. elata* have lost more ‘photosynthetic’ genes than *P. guangdongensis* (Extended Data Fig. 3), suggesting that *G. elata* may have adapted to a fully mycoheterotrophic lifestyle more completely than *P. guangdongensis*. Indeed, the genus of *Gastrodia* includes about 90 species that are all fully mycoheterotrophic, with a wide geographic distribution^34^. In contrast, *Platanthera* has about 100 species, most of which are initial mycoheterotrophs (such as most orchids), except for a few partial mycoheterotrophs and three reported full mycoheterotrophs with narrow geographic distributions: *Platanthera saprophytica* in Borneo^35^ and *Platanthera fujianensis*^36^ and *P. guangdongensis*^20^ in Fujian and Guangdong, two neighbouring provinces in southeastern China. Therefore, species from *Gastrodia* probably adopted the full mycoheterotrophic lifestyle before their divergence, while full mycoheterotrophy in the genus *Platanthera* seems to have evolved independently from initial mycoheterotrophy several times.

In addition, the partially mycoheterotrophic *P. zijinensis* has lost 542 gene families, fewer than those missing in *P. guangdongensis* and *G. elata* but more than most other photosynthetic species, except for *Asparagus officinalis*, which has lost 662 gene families (Extended Data Fig. 2). GO enrichment analyses of the missing gene families in *P. zijinensis* and *A. officinalis* show that these genes are mainly genes involved in “macromolecule metabolic process” (Supplementary Tables 23 and 24).

As a partial mycoheterotroph, mature *P. zijinensis* absorbs carbohydrates from fungi when photosynthesis is not feasible. Considering gene loss being a common pattern during the evolution from initial to full mycoheterotrophy, partial mycoheterotrophs may have already lost some genes because of their ability to retrieve carbohydrates from fungi. *A. officinalis* has been known to be associated with an arbuscular mycorrhizal fungus (AMF), *Glomus intraradices*, and growing in an environment without the fungus reducing its biomass^37^. Although AMF symbiosis is prevalent in land plants, *A. officinalis* is among the best-responding plants to AMF association^38^ and it can produce a large white spear underground in a few weeks, suggesting that it is worth investigating whether *A. officinalis* is able to use carbohydrates provided by its associated AMF and whether gene loss in *A. officinalis* is correlated with its association with AMF.

### Gene loss and adaptation to mycoheterotrophy in *P. guangdongensis*

Although *P. guangdongensis* lost many genes mostly involved in photosynthesis, in line with its non-photosynthetic lifestyle, the loss or contraction of many other genes remains enigmatic with respect to the adaptation of *P. guangdongensis* as a fully mycoheterotrophic plant. In general, gene loss can be correlated with adaptive evolution in two ways: on the one hand, gene loss may result in phenotypes that would confer adaptive advantages to an organism; on the other hand, it might be a consequence of relaxed purifying selection following adaptive evolution^39^. As gene-family expansion and contraction (or loss) may be associated with adaptive evolution, we determined the expansion and contraction of orthologous gene families using CAFE 4 (ref. ^40^; Methods), to see if the size changes of some gene families have been significantly more rapid than expected under the neutral birth-and-death model on three different branches: (1) the branch before the divergence between *P. zijinensis* and *P. guangdongensis*; (2) the branch leading to *P. zijinensis*; and (3) the branch leading to *P. guangdongensis* (Fig. 2).

On the branch leading to *P. zijinensis* and *P. guangdongensis*, only eight and ten gene families significantly contracted and expanded, respectively (Supplementary Tables 25 and 26). On the branch leading to *P. zijinensis*, there are 7 significantly contracted gene families and 63 significantly expanded gene families (Supplementary Tables 27 and 28). Reversely, on the branch leading to *P. guangdongensis*, there are 28 significantly contracted gene families and 16 gene families that have expanded (Supplementary Tables 29 and 30). Interestingly, some of these significantly contracted gene families are related to several mycoheterotrophic characteristics found in *P. guangdongensis*. For example, two significantly contracted gene families in *P. guangdongensis* are ‘light-harvesting chlorophyll A/B binding proteins (LHCB)’ and ‘auxin efflux carriers’, which are of importance for fully mycoheterotrophic species to live in the darkness (see below).

**Fig. 6:**
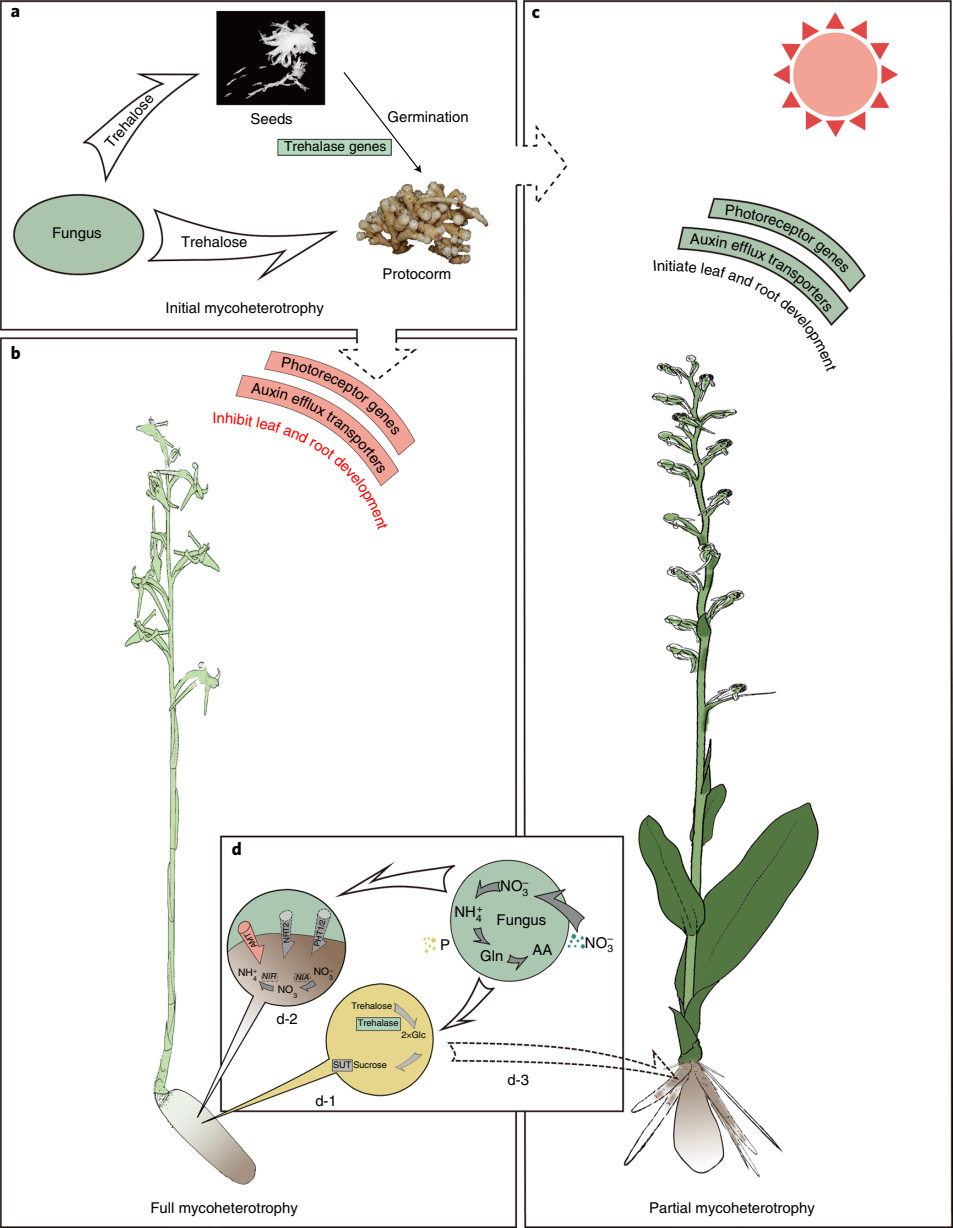
The potential molecular mechanisms of mycoheterotrophy in orchids. **a**, Initial mycoheterotrophy. The seeds of orchids do not have endosperms and their germination depends on absorbing carbohydrates, such as trehalose, from their associated fungi. Trehalose is a disaccharide in fungi that has similar roles to sucrose in plants. In contrast to other sequenced plant genomes, most orchid genomes have multiple copies of trehalase genes (Supplementary Table 34), which digest a molecule of trehalose into two molecules of glucose (d-1). The seeds develop into protocorms, which keep using the carbohydrates from fungi until they can perform photosynthesis. **b**, Full mycoheterotrophy. Leaflessness of *P. guangdongensis* may be related to the loss of most photoreceptor genes and auxin efflux transporters such as *PIN*s, because light signals are essential for leaf initiation and positioning through redistribution of auxin to the incipient primordia. The development of roots also relies on light signals and photosynthesis. In addition, transcription factors involved in root development, such as *CPC*, *TRY* and *ETC1*-like genes, are missing in *P. guangdongensis*, which may correlate with its rootless phenotype. **c**, Partial mycoheterotrophy. In contrast to full mycoheterotrophy, partially mycoheterotrophic orchids have similar numbers of photoreceptors and auxin efflux transporters, which are important for the development of leaves, to *Pha. equestris* and *D. catenatum*. *P. zijinensis* has all the transcription factors involved in root development as well as the *AGL12* genes. **d**, Obtaining nutrients from fungi. Fully mycoheterotrophic orchids keep expressing trehalase genes as protocorms to digest trehalose into glucose (Glc), which is further converted into sucrose and transported by SUTs throughout the plant body (d-1). Mycoheterotrophic orchids also have fewer nitrogen and phosphorus transmembrane transporters, such as AMT, NRT2 and PHT1/2, than *Pha. equestris* and *D. catenatum*. Furthermore, *NIA* and *NIR* genes are missing in the *G. elata* genome and have low expression in *P. guangdongensis*, suggesting that these orchids may only obtain nitrogen in the forms of ammonium (${{\mathrm{NH}_{4}^{+}}}$) and/or glutamine (Gln) and amino acid (AA) from fungi but cannot absorb nitrate (${{\mathrm{NO}_{3}^{-}}}$) from soil (d-2). As a partially mycoheterotrophic orchid, *P. zijinensis* can perform photosynthesis and obtains trehalose from its associated fungi (d-3).

**Extended Data Fig. 4:**
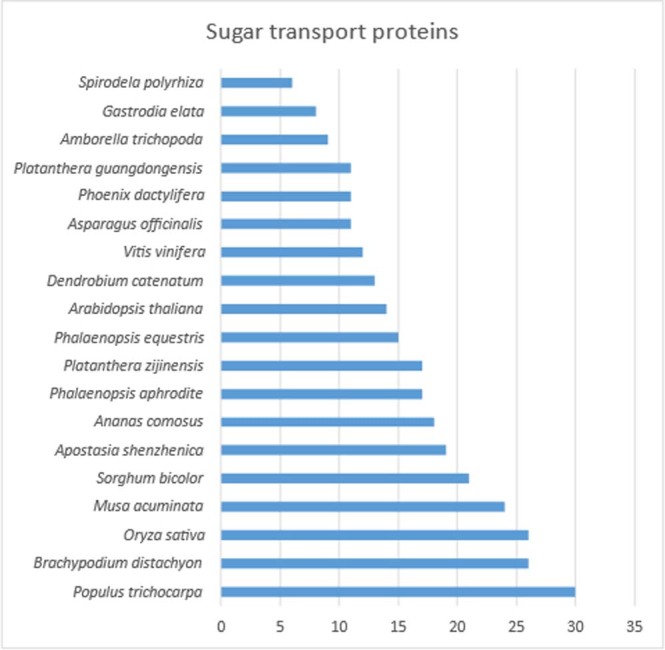
The sugar transport proteins. Number of sugar transport proteins in sequenced plant genomes.

Also, sugar transport proteins have been lost in *P. guangdongensis*, which may contribute to the mechanisms that help fully mycoheterotrophic orchids withhold benefits from their associated fungi. Indeed, the two fully mycoheterotrophic orchids, *P. guangdongensis* and *G. elata*, have the smallest number of sugar transport proteins among all the sequenced orchids (Extended Data Fig. 4). All photosynthetic orchids are initial mycoheterotrophs, for their dust-like seeds do not have endosperms and rely on symbiotic fungi to provide them with carbon compounds and nutrients for germination (Fig. 6a). In return, after becoming able to perform photosynthesis, photosynthetic orchids export carbohydrates to the symbiotic fungi for the further continuous exchange of nitrogen and phosphorus (Fig. 6c). This is a common understanding of the symbiotic mutualism between photosynthetic orchids and symbiotic fungi, and a similar mutualistic relationship also exists between trees and ectomycorrhizal fungi^41^. However, since full mycoheterotrophs live completely depending on carbohydrates from the associated fungi, limiting the carbon flux from themselves to the associated fungi by reducing the number of sugar transporters could be beneficial (Fig. 6b).

### Loss of light-harvesting genes

Although the inability to perform photosynthesis is a unique feature of full mycoheterotrophs, the evolutionary forces acting on the loss of photosynthetic genes, or a subset thereof, remain elusive. The CAFE analysis shows that the light-harvesting *LHCB* gene family has lost a significant number of genes on the branch leading to *P. guangdongensis*. Specifically, there is only one *LHCB* gene found in *P. guangdongensis* while *LHCB* genes are completely missing in *G. elata* (Supplementary Table 29). This would suggest that the loss of *LHCB* genes may be driven by relaxation selection during the evolution of full mycoheterotrophy. Although it is not clear how the loss of *LHCB* genes and photosynthesis could be beneficial by itself, some evidence in partially mycoheterotrophic orchids suggests that the loss of photosynthesis may lead to a higher degree of mycoheterotrophy, on par with the effects of limited light availability on the degree of mycoheterotropy^42^. Some green, partially mycoheterotrophic orchids, such as *Cephalanthera damasonium*, have natural non-chlorophyllous albinos that cannot perform photosynthesis, even under sunlight^16^. Those albinos generally have lower fitness than their green counterparts^16^, but they may outperform their green counterparts in a low-light environment where they only absorb carbohydrates from the associated fungi rather than struggle to perform photosynthesis, because the loss of photosynthesis confers a higher degree of mycoheterotrophy to such albinos than their green counterparts. After photosynthesis is “switched-off” through losing key components such as *LHCB*, the loss of other photosynthetic genes may be likely due to relaxed selection pressure once the obliged association has been established. Although the CAFE results discussed above support selection as a force that drives the loss of (a part of) photosynthesis during the evolution to full mycoheterotrophy of *P. guangdongensis*, further comparisons with other full mycoheterotrophs are necessary to generalize if the loss of photosynthesis is adaptive during the evolution of full mycoheterotrophy.

### Loss of photoreceptors

**Extended Data Fig. 5:**
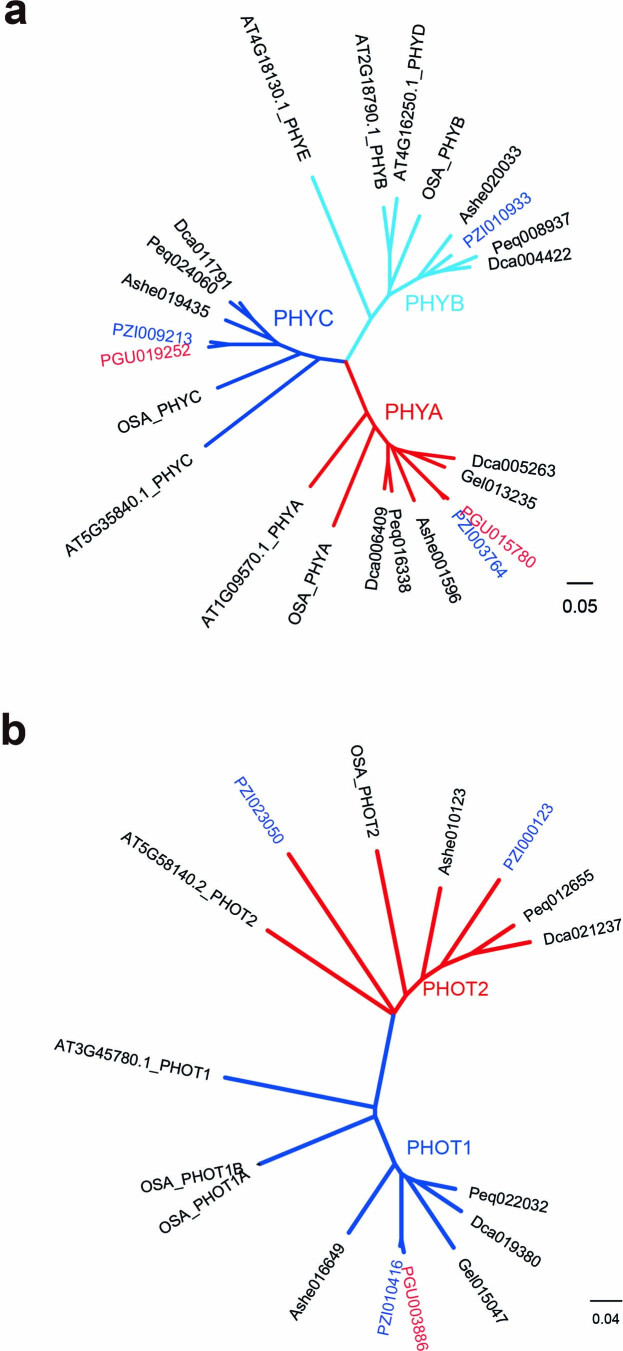
Phylogenetic analysis of phytochromes (PHY) and phototropins (PHOT) in different orchids. a. PHY. b. PHOT. AT, *Arabidopsis thaliana*; OSA, *Oryza sativa*; Ashe, *Apostasia shenzhenica*; Gel, *Gastrodia elata*; Dca, *Dendrobium catenatum*; Peq, *Phalaenopsis equestris*; PZI, *Platanthera zijinensis*; PGU, *Platanthera guangdongensis*.

Living in a low-light environment may have resulted in the loss of photoreceptors. Indeed, *P. guangdongensis* and *G. elata*, compared with partially and initially mycoheterotrophic orchids, have lost many photoreceptor genes (Supplementary Table 31). Orchids that can perform photosynthesis, such as *P. zijinensis*, *Pha. equestris* and *D. catenatum*, usually have a total of eight to nine copies of photoreceptor genes, including cryptochromes (*CRY*) and phototropins (*PHOT*) for ultra-blue and blue light, as well as phytochromes (*PHY*) for red and far-red light. However, neither *P. guangdongensis* nor *G. elata* has *CRY*, suggesting that they only have a limited response to blue light, which is one of the main contributors to photosynthesis. *P. guangdongensis* and *G. elata* have both retained *PHOT1* and *PHYA* but lost *PHOT2* and *PHYB*, consistent with the low-light environment of their living habitat (Extended Data Fig. 5). Compared with *PHOT2*, which mainly responds to blue light of high intensity, *PHOT1* responds to a broad range of light intensity from weak to strong^43^. *PHYA* and *PHYB* are sensitive to a broad spectrum of light from UV-A to far-red light^44^. In addition, a high-level expression of *PHYA* has been observed in seedlings growing in a dark environment, suggesting that *PHYA* is important for seed germination and seedling development in closed-canopy forests with low light^44^. Apparently, the loss of photoreceptors that mainly respond to light of high intensity could be tolerated by the two full mycoheterotrophs, *G. elata* and *P. guangdongensis*. After all, they spend most of their life underground in a fully dark environment. Only before inflorescence do they grow out from the underground and are exposed to a low-light environment.

Although photoreceptors can regulate flowering time, sense day length and maintain the circadian rhythm of plants, it has been shown that genes involved in circadian clock and flowering-time regulation tend to be lost in heterotrophic species, as has also been observed for *G. elata* and *C. australis*^45^. Photoreceptors also mediate various physiological and developmental processes of plants, such as phototropism, leaf expansion, chloroplast movement and neighbour perception^44^, suggesting that the loss of some photoreceptor genes might have had a cascading effect on the general biological response to light, such as leaf and root development. Therefore, the loss of some photoreceptors may have contributed to the evolution of joint traits for full mycoheterotrophy along with the loss of photosynthesis^46^, further reinforcing the dependence on the carbohydrates from fungi (Fig. 6b,d).

### Darkness inhibits leaf development

**Extended Data Fig. 6:**
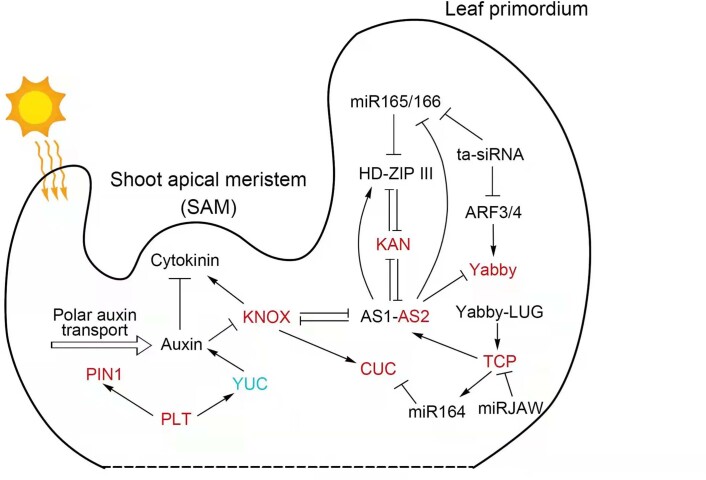
The regulatory network involved in *Arabidopsis* leaf initiation and development, and *P. guangdongensis* and *G. elata* contraction (loss) genes of involved in leaf initiation and development. The regulatory network was modified according to three review papers^6,7,9^. The transition of cells in the shoot apical meristem (SAM) from a pluripotent fate to a determinate fate is necessary for plant leaf initiation. SAM is maintained by the class I KNOX homeodomain transcription factor through the activation of cytokinin signalling and repression of asymmetric leaves1 (AS1) and AS2, which are involved in organogenesis. The leaf organ initiates from the sites where the expression of KNOX is downregulated and auxin maxima are established via polar localization of *PIN1*, the auxin efflux carrier. Light is necessary for leaf initiation. Without light, polar localization of *PIN1* is lost, and leaf production ceases^7^. The PLETHORA (PLT) transcription factors PLT3, PLT5, and PLT7, have been reported to be required for proper expression of *PIN1*^10^ and YUC1/4^11^. AS1 and AS2 form a complex and repress the expression of *Yabby*, KAN, and miR165/166. *Yabby* gene expression is essential for the switch of the SAM program to the leaf-specific program^9^. In addition, Yabby interacts with LUG to promote the expression of *Class II TCP* genes which are involved in lamina development. The *AS1* and *CUC* genes are regulated by *Class II TCPs* to control lamina development^7^. The *Arabidopsis* genes regulating leaf initiation and development in this figure were used as queries to identify orthologous genes in the genomes of *P. guangdongensis*, *P. zijinensis*, *G. elata*, *A. shenzhenica*, *D. catenatum*, and *Pha.* *equestris* (Supplementary Table 30). The genes indicated in red are lost or contracted in *P. guangdongensis* and *G. elata*, whereas those in blue are lost in *G. elata*. ARF: auxin-responsive factor; CUC: CUP-SHAPED COTYLEDON; KAN: KANADI; KNOX: KNOT-TED-like homeobox (STM, BP/KNAT1, KNAT2, KNAT6); LUG: LEUNIG; PIN1: PIN-FORMED 1; YUC: YUCCA.

**Extended Data Fig. 7:**
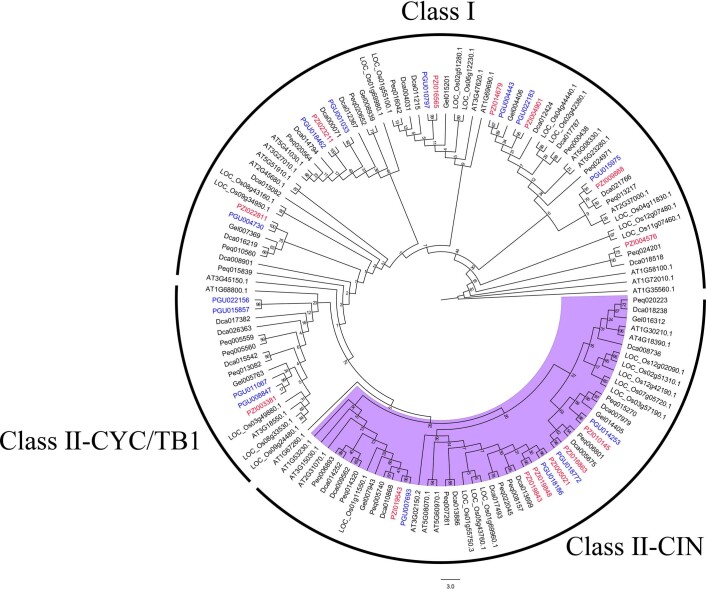
The phylogenetic tree of the TCP transcription factor families. The TCP sequences in different plant species were isolated based on BLAST analysis using *Arabidopsis TCP* genes as queries (Supplementary Table 30). The clades were labelled by subfamily category. The purple highlight is the Class II-CIN clade. The blue and red fonts represent the sequences of *P. guangdongensis* (PGU) and *P. zijinensis* (PZI), respectively. The black font represents the sequences of rice (labelled LOC), *Arabidopsis* (AT), *G. elata* (Gel), *D. catenatum* (Dca), and *Pha.* *equestris* (Peq). Bootstrap values are shown on each node.

Leaflessness is a significant feature of the fully mycoheterotrophic *P. guangdongensis* and *G. elata*, compared to the partially mycoheterotrophic species *P. zijinensis* and the other initially mycoheterotrophic orchids. The genomes of *P. guangdongensis* and *G. elata* have still retained most of the genes that are well-known for regulating leaf initiation and development in *Arabidopsis*, including auxin synthetic/responsive genes and transcription factors. However, in the genomes of *P. guangdongensis* and *G. elata*, some of these gene families have fewer copy numbers than in the chlorophyllous orchids (Fig. 6b and Supplementary Table 31). For example, *P. guangdongensis* and *G. elata* each only have one *PIN1*, whereas other chlorophyllous orchids mostly have three *PIN1* genes. The leafless character of *P. guangdongensis* may be linked to the loss of photoreceptor genes because a light signal is essential for leaf initiation and positioning by regulating the distribution of auxin (Extended Data Fig. 6). Auxin can trigger organogenesis at the shoot apical meristem (SAM), and it depends on the expression of *PIN1* genes at the SAM to redistribute auxin generated at the meristem dome to the incipient primordia^47–49^. As dark treatment can affect the subcellular localization of *PIN1* and cease leaf initiation in tomato^50^, the loss of photoreceptor genes, together with further contractions of genes involved in leaf initialization and development, may eventually have led to the leafless phenotype (Fig. 6b, Supplementary Note 2 and Extended Data Figs. 6 and 7).

### Darkness inhibits root development

Both *P. guangdongensis* and *G. elata* have a tuber without roots. Light signalling is important to induce the development of roots by providing carbohydrates and auxin^51^. Indeed, comparing the transcription factor genes that are involved in root development between the two *Platanthera* genomes (Supplementary Table 32), we found that most of the transcription factor genes were maintained in both genomes, except for *CAPRICE*- (*CPC*-), *TRIPTYCHON-* (*TRY-*) and *ENHANCER OF TRY AND CPC-*like (*ETC1-*like) genes, which are missing in *P. guangdongensis*. Each of these three genes encodes a protein with an myeloblastosis-like deoxyribonucleic acid-binding (DNA-binding) domain that lacks a transcriptional activation domain. These three genes have overlapping functions during root hair differentiation in *Arabidopsis*^52^, so their loss may also underlie rootlessness in *P. guangdongensis* (Fig. 6b).

In addition, MADS (MCM1, AGAMOUS, DEFICIENS, SRF)-box transcription factors are among the most important regulators of plant development (Table 1 and Supplementary Tables 33 and 34). It has been reported that *AGL12* genes are involved in root cell differentiation in *Arabidopsis*^53^. Also, the carnivorous aquatic plant *Utricularia gibba* from the order Lamiales does not bear true roots and has lost the MADS-box *AGL12* genes^54^. Different from epiphytic orchids such as *Pha. equestris* and *D. catenatum*, which have no *AGL12* genes and have lost the ability to develop true roots for terrestrial growth, the terrestrial orchid *A. shenzhenica* has an *AGL12* gene and develops a complex underground root system^22^. Thus, although it requires further verification, we assume that the presence of *AGL12* genes might be necessary for the development of a root system in terrestrial orchids. Phylogenetic analysis of the type II MADS-box genes indicated that *P. zijinensis*, as a terrestrial orchid with a root system like *A. shenzhenica*, harbours an intact *AGL12* gene (Supplementary Fig. 15 and Fig. 6c), while *P. guangdongensis* has no root and only contains a pseudogenenized *AGL12* gene with a truncated MADS-box domain (Supplementary Table 34), suggesting an association between the loss of *AGL12* and the ability to develop a terrestrial root system in *P. guangdongensis* (Fig. 6b).

**Table 1:** Number of MADS-box genes in the orchid *P. zijinensis*, *P. guangdongensis*, *A. shenzhenica*, *D. catenatum* and *Pha. equestris* genomes

Category	*P. zijinensis*		*P. guangdongensis*		*A. shenzhenica*		*D. catenatum*		*Pha. equestris*	
	Functional	Pseudo	Functional	Pseudo	Functional	Pseudo	Functional	Pseudo	Functional	Pseudo
Type II (Total)	29	2	27	3	27	4	35	11	29	1
MIKC^c^	27	0	25	2	25	3	32	9	28	1
MIKC*	2	2	2	1	2	1	3	2	1	0
Type I (Total)	14	1	16	1	9	0	28	1	22	8
Mα	11	1	14	0	5	0	15	1	10	6
Mβ	0	0	0	0	0	0	0	0	0	0
Mγ	3	0	2	1	4	0	13	0	12	2
Total	43	3	43	4	36	4	63	12	51	9
Reference	This study		This study		Zhang et al.^23^		Zhang et al.^22^		Cai et al.^21^	

Note: MIKCc, Canonical type II MADS-box genes; MIKC*, Type II MADS-box genes with gene length usually longer than MIKCc genes; Mα, Subgroup a of type I MADS-box genes; Mβ, Subgroup b of type I MADS-box genes; Mγ, Subgroup g of type I MADS-box genes.

### Nutrition absorption in the dark

Compared with autotrophic orchids, full mycoheterotrophs such as *P. guangdongensis* and *G. elata* have lost their ability to do photosynthesis and can only complete their life cycle by absorbing carbohydrates through associated fungi. Indeed, pelotons, that is, the typically coiled hypha in cortical cells of host plants, have been observed in protocorms^55^ as well as in mature mycoheterotrophic orchids^56^. The host cells can disintegrate the pelotons and hence obtain nutrients from the fungi^57^. Most well-studied mycoheterotrophic orchids are related to ectomycorrhizal fungi, in which trehalose is (one of) the carbohydrate(s) transferred from ectomycorrhizal fungi to mycoheterotrophic orchids^1,56,58–60^. Like sucrose in plants, trehalose is a disaccharide composed of two molecules of glucose and acts as a storing and transporting carbon compound in fungi. For instance, ectomycorrhizal fungi presumably synthesize trehalose and transport trehalose towards soil-growing hyphae^61^ after absorbing glucose from photosynthetic plants, such as trees. The trehalose stored in fungal hyphae then becomes available as carbon resource for mycoheterotrophic orchids. Alternatively, sucrose has been suggested to be transported from fungus to *G. elata*, because high sucrose concentration and two sucrose transporter (SUT)-like genes are highly expressed in tubers at the early stage of fungus colonization in *Gastrodia*^62^.

Interestingly, we observed multi-copy trehalase genes in *P. guangdongensis* (2), *P. zijinensis* (2), *G. elata* (4), *D. catenatum* (3), *Pha. aphrodite* (2) and *Populus trichocarpa* (3), compared to their single-copy status in other investigated plant genomes (Supplementary Table 35). Trehalose is an important signalling chemical in embryo development and response to abiotic stress^63^. However, plants with high accumulation of trehalose by ectopic-expressing heterologous fungal or bacterial enzymes resulted in abnormal development^64^, so most angiosperms keep trehalose at a low level^65^.

**Extended Data Fig. 8:**
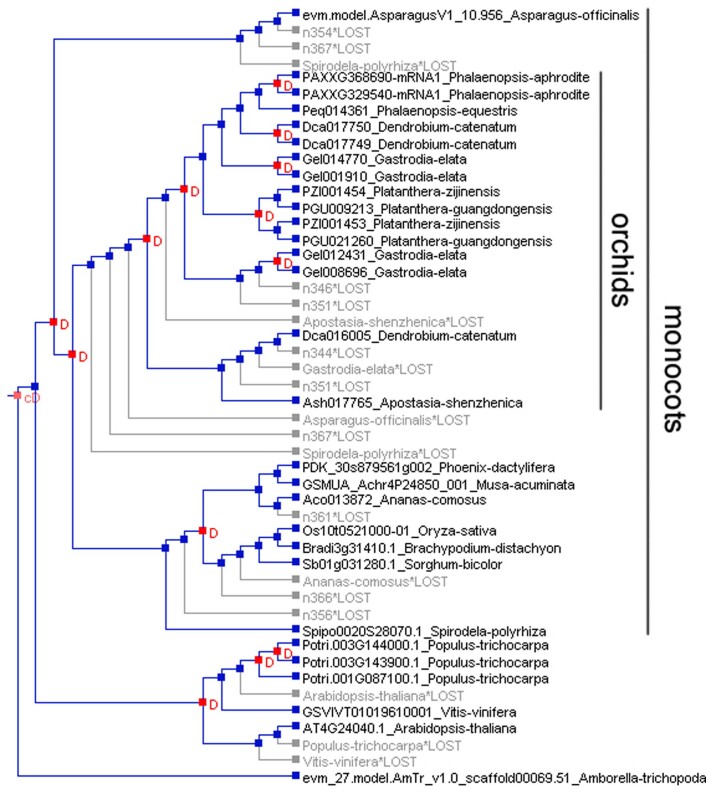
Phylogenetic tree of trehalase genes. Phylogenetic tree of trehalase genes in orchids and monocots.

Consistently, species that can use carbohydrates from fungi, such as orchids and *Po. trichocarpa*, tend to have multiple copies of trehalase genes via independent duplication events (Extended Data Fig. 8). We further compared the expression of trehalase genes in the mature plant bodies of *P. guangdongensis* and *P. zijinensis* using *D. catenatum* as a control and observed that the trehalase genes are upregulated in both *P. guangdongensis* and *P. zijinensis* (Supplementary Fig. 16), suggesting that mature *P. guangdongensis* and *P. zijinensis* can both efficiently convert the obtained trehalose to glucose by trehalase to keep the levels of trehalose low in the plant, especially when pelotons disintegrate and release all the nutrients in the cortical cells (d-1 and d-3 in Fig. 6d).

As expected, the fully mycoheterotrophic *P. guangdongensis* shows a higher level of expression of the trehalase genes than does the partially mycoheterotrophic *P. zijinensis* (Supplementary Fig. 16), indicating that trehalose is at least one of the main carbohydrates that *P. guangdongensis* obtains from fungi (d-1 in Fig. 6d). Further, we examined the expression patterns of *SUT* genes in various organs in *P. guangdongensis* using transcriptome analysis. The results showed that all *SUT* genes of *P. guangdongensis* were predominantly expressed in tuber, stem and flower (Supplementary Fig. 17). Considering that plants transport sucrose rather than glucose throughout their bodies, we propose that one molecule of trehalose from fungi is firstly digested into two molecules of glucose via trehalase, and then the two glucose molecules are synthesized into a sucrose molecule and further transported by SUTs (d-1 in Fig. 6d). This explains the multi-copy status and the expression profile of trehalase genes and the observation of high sucrose concentration in tubers and the expression profile of *SUT* genes (Supplementary Figs. 16 and 17).

**Extended Data Fig. 9:**
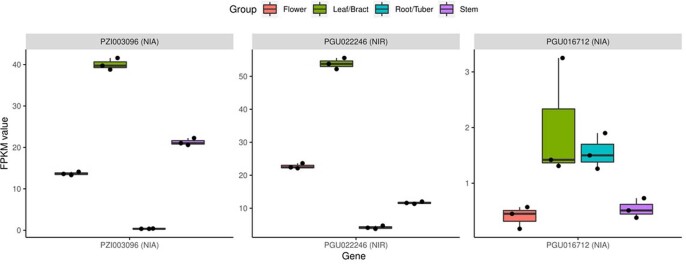
Expression patterns of *NIA* and *NIR* genes. Expression patterns of *NIA* and *NIR* genes in various organs of *P. zijinensis* and *P. guangdongensis*, which are performed in three replicates. The means of expression value are shown above the bar differ; the error bars indicate the ±SDs of three biological replicates.

**Extended Data Fig. 10:**
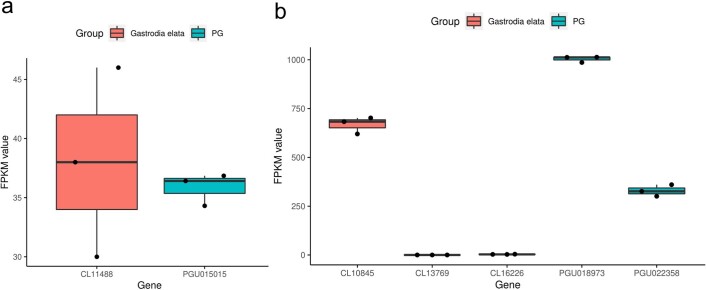
The expression of glutamine synthetase (GS) and glutamate synthase (GOGAT) in tubers of *P. guangdongensis* and *G. elata*. a. The expression of GS; b. The expression of GOGAT. The means of expression value are shown above the bar differ; the error bars indicate the ±SDs of three biological replicates.

Aside from obtaining carbohydrates from associated fungi, *P. guangdongensis* may also take up nitrogen (N) and phosphate (P) from fungi or through specialized transporters from the symbiotic interface of fungi and plants. However, the mechanisms for nutrient transfer and the nutrient forms obtained from fungal cells are still unclear^4,66^. Compared to the partially mycoheterotrophic *P. zijinensis*, the *G. elata* genome has lost both nitrate reductase (*NIA*) genes and nitrite reductase (*NIR*) genes (Supplementary Table 36). The *P. guangdongensis* genome has only lost the *NIA* genes, but the expression of its *NIR* genes remain low in stems and tubers (Supplementary Table 36 and Extended Data Fig. 9), suggesting that they may not directly utilize nitrate from the soil as other orchids. In addition, there are fewer genes encoding ammonium transporters (*AMT*) in *P. guangdongensis* and *G. elata* than in other sequenced orchids, while high-affinity nitrate transporters (*NRT2*) and phosphate transporters (*PHT*) are completely missing in *P. guangdongensis* and *G. elata* (Supplementary Table 36). However, both *P. guangdongensis* and *G. elata* have the glutamine synthetase–glutamate synthase pathway to incorporate ammonium into amino acids (Extended Data Fig. 10). These results hence suggest that *P. guangdongensis* and *G. elata* may mainly obtain N and P from fungi and that the N compounds acquired from fungi might be glutamine or ammonium but not nitrate (d-2 in Fig. 6d). This finding is somewhat consistent with previous studies showing that glutamine and ammonium may be the preferred forms of N released by fungi^67,68^.

### Mycoheterotrophy in mature orchids as a continuation of the protocorm stage

Although there is a general understanding that the evolution of mycoheterotrophy follows a path from autotrophy to initial and partial mycoheterotrophy, and eventually to full mycoheterotrophy^2^, the evolutionary forces behind the process remain unknown. Because all chlorophyllous orchids are initially mycoheterotrophs, their growth process involves a mycoheterotrophic period of seed germination and the protocorm stage (Fig. 6a) and an autotrophic period when the seedling can perform photosynthesis (Fig. 6c). The growing stages of orchids indicate that all orchids have the genetic toolkits for living, at least temporarily, as a mycoheterotrophic plant, so utilization of carbohydrates from associated fungi in mature orchids is probably a continued strategy that some species adopt from the protocorm stage to adapt to an environment where light is insufficient for photosynthesis (Fig. 6b). The transition from mycoheterotrophy to autotrophy during the development of orchids must include changes in the expression of some genes related to the two different modes of nutrition. Our gene expression analysis of trehalase genes in organs from mature *P. guangdongensis* and *P. zijinensis* plants show that the upregulation of the trehalase genes in *P. guangdongensis* may play a key role in switching between autotrophy and mycoheterotrophy. Hence, investigating the expression patterns of the trehalase genes along the growth of orchids may shed light on how some orchids become full mycoheterotrophs.

**Fig. 7:**
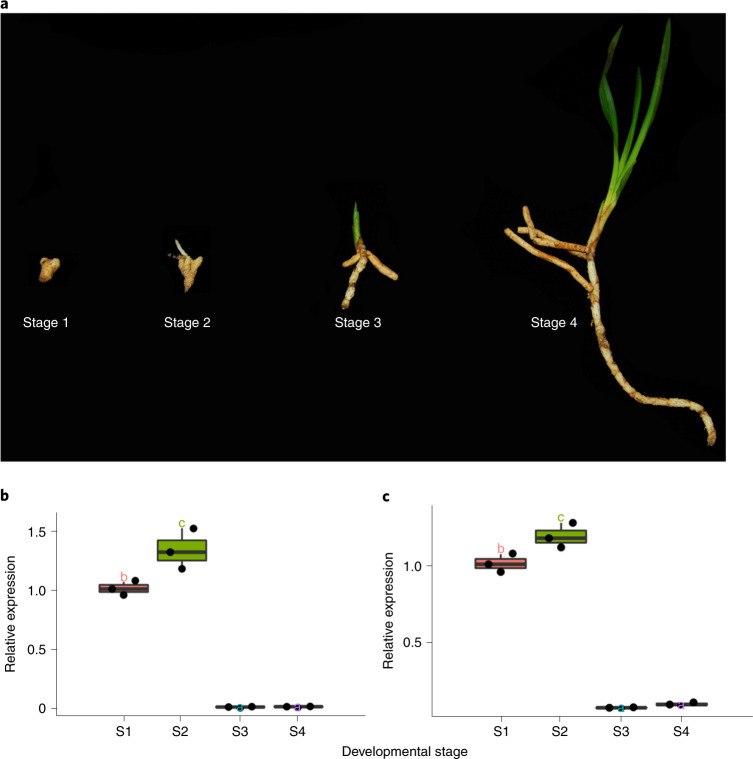
Expression patterns of trehalase in different stages of *C. goeringii.* **a**, Different development stages including rhizome (stage 1), rhizome with branches (stage 2), small seedling (stage 3) and older seedling (stage 4), abbreviated as S1 to S4, respectively. Expression patterns of trehalase genes *c249696_g1_i4* (*CL09234* (ref. ^69^)) (**b**) and *c232459_g1_i2* (*CL20246* (ref. ^69^)) (**c**) in different stages. The line in the middle of a box represents the median value and the top and bottom borders of the boxes denote the 75th and 25th percentiles, respectively. The upper and lower bars show the largest value within 1.5 times the interquartile range above the 75th percentile and the smallest value within 1.5 times the interquartile range below the 25th percentile, respectively. A dot shows the corresponding data points. Different letters represent significant differences by one-way ANOVA with Duncan’s post hoc test (*P* < 0.05).

To this end, we collected samples of the orchid *Cymbidium goeringii* at different developmental periods, including rhizome (protocorm) as stage 1, rhizome with branches as stage 2, young seedlings as stage 3 and older seedlings as stage 4 (Fig. 7). The first two samples were considered to represent the mycoheterotrophic stage (stages 1 and 2), while the latter two samples were considered to represent the autotrophic stage (stages 3 and 4) (Fig. 7a). The real-time quantitative reverse transcription PCR (qRT-PCR) analyses show that the expression level of two trehalase genes in *C. goeringii*^69^ (Supplementary Table 37) is upregulated in the samples from the mycoheterotrophic stage and downregulated in the samples from the autotrophic stage (Fig. 7b,c). Our results hence illustrate that downregulation of trehalase genes is involved in the transition from mycoheterotrophy to autotrophy during the growth of a photosynthetic orchid, indicating that the transition is correlated with changes of gene expressions.

Indeed, some orchid species that are believed to be autotrophic have turned out to be mixotrophic, at least during a specific period after the protocorm stage^70^, suggesting the ease of switching between autotrophy and mycoheterotrophy in Orchidaceae through the regulation of gene expression. Therefore, the lifestyle of fully mycoheterotrophic orchids is supported by the continuous, high expression of trehalase genes and symbiosis with orchid mycorrhiza-accumulated trehalose. Considering that the dust seeds of orchids must live in symbiosis with fungi to germinate successfully, that could also explain why plant species belonging to Orchidaceae has a higher frequency of recurrence of mixotrophs and full mycoheterotrophs than other plant lineages^3^. In summary, our results suggest that terminating or reversing the transition from mycoheterotrophy to autotrophy and staying at the protocorm stage may be one of the decisive events on the evolutionary path from initial to full mycoheterotrophs in Orchidaceae.

### Into darkness

Although it remains unknown why some orchids have become full mycoheterotrophs, our comparative analyses of the *P. guangdongensis* and *P. zijinensis* genomes and other sequenced orchid genomes suggest that the evolution of full mycoheterotrophy in orchids may be an adaptation to occupy specific biological niches without light, as previous studies^1,3,71,72^ have shown. The seeds of nearly all orchids do not have endosperms and need a protocorm stage dependent on the associated fungi for their supply of nutrients (that is, the mycoheterotrophic stage) during their development. Then, as the protocorm grows and develops leaves, the orchid transitions into the autotrophic stage. If mutational changes can reverse the transition from mycoheterotrophy to autotrophy in the ancestor of a fully mycoheterotrophic orchid, then they may enable the ancestor to become a mixotroph that could survive in an environment with feeble light by reaching for alternative carbon sources from associated fungi. In fact, some features of full mycoheterotrophic orchids could be observed already in achlorophyllous variants of a mixotrophic orchid, *C. damasonium*^16^. The nutrition supplies from associated fungi might confer advantages to the mixotrophic orchid to explore new biological niches atypical for photosynthetic plants, such as expanding into the deep forest where light is scarce. Along the evolution of full mycoheterotrophy, the ancestor of a fully mycoheterotrophic orchid, according to the genomes of *P. zijinensis* and *P. guangdongensis*, may be a mixotroph that could sustain the expression of trehalase to hijack trehalose from the associated fungi as energy for its life cycle. While the mixotrophic ancestor has further adapted to the dark environment, losing genes for light response and photosynthesis as well as terminating the development of leaves and roots may have eventually given birth to the fully mycoheterotrophic orchid (Fig. 6).

In conclusion, by sequencing and analysing the genomes of the partially and fully mycoheterotrophic orchids *P. zijinensis* and *P. guangdongensis*, we reveal not only the potential molecular basis underlying important mycoheterotrophic traits, but also nutrient supplement mechanisms in the early and later stage of mycoheterotrophic growth, providing insights into the evolution of mycoheterotrophic plants.

## Methods

### Sample preparation

For genome sequencing, total DNA was extracted from multiple individuals of *P. zijinensis* in a wild population and the multiple individuals of *P. guangdongensis* in a wild population, using a modified cetyltrimethylammonium bromide protocol. Three replicates of tissues including tuber and roots, stems, leaves and flowers from three *P. zijinensis* individuals in a wild population and flowers, bracts and tubers from three *P. guangdongensis* individuals in a wild population were sampled for transcriptome sequencing. Total RNA was extracted from using RNAprep Pure Plant Kit (Tiangen Biotech) following the manufacturer’s instructions. Subsequently, total RNA was qualified and quality-checked using a Nano Drop and Agilent 2100 bioanalyzer (Thermo Fisher Scientific). Libraries were constructed using the mRNA-Seq Prep Kit (Illumina) and then sequenced by the Illumina HiSeq 4000 platform.

### PacBio library construction and sequencing

To construct genomic libraries (SMRTbell libraries) for PacBio long-read sequencing, high-molecular-weight genomic DNA was sheared into fragments of approximately 20 kb. Then, large-fragment genomic DNA was concentrated with AMPure PacBio beads and used for SMRTbell preparation according to the manufacturer’s specifications (Pacific Biosciences). The libraries were constructed and sequenced by the PacBio Sequel sequencing platform (Pacific Biosciences). In total, nine SMRT cells generated 441.17 Gb and 15 SMRT cells generated 414.21 Gb of sequencing data to assemble the *P. zijinensis* and *P. guangdongensis* genomes, respectively.

### Genome size estimation and sequence assembly

To estimate the genome size of *P. zijinensis* and *P. guangdongensis*, we used reads from paired-end libraries to determine the distribution of *k*-mer values by jellyfish v.2.1.4 (ref. ^73^) and genomeScope^74^. According to the Lander–Waterman theory^75^, genome size can be determined by the total number of *k*-mers divided by the peak value of the *k*-mer distribution. Given the high frequency of the first major peak in the *k*-mer distribution, we found that the heterozygosity rate in *P. zijinensis* and *P. guangdongensis* was very high, which may come from population diversity (Supplementary Figs. 1 and 2). With the peak as the expected *k*-mer depth and the formula genome size equals total *k*-mer/expected *k*-mer depth, the sizes of the *P. zijinensis* and *P. guangdongensis* haploid genomes were estimated to be 4.15 Gb and 4.27 Gb, respectively. We used Canu^76^ to correct the Pacbio subreads and used flye (v.2.4.2 release)^77^ to assemble the genome. Pilon v.1.22 (ref. ^78^) was used to correct indel and single-nucleotide polymorphisms errors in the assembly results. To deal with the high heterozygosity in the two *Platanthera* genomes, we used trimDup from the Rabbit Genome Assembler package (https://github.com/gigascience/rabbit-genome-assembler) to remove redundant contigs based on the *k*-mer occurrence frequency calculated by jellyfish v.2.1.4.

### HiC library preparation, sequencing and assembly of the chromosome

Approximately 5 g of leaves from *P. zijinensis* and 5 g of bracts from *P. guangdongensis*, respectively, were fixed in 1% formaldehyde for library construction. According to a previously described method^79^, cell lysis, chromatin digestion, proximity-ligation treatments, DNA recovery and subsequent DNA manipulations were performed. The MboI or DpnII enzyme was used to restrict chromatin digestion. The HiC library was sequenced on the Illumina HiSeq X platform for 150 bp paired-end reads. The HiC reads were aligned to the draft assembly using the Burrows-Wheeler Alignment aln algorithm^80^ with default parameters, and the quality was then assessed using HiC-Pro v.2.8.0 (http://github.com/nservant/HiC-Pro). Invalid interaction pairs, including self-circle ligation, dangling ends, PCR duplicates and other potential assay-specific artefacts, were discarded. The unique valid interaction pairs (nonredundant, true ligation products) were uniquely mapped onto the draft assembly contigs. The locations and directions of the contigs were determined by 3D-DNA (v.180922) preliminarily, with default parameters. To prevent excessive interruption, the result of the first iteration of 3D-DNA was used as input for Juicerbox (v.1.11.08; https://github.com/aidenlab/Juicebox/wiki/Download).

### Repeat prediction

Repbase^81^ was used to find repeats using RepeatProteinMask^82^ and RepeatMasker^82^. RepeatModeler was used to build the de novo repeats. Redundancies were then filtered out, and RepeatMasker was used to identify the positions of repeats. Through structural features, LTR_FINDER software^83^ and TRF software^84^ were used to find LTRs and tandem repeats, respectively.

### LTR insertion-time analysis

We used LTRharvest (parameters: minlenltr, 100; maxlenltr, 5,000 and maxdistltr, 25,000) and LTR_Finder v.1.07 (parameters: L, 5,000; l, 100; E) to find the intact LTR transposon elements in the two *Platanthera* genomes and integrated their prediction results through LTR_retriever^85^. To estimate the substitution rate for *Platanthera*, we employed MUMmer v.4.0.0 (ref. ^86^) to compare the genomic sequences of *P. zijinensis* and *P. guangdongensis* and find the collinear blocks between the two species. EMBOSS v.6.6.0 distmat programme^87^ with Jukes–Cantor as substitution model was then used to calculate the genetic distance between the sequences of the collinear blocks. The formula *r* = *d*/2*t* was used for substitution-rate calculation, with *r* for the substitution rate, *d* for the genetic distance and *t* for the divergence time of two *Platanthera* species estimated by MCMCTree (Fig. 2). The estimated substitution rate of *P. zijinensis* and *P. guangdongensis* was 1.65e-8 substitutions per site per Ma, which was fed into LTR_retriever to calculate the insertion time of LTRs in *P. zijinensis* or *P. guangdongensis*.

### Gene and non-coding RNA prediction

MAKER^88^ was used to generate a consensus gene set based on de novo prediction, homology annotation with BUSCO v.5 (ref. ^25^) and other sequenced angiosperms and RNA-seq prediction (Supplementary Table 11). These results were integrated into a final set of 24,513 and 22,559 protein-coding genes of *P. zijinensis* and *P. guangdongensis* for annotation, respectively. *P. zijinensis* and *P. guangdongensis* were found to have longer average messenger RNA length and intro length than most other sequenced plants (Supplementary Fig. 18 and Supplementary Table 38). We then generated functional assignments of the *P. zijinensis* and *P. guangdongensis* genes by aligning their protein-coding regions with sequences in public protein databases, including KEGG^89^, Swiss-Prot^90^, TrEMBL^91^ and InterPro^92^ (Supplementary Table 12). The genes with KEGG annotations were mapped to the corresponding KEGG pathways using Pathview^93^.

Transfer RNA genes were identified via tRNAscan-SE^94^. For ribosomal RNA identification, we downloaded *Arabidopsis* rRNA sequences from the National Center for Biotechnology Information (NCBI) and aligned them with the *P. zijinensis* and *P. guangdongensis* genomes to identify possible rRNAs. Additionally, other types of non-coding RNA, including microRNA and small nuclear RNA, were identified using INFERNAL^95^ to search the Rfam database.

### Gene family identification

We downloaded genome and annotation data of *Ananas comosus*^96^ (https://genomevolution.org/CoGe/NotebookView.pl?nid=937), *A. trichopoda*^97^ (http://amborella.huck.psu.edu; v.1.0), *A. officinalis*^98^ (https://genomevolution.org/coge/OrganismView.pl?dsgid=33908), *Arabidopsis thaliana*^99^ (TAIR 10), *Brachypodium distachyon*^100^ (purple false brome; Phytozome v.9.0), *Musa acuminata*^101^ (http://ensemblgenomes.org, release 21), *Oryza sativa*^102^ (Nipponbare, IRGSP-1.0), *Pho. dactylifera*^103^ (http://qatar-weill.cornell.edu/research/datepalmGenome), *P. equestris*^21^ (ftp://ftp.genomics.org.cn/from_BGISZ/20130120/), *Po. trichocarpa*^104^ (http://ensemblgenomes.org, release 21), *Sorghum bicolor*^105^ (sorghum; Phytozome v.9.0), *S. polyrhiza* (common duckweed; http://www.spirodelagenome.org), *Vitis vinifera*^106^ (Phytozome v.9.0), *D. catenatum*^22^ (http://www.ncbi.nlm.nih.gov/bioproject/262478), *A. shenzhenica*^23^ (https://www.ncbi.nlm.nih.gov/bioproject/310678), *D. chrysotoxum*^28^ (https://www.ncbi.nlm.nih.gov/bioproject/691441), *V. planifolia*^26^ (https://www.ncbi.nlm.nih.gov/bioproject/?term=PRJNA633886) and *Pha. aphrodite*^27^ (http://orchidstra2.abrc.sinica.edu.tw/orchidstra2/pagenome.php). The *G. elata* genome^24^ was downloaded from the NCBI (under project PVEL00000000) and re-annotated using the same pipeline as *P. zijinensis* and *P. guangdongensis* as described above (Supplementary Table 11). We selected the longest transcript to represent each gene and removed gene models with open reading frames shorter than 150 bp.

These protein sets were clustered into gene families using OrthoMCL v.2.0.9 (ref. ^107^) based on the sets of 24, 513 and 22,559 predicted genes of *P. zijinensis* and *P. guangdongensis*, respectively, and the protein-coding genes of 13 other monocots (*D. catenatum*, *Pha. aphrodite*, *Pha. equestris*, *A. shenzhenica*, *G. elata*, *A. comosus*, *A. officinalis*, *S. bicolor*, *B. distachyon*, *O. sativa*, *M. acuminata*, *S. polyrhiza* and *Pho. dactylifera*), three dicots (*Po. trichocarpa*, *A. thaliana* and *V. vinifera*) and the outgroup *A. trichopoda*. This analysis yielded 10,199 shared gene families in *P. zijinensis* and *P. guangdongensis* containing 15,795 and 13,793 predicted genes (64.43% and 61.14% of the total genes identified, respectively; orthologous genes in the 19 sequenced plant species are shown in Supplementary Fig. 19 and Supplementary Table 16). There were 234 single-copy gene families in the 19 species. A seven-way comparison of *A. shenzhenica*, *D. catenatum*, *Pha. aphrodite*, *Pha. equestris*, *G. elata*, *P. guangdongensis* and *P. zijinensis* in Orchidaceae (Supplementary Fig. 20) found 6,821 gene families to be shared by all taxa, with 710, 665, 915 and 363 gene families unique to *P. guangdongensis*, *P. zijinensis*, *D. catenatum* and *G. elata*, respectively.

After the identification of gene families, we selected gene families that existed in at least 16 of the 19 species and that had homologues in the early diverging angiosperm *A. trichopoda*. For these gene families that are probably conserved across angiosperms, we calculated the *F* index to compare the observed gene-family size in each species and the average gene-family size of each gene family using the following formula^30^:

$$F = \frac{{{{{\mathrm{log}}}}_2\;\left( {a\frac{{c_{ij}}}{{N_j}} + \frac{1}{2}} \right) + 1}}{{{{{\mathrm{log}}}}_2\;\left( {a + \frac{1}{2}} \right) + 1}}$$

where *c*_*ij*_ represents the number of genes in species *i* and gene family *j*, *N*_*j*_ represents the total number of gene families *j* and *a* is calculated using the number of species in a gene family (*S*):

$$a = \frac{{S^2 - 2S}}{2}$$

If a species has an average number of genes in a gene family, then the *F* index of the gene family for the species is equal to 0.5. Therefore, we classified the selected gene families in each species into five categories according to their *F* index: “Lost” with an *F* index equal to 0; “Less than average” with an *F* index greater than 0 but less than or equal to 0.45; “Around average (less)” with an *F* index greater than 0.45 but less than or equal to 0.5; “Around average (greater)” with an *F* index greater than 0.5 but less than or equal to 0.55; and “Greater than average” with an *F* index greater than 0.55.

### Phylogenomic dating

We constructed a phylogenetic tree based on a concatenated sequence alignment of 234 single-copy gene families from *P. guangdongensis* and *P. zijinensis* and 17 other plant species using MrBayes v.3.2.6 software with the maximum likelihood method. We then conducted phylogenomic dating in the Markov Chain Monte Carlo (MCMC) Tree program from PAML v4.6^108^. The MCMC process was run for 1,500,000 iterations with a sample frequency of 150 after a burn-in of 500,000 iterations. Other parameters used the default settings of MCMCTree. Two independent runs were performed to check the convergence. The following constraints were used for time calibrations:

1. *O. sativa* and *B. distachyon* divergence time: 40–54 Ma (ref. ^100^);
2. *Po. trichocarpa* and *A. thaliana* divergence time: 100–120 Ma (ref. ^104^);
3. monocot and eudicot divergence times with a lower boundary of 130 Ma (ref. ^106^);
4. 144–199 Ma as the upper boundary for the earliest-diverging angiosperms^109^;
5. Epidendroideae and Orchidoideae divergence time: 55–73 Ma (ref. ^109^);
6. *Dendrobium* and *Phalaenopsis* divergence time: 32–41 Ma (ref. ^109^); and
7. 14 Ma as the upper boundary for the divergence of *Platanthera*^109^.

### Gene family contractions and expansions

We used CAFE software (v.4.0)^40^ to identify the gene family contractions and expansions. First, we filtered the gene family statistics file containing the gene-family sizes for each species, and the family containing gene numbers larger than 100 in one species was filtered. The filtered table and the ultrametric tree of 19 plants were the input of CAFE; then, we set the parameters “load -p 0.05 -t 10 -r 1000 -filter” and the -t parameter in lambda command was set as different birth and death rates for different branches; Orchidaceae branches were set as 1, Poaceae branches set as 2, other monocot branches set as 3 and the other branches set as 4. For GO and KEGG enrichment analysis of contracted and expanded gene family, we used a one-sided hypergeometric test to perform the function enrichment and the Bonferroni method was used to make adjustments for multiple comparisons.

### Analyses of substitution rates

Identification of orthologues was performed first via reciprocal BLASTP with *E* (Expect) values <1 × 10^−5^ for proteins from the genomes of *D. catenatum*, *P. guangdongensis*, *P. zijinensis* and the OneKP transcriptome of *P. clavellata*^29^, followed by sorting BLAST hits by bit-scores and *E* values. The reciprocal best hits between *D. catenatum* and *P. guangdongensis*, *P. zijinensis* and *P. clavellata* were selected as orthologues. In this way, we identified 10,871 orthologues between *D. catenatum* and *P. guangdongensis*, 11,709 orthologues between *D. catenatum* and *P. zijinensis*, 10,235 orthologues between *D. catenatum* and *P. clavellata*, 10,202 orthologues between *P. clavellata* and *P. guangdongensis*, 10,908 orthologues between *P. clavellata* and *P. zijinensis* and 12,737 orthologues between *P. guangdongensis* and *P. zijinensis*. For each pair of orthologues, ClustalW^110^ alignment was carried out for sequence alignment using the parameter for amino acids recommended by Hall^111^. PAL2NAL^112^ was then used to back-translate aligned protein sequences into codon sequences and to remove any gaps in the alignment. Estimates of *K*_S_ values were obtained from CODEML in PAML v.4.9j using the Goldman–Yang model with codon frequencies estimated using the F3 × 4 model^109,110^. We also used the same approach to calculate one-to-one orthologous *K*_S_ distributions between *A. shenzhenica* and *G. elata*, *P. guangdongensis*, *P. zijinensis*, *P. clavellata*, *Pha. equestris*, *Pha. aphrodite* and *D. catenatum*. The *K*_S_ distance between any two species in the calculation was estimated by the mode inferred by resampling the corresponding orthologous *K*_S_ distribution 200 times (Fig. 4b and Extended Data Fig. 1).

To quantify the difference in substitution rates, we calculated the *K*_S_ distances of *P. guangdongensis*, *P. zijinensis* and *P. clavellata* after they diverged from their most recent common ancestor using the approach described in the relative rate test using *D. catenatum* as an outgroup^113^. For example, using the *K*_S_ distances between *D. catenatum* and *P. guangdongensis* and *P. clavellata* and the *K*_S_ distance between *P. clavellata* and *P. guangdongensis*, we calculated the *K*_S_ distances to the lineages of *P. guangdongensis* and *P. clavellata* after their divergence. Similarly, we calculated the *K*_S_ distances of *P. clavellata* and *P. zijinensis* after their divergence. The summarized results are shown in Fig. 4a.

### Whole-genome duplication

*K*_S_-based age distributions for all the paralogues of *P. zijinensis* and *P. guangdongensis* were constructed as previously described^114^. In addition, paralogous gene pairs located in duplicated segments (anchors) were identified in the chromosome-level assembled genomes of *P. zijinensis* and *P. guangdongensis* using i-ADHoRe (v.3.0)^115,116^. The resulting *K*_S_ distributions of *P. guangdongensis* and *P. zijinensis* are shown in Supplementary Figs. 7 and 8, respectively.

### Analysis of genes related to leaf development and nitrogen and phosphorus acquisition

The candidate genes related to leaf development and nitrogen and phosphorus acquisition were collected from sequencing data of *Pha. equestris*, *D. catenatum*, *P. zijinensis*, *P. guangdongensis* and *G. elata* based on BLAST searches using *Arabidopsis* genes reported in several review articles as queries^32,48,49,117–120^. Once a candidate gene was identified, the sequence was further subjected to a reverse-BLAST search against the *Arabidopsis* genome. When the original *Arabidopsis* query was the top hit, the candidate gene was defined as the orthologue of the *Arabidopsis* query. The amino acids of the candidate sequences were aligned using ClustalW v.2.1 (http://clustalw.ddbj.nig.ac.jp/), and phylogenetic trees were constructed using MEGA6 v.6.06 (http://www.megasoftware.net/) using the bootstrap neighbour-joining method.

### KEGG annotation of the photosynthetic pathway in six orchid species

We used DIAMOND to compare the protein set of species with the KEGG library and set the parameters as: max-target-seqs, 1; evalue, 0.00001; id, 30; query-cover, 50 and subject-cover, 50, which requires query and subject to reach coverage equal to 50% as a credible comparison.

### Identification and phylogenetic analysis of MADS-box genes

MADS-box genes were identified by searching InterProScan^92^ for the results of all the predicted *P. zijinensis*, *P. guangdongensis* and *G. elata* proteins. The predicted genes were manually inspected for which gene predictions were short or the MADS- or K-domains were only partially included. MADS-box domains comprising 60 amino acids, identified via SMART^121^ for all the MADS-box genes, were then aligned using ClustalW. An unrooted maximum likelihood tree was constructed in MEGA5 (ref. ^122^) with default parameters. Bootstrap analysis was performed using 1,000 iterations.

### Gene expression analysis

#### Transcriptomes

Transcriptome sequencing was entrusted by Beijing Genomics Institute and paired-end-sequenced with an Illumina HiSeq™ 2000 system of Gene Denovo Biotechnology Co., Ltd.

RNA-Seq was performed on 21 samples, 12 from *P. zijinensis* and 9 from *P. guangdongensis*. The quality control of the reads was performed with FASTQC whereas trimming and clipping were performed with BBDuk (v.35.85; https://jgi.doe.gov/data-and-tools/bbtools/). The minimum length of the reads after trimming was set to 35 bp and the minimum base quality score (Phred) to 25. Mapping was performed against *P. zijinensis* and *P. guangdongensis* reference genomes with STAR aligner (v.2.5.0c; https://github.com/alexdobin/STAR) with the following options: –outSAMtype BAM SortedByCoordinate–alignIntronMax 14000–alignEndsType EndToEnd–alignEndsProtrude 20 ConcordantPair–chimOutType WithinBAM–chimSegmentMin 50–twopassMode Basic. FeatureCounts (v.1.6; http://bioinf.wehi.edu.au/featureCounts/) was used to obtain gene expression values as raw read counts across all the samples. Finally, the gene expression values were normalized as fragments per kilobase per million mapped reads using edgeR (http://bioconductor.org/packages/release/bioc/html/edgeR.html). Heatmaps of the expression profiles were produced with gplots (https://cran.r-project.org/web/packages/gplots/index.html). We have three replicates for each tissue, and the mean and standard deviation of expression level of concerned genes were calculated.

#### Real-time PCR

*C. goeringii* was collected from Yunnan Province, China. Total RNA was extracted using a Plant RNA MiniPrep™ kit (Zymo Research Corporation). Using 500 ng of RNA, complementary DNA was synthesized via reverse transcription based on a HiScript II Q Select RT SuperMix for qPCR (+gDNA wiper) kit (Vazyme Biotech Co., Ltd) and a (dT)15 primer. cDNA (1 μl) was used for subsequent qRT-PCR using the AceQ qPCR SYBR® Green Master Mix kit (Vazyme Biotech Co., Ltd) in an ABI StepOne system (Applied Biosystems) according to the default protocol. Each sample was analysed in triplicate. The primers used in this study are listed in Supplementary Table 39.

### Reporting Summary

Further information on research design is available in the Nature Research Reporting Summary linked to this article.

## Supplementary information

Supplementary Information Supplementary Notes 1–3, Figs. 1–21 and Tables 1–7, 9–24, 31–36, 38 and 39.

Reporting Summary

Supplementary Data 1 Supplementary Tables 8, 25–30, 37 and 40–46.

## Data Availability

Genome sequences and whole-genome assemblies have been submitted to the NCBI database under BioProject PRJNA739531.
