# Supplementary material for: Genomes of leafy and leafless Platanthera orchids illuminate the evolution of mycoheterotrophy
Source: Nat Plants. 2022 Apr 21;8(4):373–88. doi: 10.1038/s41477-022-01127-9 (PMC9023349; doi:10.1038/s41477-022-01127-9)
Supplement: Supplementary file 1 — Supplementary Notes 1–3, Figs. 1–21 and Tables 1–7, 9–24, 31–36, 38 and 39. [file 41477_2022_1127_MOESM1_ESM.pdf]

nature portfolio

https://doi.org/10.1038/s41477-022-01127-9

Supplementary information

Genomes of leafy and leafless *Platanthera* orchids illuminate the evolution of mycoheterotrophy

In the format provided by the authors and unedited

Supplementary Information

Genomes of leafy and leafless *Platanthera* orchids illuminate the evolution of mycoheterotrophy

Ming-He Li[1, 2*], Ke-Wei Liu[3*], Zhen Li[4, 5*], Hsiang-Chia Lu[1, 6*], Qin-Liang Ye[7*], Diyang Zhang[1, 2], Jie-Yu Wang[1, 8], Yu-Feng Li[7], Zhi-Ming Zhong[7], Xuedie Liu[1, 2], Xia Yu[1, 2], Ding-Kun Liu[1, 2], Xiong-De Tu[1, 2], Bin Liu[1, 2], Yang Hao[1, 2], Xing-Yu Liao[1, 2], Yu-Ting Jiang[1, 2], Wei-Hong Sun[1, 2], Jinliao Chen[1,2], Yan-Qiong Chen[1, 2], Ye Ai[1,2], Jun-Wen Zhai[1, 2], Sha-Sha Wu[1, 2], Zhuang Zhou[1, 2], Yu-Yun Hsiao[9], Wan-Lin Wu[9,10], You-Yi Chen[9, 10], Yu-Fu Lin[10], Jui-Ling Hsu[10], Chia-Ying Li[11], Zhi-Wen Wang[12], Xiang Zhao[12], Wen-Ying Zhong[12], Xiao-Kai Ma[1], Liang Ma[1], Jie Huang[1], Gui-Zhen Chen[1], Ming-Zhong Huang[1], Laiqiang Huang[3], Dong-Hui Peng[1, 2], Yi-Bo Luo[13], Shuang-Quan Zou[1, 2], Shi-Pin Chen[1], Siren Lan[1, 2†], Wen-Chieh Tsai[9,10†], Yves Van de Peer[4, 5, 14, 15†], Zhong-Jian Liu[1, 2, 16†]

Contents

Supplementary Notes **1**

Supplementary Note 1. Evolution of chloroplast and mitochondrial genomes 1

Supplementary Note 2. Genes involved in leaf initialization and leaf development 3

Supplementary Note 3. Selective pressure of homologous genes involved in the photosynthesis pathway 4

Supplementary Figures **6**

Supplementary Figure 1. *K*-mer distribution of sequencing reads of *P. zijinensis* with 1.80% heterozygosity. 6

Supplementary Figure 2. *K*-mer distribution of sequencing reads of *P. guangdongensis* with 1.89% heterozygosity. 7

Supplementary Figure 3. The Hi-C interaction matrices for chromosomes. 8

Supplementary Figure 4. The LTR insertion time distributions of *P. zijinensis* and *P. guangdongensis*. 9

Supplementary Figure 5. Common lost BUSCOs of *P. zijinensis*, *P. guangdongensis* and *G. elata*. 10

Supplementary Figure 6. Syntenic block of *V. planifolia*, *P. zijinensis*, *P. guangdongensis* and *D. chrysotoxum*. 15

Supplementary Figure 7. K_S distributions for the whole paranome (up) and the anchor pairs (bottom) in the *P. zijinensis* genome. 16

Supplementary Figure 8. K_S distributions for the whole paranome (up) and the anchor pairs (bottom) in the *P. guangdongensis* genome. 17

Supplementary Figure 9. Maximum likelihood phylogram of 19 angiosperms inferred based on single-copy genes. 18

Supplementary Figure 10. Venn diagram indicates the common lost genes of *G. elata* and *P. guangdongensis*. 19

Supplementary Figure 11. Chloroplast genome of *P. zijinensis*. 20

Supplementary Figure 12. Chloroplast genome of *P. guangdongensis*. 21

Supplementary Figure 13. Chloroplast genome collinearity mapping of *D. catenatum*, *P. guangdongensis*, and *P. zijinensis*. 22

Supplementary Figure 14. Mitochondrial genome of *P. zijinensis* and *P. guangdongensis*. 23

Supplementary Figure 15. Phylogenetic analysis of type II MADS-box genes in orchids. 24

Supplementary Figure 16. Expression patterns of trehalase genes in various organs of autotrophic orchid *D. catenatum*, fully mycoheterotrophic orchid *P. guangdongensis* (PG), and partially mycoheterotrophic orchid *P. zijinensis* (PZ). 25

Supplementary Figure 17. Expression patterns of SUT genes in various organs of *P. guangdongensis*. 26

Supplementary Figure 18. The boxplot of gene elements length for ten plant species. 27

Supplementary Figure 19. Ortholog groups found in various plant species. 28

Supplementary Figure 20. A seven-way comparison of *A. shenzhenica* (ASHE), *D. catenatum* (DCAT), *G. elata* (GELA), *Pha. aphrodite* (PAPH), *Pha. equestris* (PEQU), *P. guangdongensis* (PGUA) and *P. zijinensis* (PZIJ) in Orchidaceae. 29

Supplementary Figure 21. Expression pattern of transcriptome analysis for leaf development-related genes. 30

Supplementary Tables **31**
Supplementary Table 1. The statistics of *P. zijinensis* sequencing raw data from Pacific Biosciences platforms. 31
Supplementary Table 2. The statistics of *P. guangdongensis* sequencing raw data from Pacific Biosciences platforms. 32
Supplementary Table 3. Summary of the *P. zijinensis* and *P. guangdongensis* genomes' assembly. 33
Supplementary Table 4. BUSCO assessment of genome assembly for *P. zijinensis* and *P. guangdongensis*. 34
Supplementary Table 5. Chromosome length (bp) of *P. zijinensis* and *P. guangdongensis*. 35
Supplementary Table 6. Summary of the *P. zijinensis* and *P. guangdongensis* genomes Hi-C assembly. 36
Supplementary Table 7. Categories of TEs predicted in *P. zijinensis*, *P. guangdongensis* and *G. elata* genomes 37
Supplementary Table 8. The subtypes of repeats for six orchid species (see separate files). 38
Supplementary Table 9. LTR insertion distribution of *P. zijinensis*. 39
Supplementary Table 10. LTR insertion distribution of *P. guangdongensis*. 40
Supplementary Table 11. The prediction of gene structures of *P. zijinensis*, *P. guangdongensis* and *G. elata*. 41
Supplementary Table 12. Gene function annotation of *P. zijinensis* and *P. guangdongensis*. 42
Supplementary Table 13. Summary of ncRNA annotation of *P. zijinensis* and *P. guangdongensis*. 43
Supplementary Table 14. BUSCO assessment of genome annotation in six orchids. 44
Supplementary Table 15. Statistics of syntenic analysis in *P. guangdongensis*, *P. zijinensis*, *Pha. aphrodite, V. planifolia* and *D. chrysotoxum*. 45
Supplementary Table 16. Summary of orthologous gene families in 19 sequenced plant species. 46
Supplementary Table 17. GO enrichment analysis for missing gene families in *S. polyrhiza*. 47
Supplementary Table 18. GO enrichment analysis for missing gene families in *Pho. dactylifera*. 48
Supplementary Table 19. GO enrichment analysis for missing gene families in *P. guangdongensis*. 49
Supplementary Table 20. GO enrichment analysis for missing gene families in *G. elata*. 50
Supplementary Table 21. Photosynthesis-related KEGG pathway and genes in six orchids.......... 51
Supplementary Table 22. Chloroplast genes of *P. zijinensis* and *P. guangdongensis*. 52
Supplementary Table 23. GO enrichment analysis for missing gene families in *P. zijinensis*........ 54
Supplementary Table 24. GO enrichment analysis for missing gene families in *A. officinalis*. 55
Supplementary Table 25. Significantly contracted gene families on the branch leading to the divergence between *P. zijinensis* and *P. guangdongensis* (see separate file). 56
Supplementary Table 26. Significantly expanded gene families on the branch leading to the divergence between *P. zijinensis* and *P. guangdongensis* (see separate file). 56
Supplementary Table 27. Significantly contracted gene families on the branch leading to *P. zijinensis* (see separate file). 56
Supplementary Table 28. Significantly expanded gene families on the branch leading to *P. zijinensis* (see separate file). 56
Supplementary Table 29. Significantly contracted gene families on the branch leading to *P.*

guangdongensis (see separate file). 56
Supplementary Table 30. Significantly expanded gene families on the branch leading to *P. guangdongensis* (see separate file). 56
Supplementary Table 31. Number of genes related to leaf initiation and development in the genomes of *A. thaliana* and orchids*. 57
Supplementary Table 32. Transcription factor genes involved in root development. 58
Supplementary Table 33. List of 46 MADS-box genes identified in *P. zijinensis* 59
Supplementary Table 34. List of 47 MADS-box genes identified in *P. guangdongensis* 61
Supplementary Table 35. Trehalase genes in sequenced plant genomes 63
Supplementary Table 36. Number of genes related to nutrient transport and assimilation in the genomes of *A. thaliana* and orchids. 64
Supplementary Table 37. Sequences of trehalase genes in *C. goeringii* (see separate file). 65
Supplementary Table 38. Length distribution of gene elements in sequenced plants. 66
Supplementary Table 39. Primers used in this study. 67
Supplementary Table 40. GO enrichment terms of significantly expanded gene families of *P. guangdongensis* (see separate file). 68
Supplementary Table 41. Mitochondrial genes of *P. zijinensis* and *P. guangdongensis*. 69
Supplementary Table 42. W value of other genes except for photosynthetic homologs of *P. guangdongensis* and *P. zijinensis* (see separate file). 71
Supplementary Table 43. W value of photosynthetic homologs of *P. guangdongensis* and *P. zijinensis* (see separate file). 71
Supplementary Table 44. W value of each branch after the divergence of *P. guangdongensis* and *P. zijinensis* using *A. shenzhenica* as outgroup (see separate file). 71
Supplementary Table 45. Gene element length for ten plant species (see separate file). 71
Supplementary Table 46. Gene IDs for leaf initiation and development (see separate file). 71
Supplementary References 72

Supplementary Notes

Supplementary Note 1. Evolution of chloroplast and mitochondrial genomes

Degeneration of the photosynthesis system

As *P. guangdongensis* does not perform photosynthesis, it was unsurprising that genes enriched for 'chloroplast' annotations were strongly represented among the missing genes (**Supplementary Tables 22, 40**). To further investigate the putative functions of missing genes, we examined genes related to the photosynthetic apparatus, namely Photosystem I, Photosystem II, Cytochrome b_6f, Cytochrome C_6, ATP synthase, and Rubisco. Of the nine nuclear genes coding for photosynthetic apparatus proteins in *P. zijinensis*, only six and two were present in the *P. guangdongensis* and *G. elata* genomes, respectively; this was significantly fewer than in *P. zijinensis* and other sequence orchids (**Supplementary Table 22**). We assumed that these genes were nonfunctional because their full complements of subunits were not present.

We assembled the chloroplast genomes of *P. guangdongensis* and *P. zijinensis* (MZ440660 and MZ440661). We found that the chloroplast genome of *P. guangdongensis* (88,060 bp) was dramatically restructured and reduced in size, similar to the reduction in gene number observed for the nuclear genome, compared to the chloroplast genomes of *P. zijinensis* (151,858 bp) (**Supplementary Figures 11, 12**). The chloroplast genomes of these two species comprise two single-copy regions (a large and a small single-copy region) and the two identical large inverted repeats (IRs) encode 128 and 60 genes, respectively, of which *P. zijinensis* is most associated with photosynthesis. The *P. guangdongensis* chloroplast genome has lost one IR and encodes only 28 protein-coding genes (**Supplementary Figure 12**), suggesting that *P. guangdongensis* is an ancient full mycoheterotrophy and that its chloroplast genome is in the last stage of a 'degradation ratchet', that is, retention and loss of the five core nonbioenergetic genes[1,2]. These results clearly show that both the chloroplast and nuclear genomes of *P. guangdongensis* have lost most of the genes required for photosynthesis, although the highly degraded chloroplast genome is still essential for this full mycoheterotrophy (**Supplementary Figures 11–13**).

Contraction of the mitochondrial genome

Although the *P. guangdongensis* genome has undergone extensive gene loss, we found that 550 gene families containing 1,431 genes (25 by a significant margin) showed expansion in *P. guangdongensis* compared to *P. zijinensis* (**Figure 2; Supplementary Table 30**). These genes were enriched for GO terms related to several metabolic processes (**Supplementary Table 40**). We speculate that these expanded genes are related to the functional requirements of the obligate mycoheterotrophic lifestyle of *P. guangdongensis.* We assembled the mitochondrial genome to explore this idea, and the mitochondrial genome of *P. guangdongensis* was contracted in size (1,126,452 bp, **Supplementary Figure 14b**) compared to the mitochondrial genomes of *P. zijinensis* (1,215,055 bp, **Supplementary Figure 14a**). Thirty-three and 37 protein-coding genes, 19 and 20 tRNA genes, and one rRNA gene were annotated in *P. guangdongensis* and *P. zijinensis*, respectively, showing gene contraction with one gene family (*Mat-R*, one gene) and four genes (Atp family, *Atp6*; Rp family, *Rp110*, *Rps1*, and *Rps2*) in *P. guangdongensis* compared to *P. zijinensis* (**Supplementary Table 41**). For example, the ATP synthase gene family in *P. zijinensis* has five genes, whereas *P. guangdongensis* has four genes and loses *Atp6*. However, the rRNA gene family has one *Rps7* gene in *P. guangdongensis*; it is lost in *P. zijinensis*. *Rps7* is one of the primary rRNA-binding protein genes; its molecular functions include mRNA and rRNA binding, and it is also a structural constituent of ribosomes[3]. This gene is located upstream of one 26S rRNA gene in the mitochondrial genome and is expressed as an abundant mRNA[4].

In addition, we analysed and compared the chloroplast and mitochondrial genomes. It is a *P. guangdongensis* nuclear gene that is integrated into the mitochondrial genome, which might be related to the energy supply of *P. guangdongensis*. The lost chloroplast genes may be transferred to the nuclear and mitochondrial genomes[5]. The lost chloroplast genes of *P. guangdongensis* were queried in the nuclear and mitochondrial genomes, which only found *psbE* located in the *P. guangdongensis* (*PGU012205*) and *P. zijinensis* (*PZI018842*) nuclear genomes, indicating that the lost genes were not transferred to the nucleus and mitochondria. Interestingly, *P. guangdongensis* retains more genes in the photosynthesis pathways than does *G. elata*, showing a closer number to the ones in *P. zijinensis*, which predicts that more *P. guangdongensis* genes involved in

photosynthesis are lost. Selective pressure analysis (see **Supplementary Note 3**) showed that among all the orthologous pairs of collinear blocks in *P. guangdongensis* and *P. zijinensis*, 24 orthologous pairs involved in photosynthesis showed lower w values (*Ka*/*Ks*) (T_{test} P value=0.07258) (**Supplementary Tables 42, 43**). Using *A. shenzhenica* as an outgroup, the w value of each clade showed that *P. zijinensis* was higher than *P. guangdongensis* (T_{test} P value =0.0882) (**Supplementary Table 44**). Considering the close phylogenetic relationship between *P. zijinensis* and *P. guangdongensis* and their distinguished habitats in the same region, we deduce parsimoniously that the most recent common ancestor (MRCA) of *P. zijinensis* and *P. guangdongensis* was a mixotrophy similar to *P. zijinensis* and that the invasion of MRCA into the local shaded forest eventually led to the appearance of fully mycoheterotrophic *P. guangdongensis*.

Supplementary Note 2. Genes involved in leaf initialization and leaf development

We are interested in determining whether specific leaf initiation genes have been lost in *P. guangdongensis* and *G. elata* in relation to the loss of photoreceptor genes. To this end, we used *Arabidopsis* genes that are well known for regulating leaf initiation and development, including auxin synthetic/responsive genes and transcription factors, as queries to analyse the genomes of *P. guangdongensis*, *P. zijinensis*, *G. elata*, *D. catenatum*, and *Pha. equestris*[6-9] (**Extended Data Figures 6, 7; Supplementary Table 31**).

Among the key genes for leaf initiation, *P. guangdongensis* loses PLETHORA (PLT) transcription factors while *P. zijinensis* has two copies, which are required for the maintenance of high-level expression of *PIN1* during leaf development[10]. Similarly, *G. elata* has one homologue to PLT transcription factors (**Supplementary Table 31**). *PIN1* is an auxin efflux transporter gene. It can be expressed in the shoot apical meristem (SAM) and redistributes auxin generated at the meristem dome to the incipient primordia, so the expression of *PIN1* is important for auxin to trigger organogenesis at the SAM[10,11]. The numbers of *PIN1* genes were comparable in the six orchid genomes; *P. guangdongensis* and *G. elata* have only one homologue compared to *P. zijinensis* (3) (**Supplementary Table 31**), likely because they are involved in multiple developmental processes[12]. Although the genomes of *P. guangdongensis* and *P. zijinensis* still

have one homologue of the auxin synthetic pathway gene (YUC), whereas *G. elata* has lost it, the contraction (loss) of upstream *PLT* transcription factors may affect the expression of *PIN1* and affect the distribution of auxin at the SAM in *P. guangdongensis* and *G. elata*. Furthermore, as dark treatment can affect the subcellular localization of *PIN1* and cease leaf initiation in tomatoes[13], the loss of photoreceptor genes may also affect the subcellular localization of *PIN1* in *P. guangdongensis* and *G. elata*, eventually leading to the leafless phenotype. Nevertheless, genes related to leaf localization and development are not entirely lost in *P. guangdongensis* and *G. elata*, which still contain homologues of *ASYMMETRIC LEAVES1 (AS1)*, another key regulator of leaf initiation in *Arabidopsis*[7].

For leaf development genes, the homologue numbers of *Arabidopsis* transcription factor families, including *Class II TCP* and *CUC*, were reduced in the genomes of *P. guangdongensis* and *G. elata* compared with other photosynthetic orchids (**Supplementary Table 31**). Our phylogenetic analysis of *TCP* genes showed that *P. guangdongensis* and *G. elata* had only four and three genes of the *Class II CIN-TCP* family, respectively, which contained six and eight genes in *P. zijinensis* and *A. thaliana*, respectively (**Extended Data Figure 7**). Interestingly, other *TCP* genes related to leaf development in *Arabidopsis* such as *TCP 4/5/10/13/17/23* do not exist in the genomes of *P. guangdongensis*. *Class II CIN-TCP* genes can regulate leaf development in *Arabidopsis*[7]. One of these two *Class II CIN-TCP* genes in *P. guangdongensis* was not expressed in any of the analysed organs (**Supplementary Figure 21; Supplementary Table 31**), suggesting that the leafless feature of *P. guangdongensis* is also correlated with the loss of *Class II TCP* genes.

Supplementary Note 3. Selective pressure of homologous genes involved in the photosynthesis pathway

To investigate the selection pressure of homologous genes in the photosynthesis pathway shared by *P. guangdongensis* and *P. zijinensis*, 24 pairs of homologous genes in photosynthesis were found to be shared by *P. guangdongensis* and *P. zijinensis*. First, *Ka*/*Ks* (w value) was calculated for all homologous pairs in the collinear block of *P. guangdongensis* and *P. zijinensis*; then, it was determined whether the w values of these 24 homologous pairs and other gene pairs were

significantly different. Second, we used BLASTP alignment to identify the homologous genes of 24 pairs of genes in *A. shenzhenica*, and then performed multiple sequence alignment for each group of homologous genes. According to the tree structure of (*A. shenzhenica*, (*P. guangdongensis*, *P. zijinensis*)), codeml was used to set the runmode = 0, model = 1, and NSsites = 0. The *Ka*, *Ks* and w values of each branch were calculated. Taking *A. shenzhenica* as the outgroup, w value of each branch was calculated after the differentiation of each group of genes *P. guangdongensis* and *P. zijinensis*.

Supplementary Figures

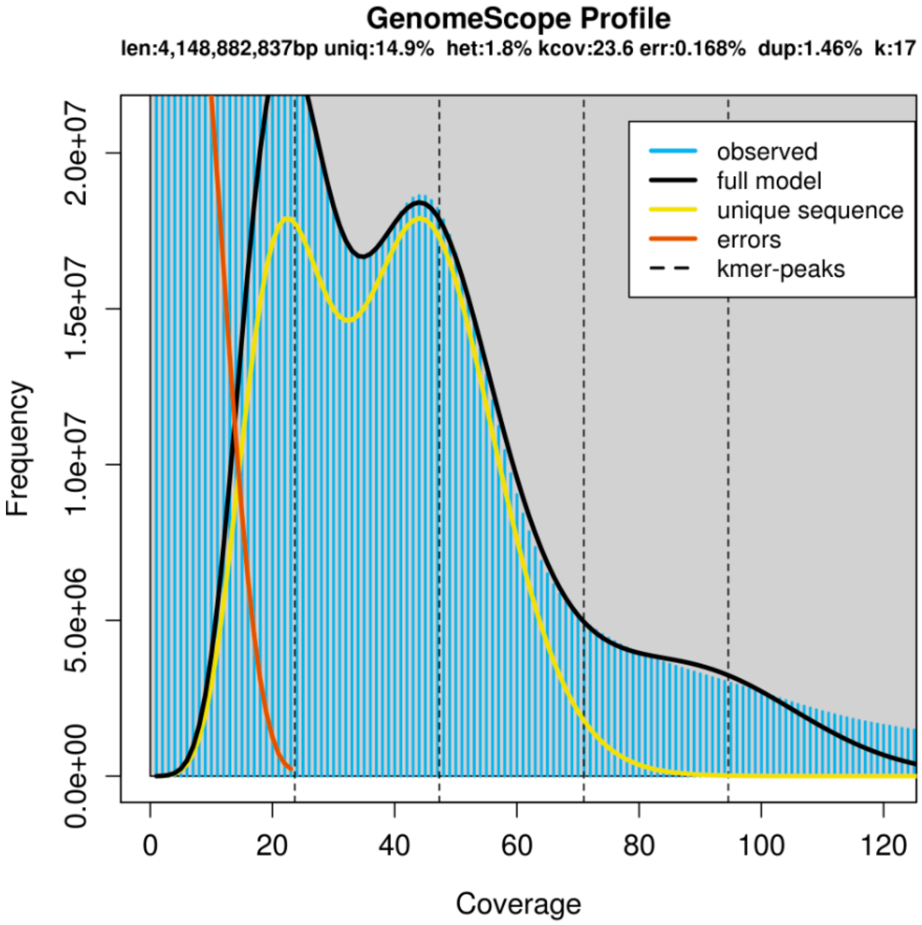

Supplementary Figure 1. *K*-mer distribution of sequencing reads of *P. zijinensis* with 1.80% heterozygosity.

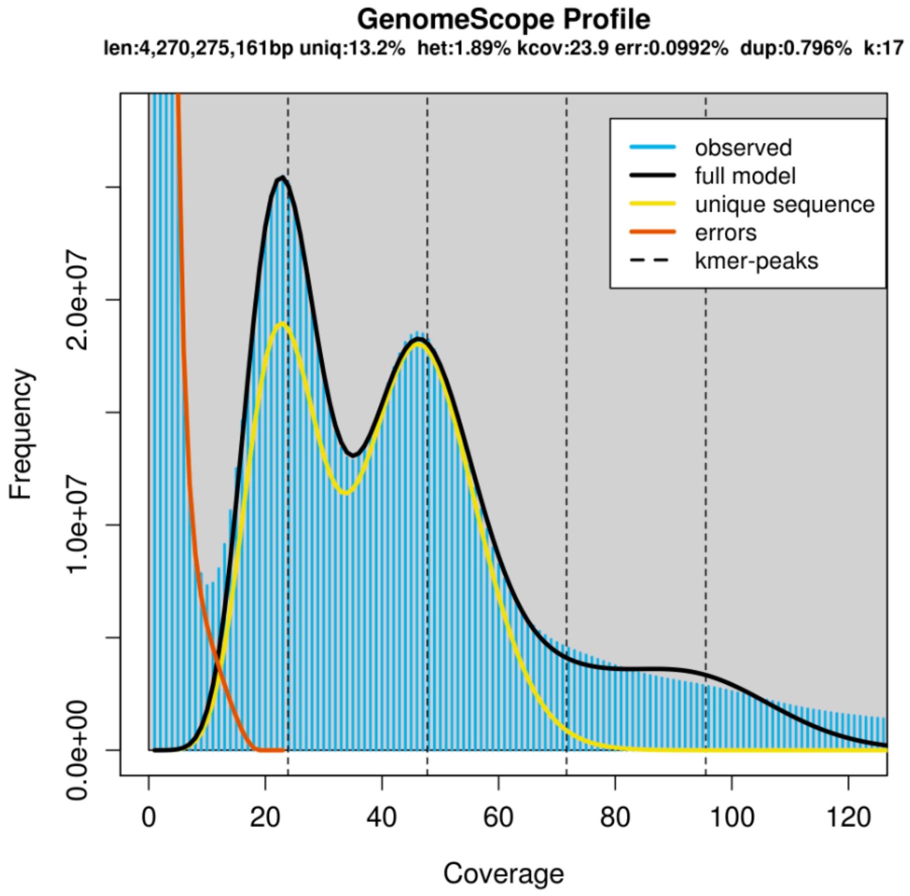

Supplementary Figure 2. *K*-mer distribution of sequencing reads of *P. guangdongensis* with 1.89% heterozygosity.

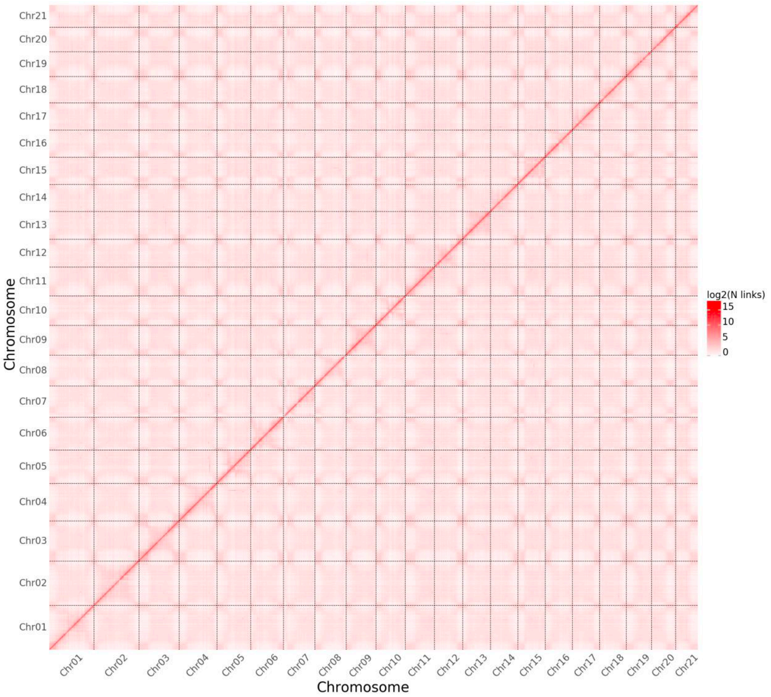

a

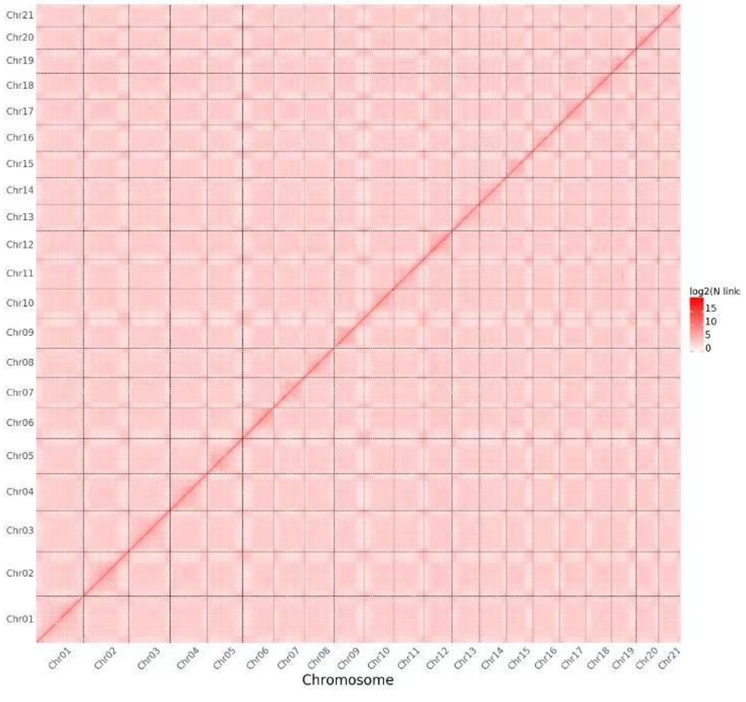

b

Supplementary Figure 3. The Hi-C interaction matrices for chromosomes. a. *P. zijinensis*. **b**. *P. guangdongensis*.

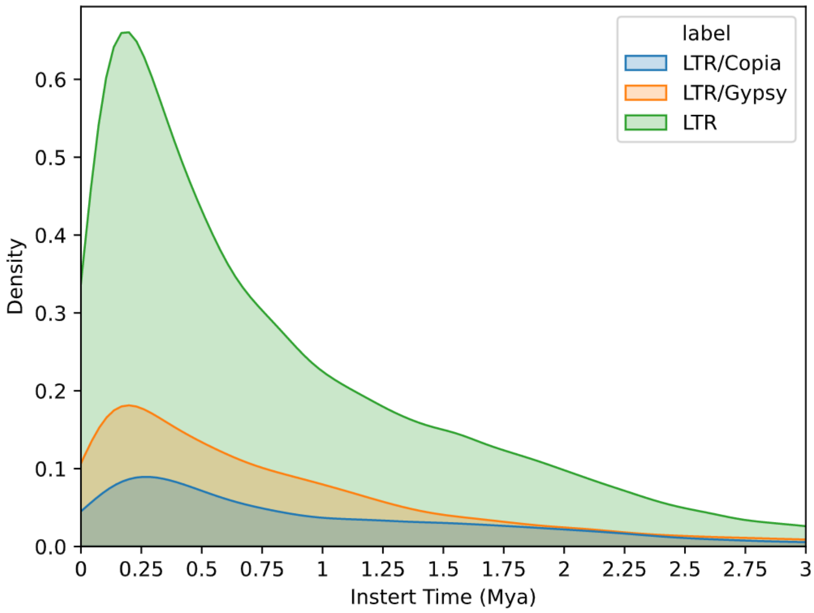

a

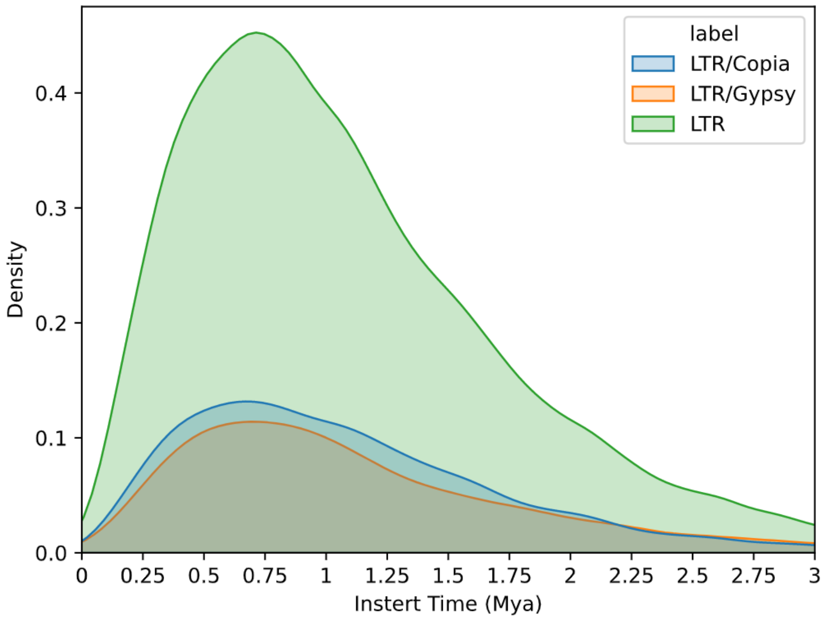

b

Supplementary Figure 4. The LTR insertion time distributions of *P. zijinensis* and *P. guangdongensis*. Red, green, and blue distributions represent the insertion time of total LTR, *Copia* and *Gypsy*, respectively. **a**. The insertion time of total LTR, *Copia* and *Gypsy* of *P. zijinensis*. **b**. The insertion time of total LTR, *Copia* and *Gypsy* in *P. guangdongensis*.

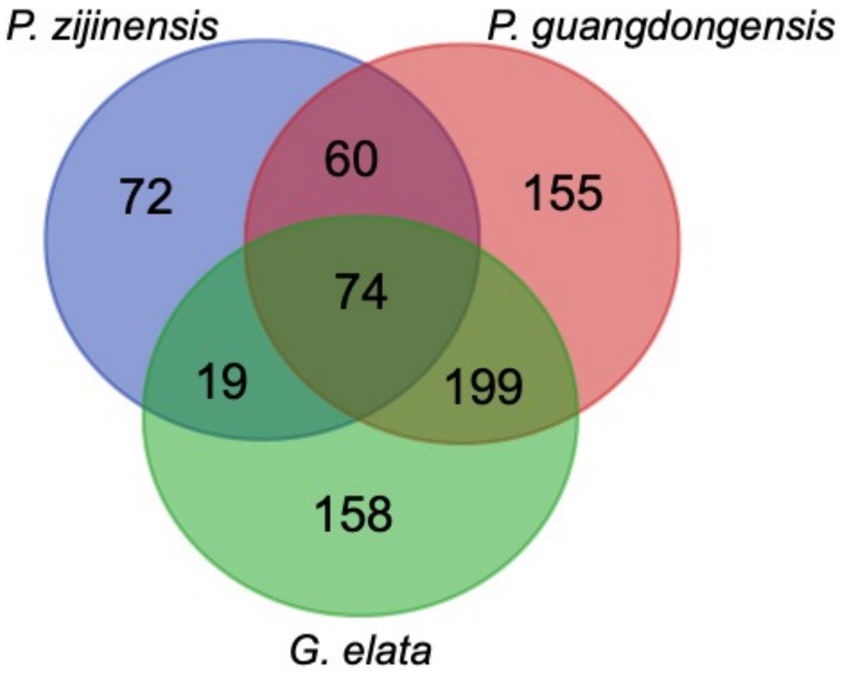

Supplementary Figure 5. Common lost BUSCOs of *P. zijinensis*, *P. guangdongensis* and *G. elata*.

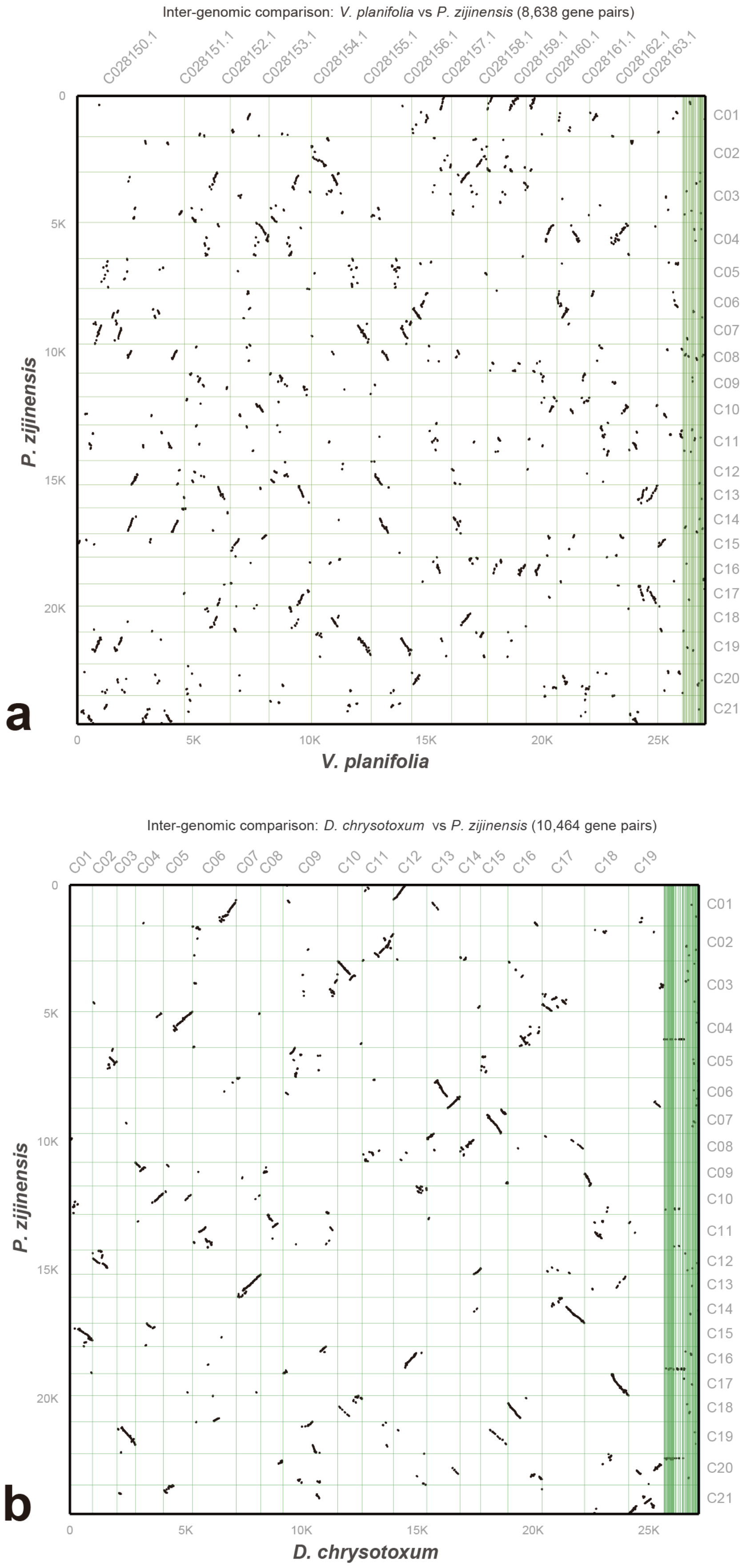
Inter-genomic comparison: V. planifolia vs P. zijinensis (8,638 gene pairs)
C028150.1
C028151.1
C028152.1
C028153.1
C028154.1
C028155.1
C028156.1
C028157.1
C028158.1
C028159.1
C028160.1
C028161.1
C028162.1
C028163.1
C01
C02
C03
C04
C05
C06
C07
C08
C09
C10
C11
C12
C13
C14
C15
C16
C17
C18
C19
C20
C21
0
5K
10K
15K
20K
25K
P. zijinensis
V. planifolia
a
Inter-genomic comparison: D. chrysotoxum vs P. zijinensis (10,464 gene pairs)
C01
C02
C03
C04
C05
C06
C07
C08
C09
C10
C11
C12
C13
C14
C15
C16
C17
C18
C19
C20
C21
0
5K
10K
15K
20K
25K
P. zijinensis
D. chrysotoxum
b

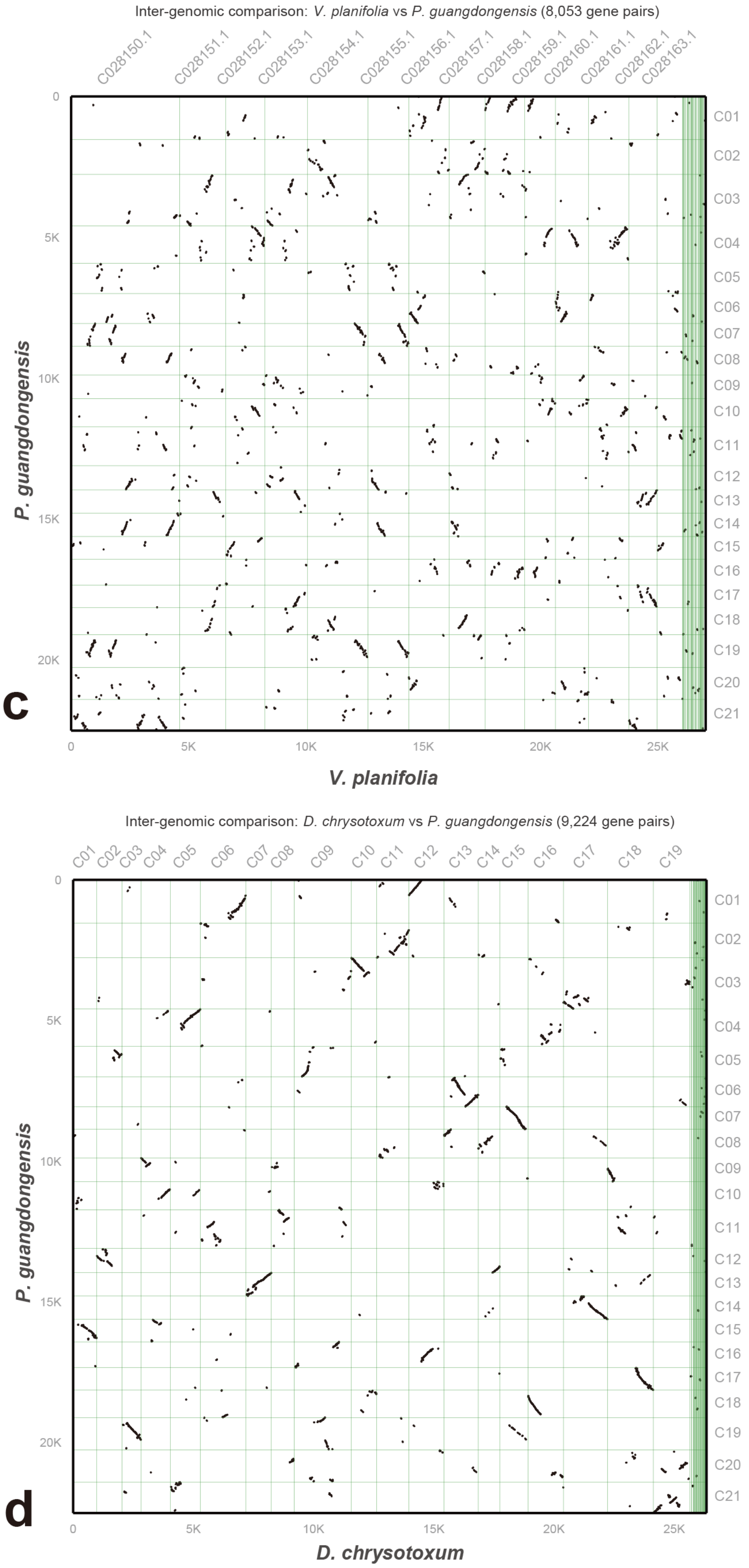
Inter-genomic comparison: V. planifolia vs P. guangdongensis (8,053 gene pairs)
C028150.1
C028151.1
C028152.1
C028153.1
C028154.1
C028155.1
C028156.1
C028157.1
C028158.1
C028159.1
C028160.1
C028161.1
C028162.1
C028163.1
C01
C02
C03
C04
C05
C06
C07
C08
C09
C10
C11
C12
C13
C14
C15
C16
C17
C18
C19
C20
C21
0
5K
10K
15K
20K
25K
P. guangdongensis
V. planifolia
c
Inter-genomic comparison: D. chrysotoxum vs P. guangdongensis (9,224 gene pairs)
C01
C02
C03
C04
C05
C06
C07
C08
C09
C10
C11
C12
C13
C14
C15
C16
C17
C18
C19
C20
C21
P. guangdongensis
D. chrysotoxum
d

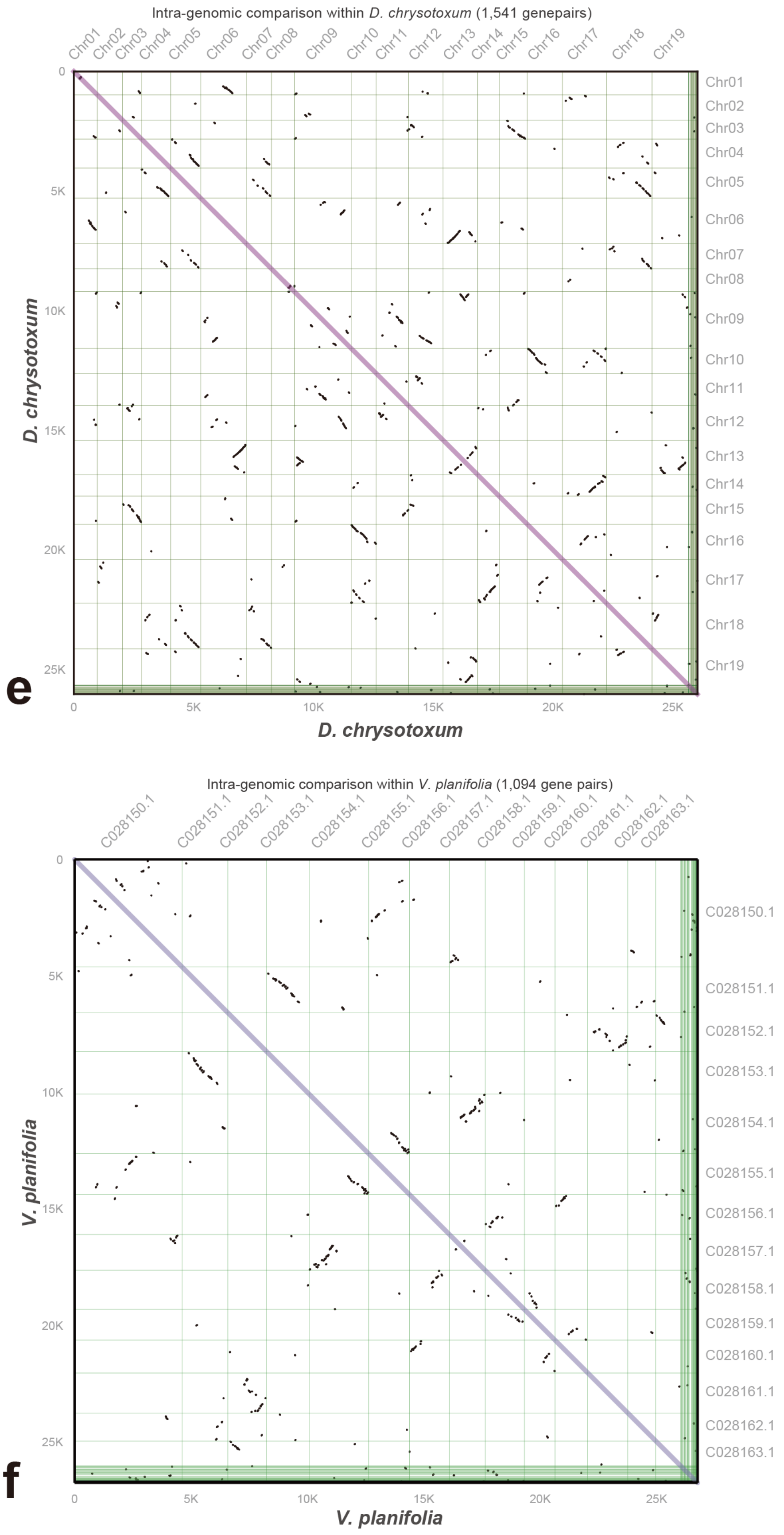
Intra-genomic comparison within D. chrysotoxum (1,541 genepairs)
Chr01 Chr02 Chr03 Chr04 Chr05 Chr06 Chr07 Chr08 Chr09 Chr10 Chr11 Chr12 Chr13 Chr14 Chr15 Chr16 Chr17 Chr18 Chr19
0
5K
10K
15K
20K
25K
D. chrysotoxum
D. chrysotoxum
e
Intra-genomic comparison within V. planifolia (1,094 gene pairs)
C028150.1 C028151.1 C028152.1 C028153.1 C028154.1 C028155.1 C028156.1 C028157.1 C028158.1 C028159.1 C028160.1 C028161.1 C028162.1 C028163.1
0
5K
10K
15K
20K
25K
V. planifolia
V. planifolia
f

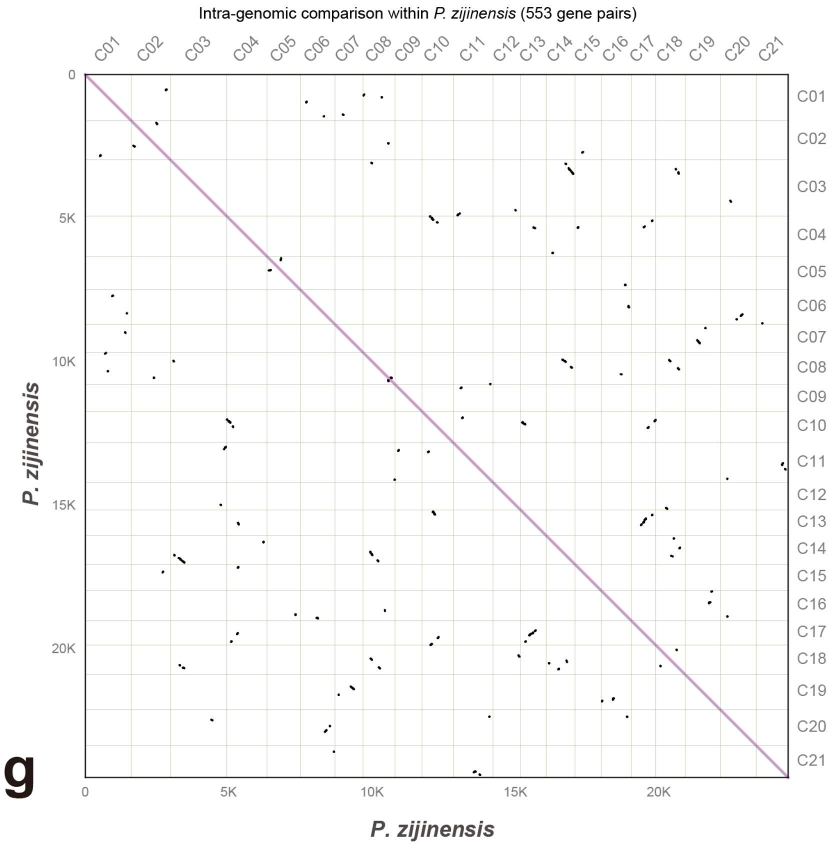
Intra-genomic comparison within P. zijinensis (553 gene pairs)
C01 C02 C03 C04 C05 C06 C07 C08 C09 C10 C11 C12 C13 C14 C15 C16 C17 C18 C19 C20 C21
P. zijinensis
0
5K
10K
15K
20K
g
P. zijinensis

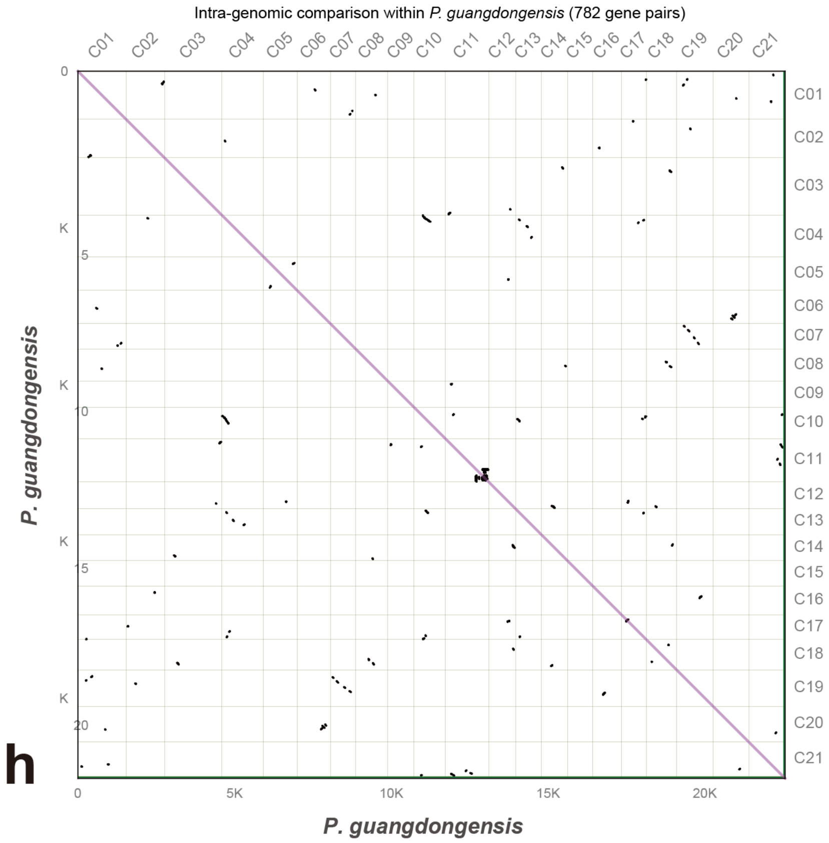
Intra-genomic comparison within P. guangdongensis (782 gene pairs)
C01 C02 C03 C04 C05 C06 C07 C08 C09 C10 C11 C12 C13 C14 C15 C16 C17 C18 C19 C20 C21
P. guangdongensis
0
5K
10K
15K
20K
h
P. guangdongensis

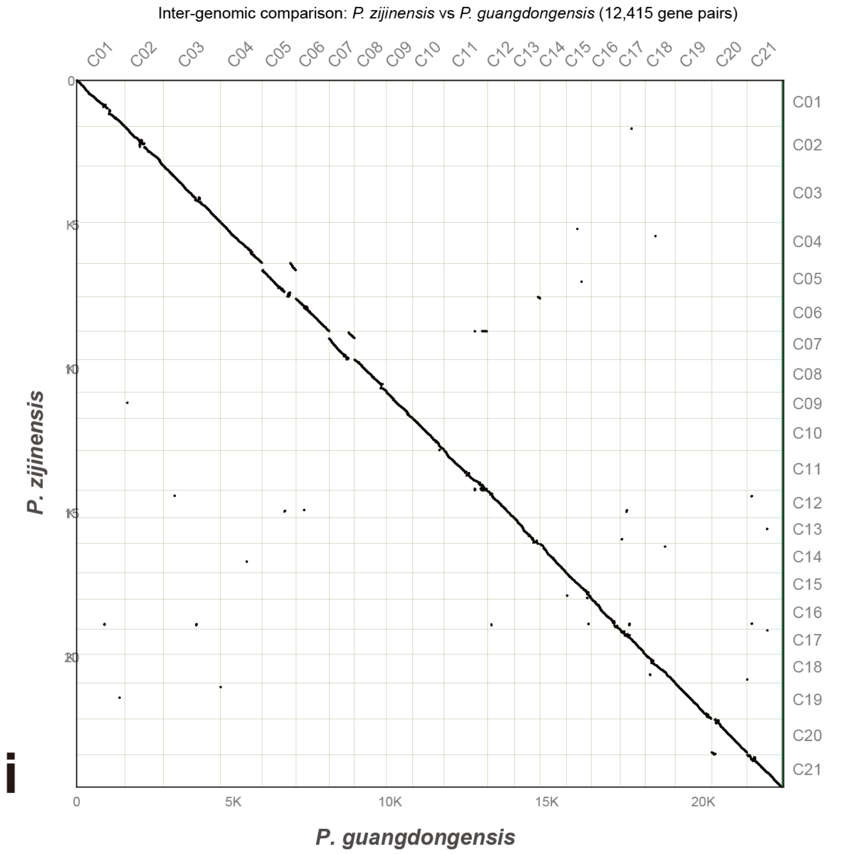

Supplementary Figure 6. Syntenic block of *V. planifolia*, *P. zijinensis*, *P. guangdongensis* and *D. chrysotoxum*. a. *V. planifolia* vs. *P. zijinensis*. **b.** *D. chrysotoxum* vs. *P. zijinensis.* **c**. *V. planifolia* vs. *P. guangdongensis.* **d**. *D. chrysotoxum* vs. *P. guangdongensis*. **e**. *D. chrysotoxum* vs. *D. chrysotoxum*. **f.** *V. planifolia* vs. *V. planifolia.* **g.** *P. zijinensis* vs. *P. zijinensis.* **h.** *P. guangdongensis* vs. *P. guangdongensis*. **i.** *P. zijinensis* vs. *P. guangdongensis*.

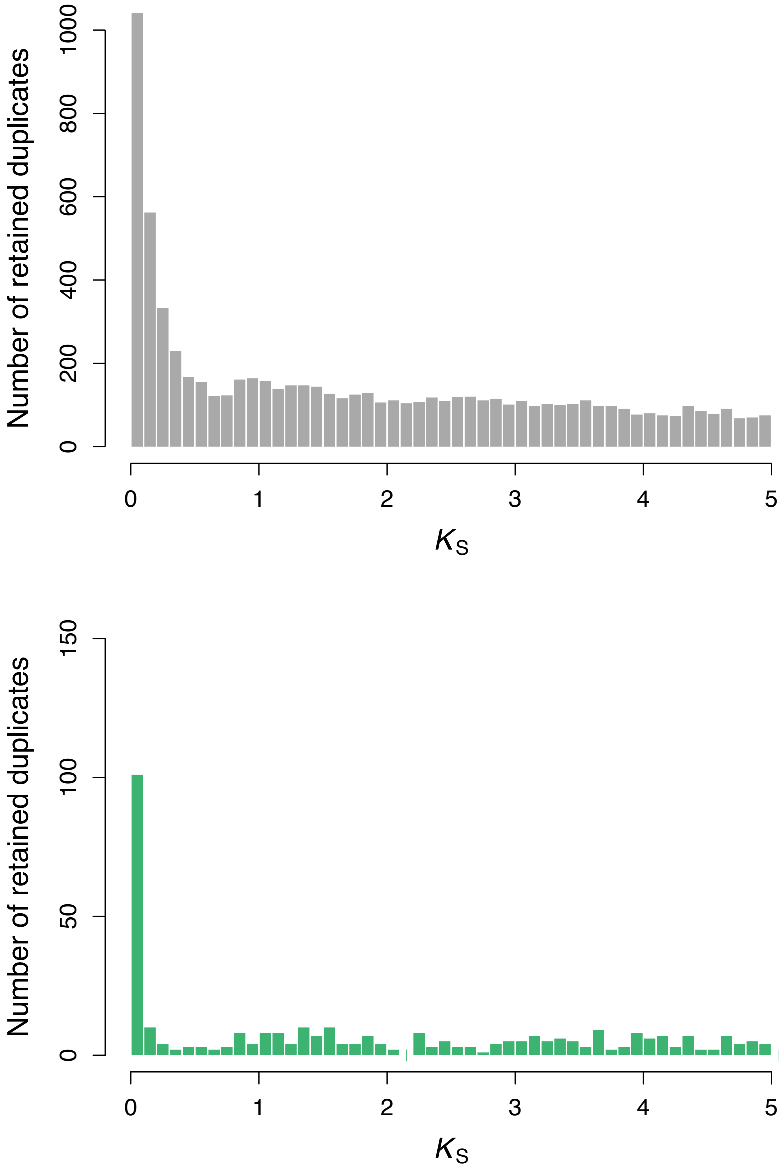

Supplementary Figure 7. *K*s distributions for the whole paranome (up) and the anchor pairs (bottom) in the *P. zijinensis* genome.

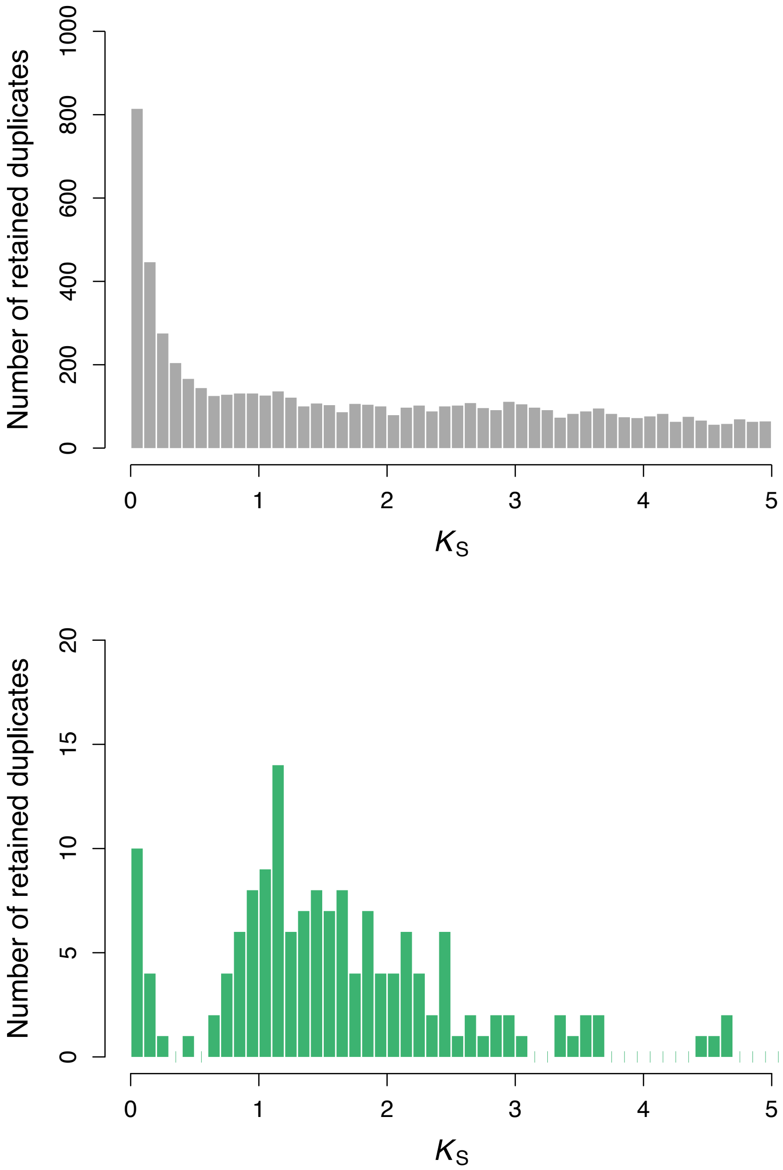

Supplementary Figure 8. *K*S distributions for the whole paranome (up) and the anchor pairs (bottom) in the *P. guangdongensis* genome.

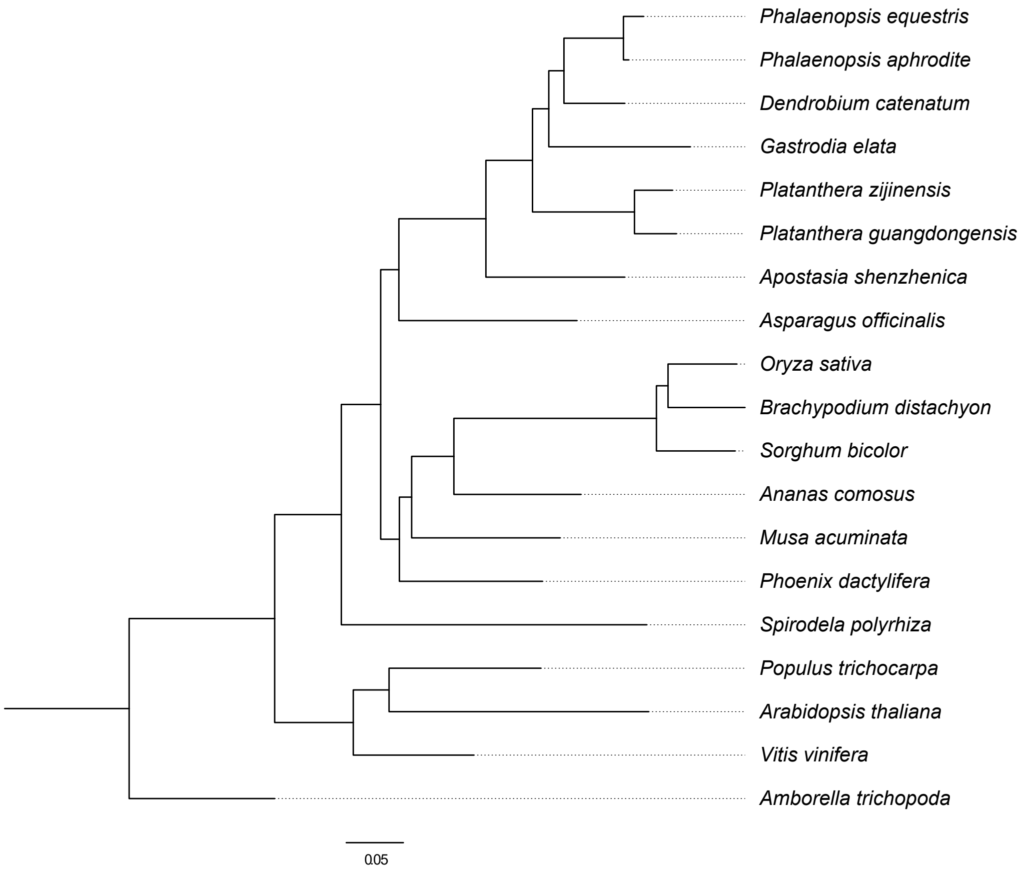

Supplementary Figure 9. Maximum likelihood phylogram of 19 angiosperms inferred based on single-copy genes. The phylogram shows that *P. zijinensis* and *P. guangdongensis* may have higher substitution rate than other sequenced orchids.

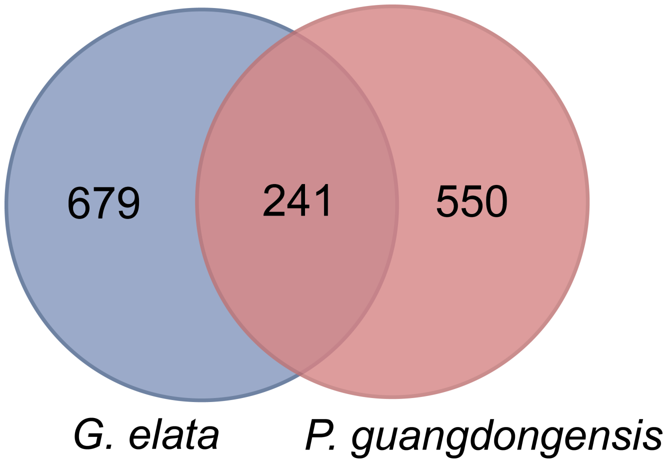

Supplementary Figure 10. Venn diagram indicates the common lost genes of *G. elata* and *P. guangdongensis*.

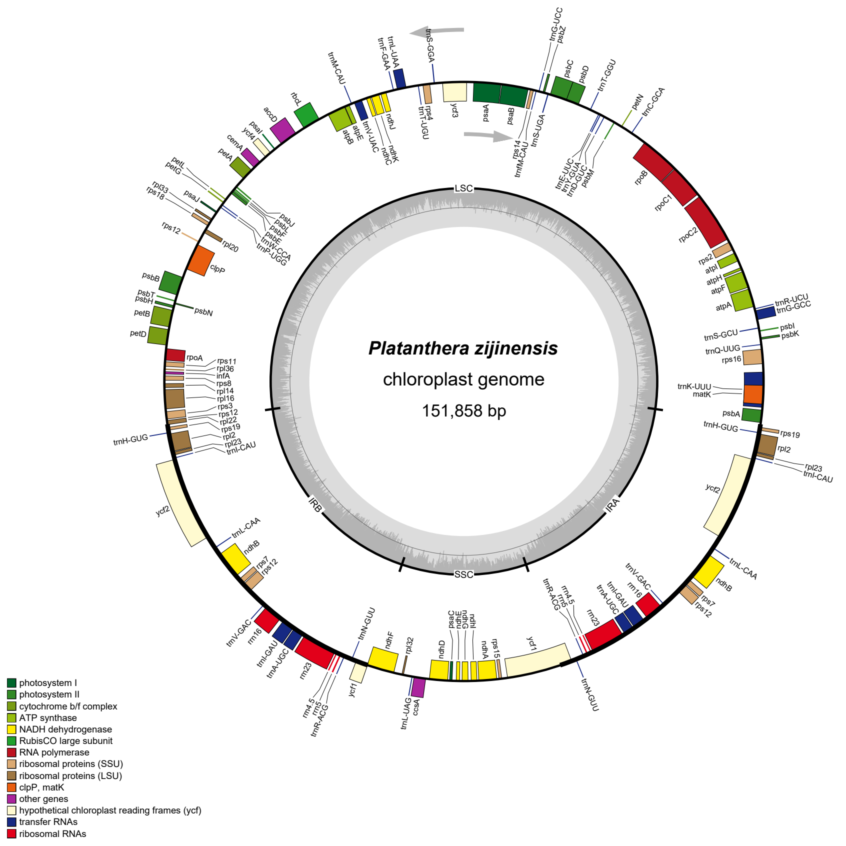

Supplementary Figure 11. Chloroplast genome of *P. zijinensis*. The total length of *P. zijinensis* chloroplast genome is 151,858 bp with 128 genes in total; the overall GC content is 36.78%.

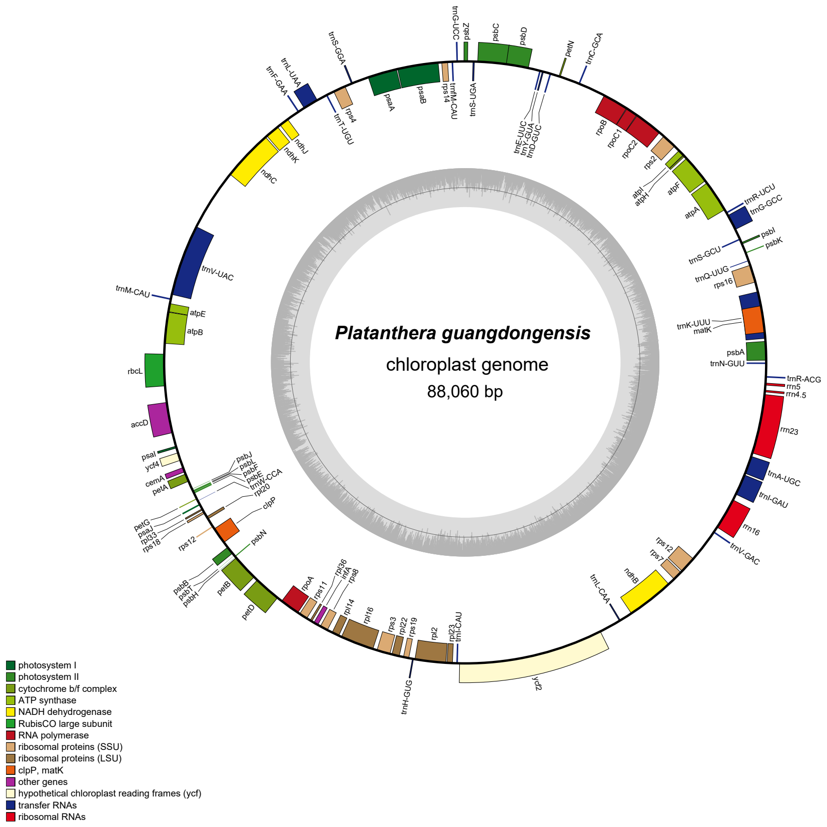

Supplementary Figure 12. Chloroplast genome of *P. guangdongensis*. The total length of *P. guangdongensis* chloroplast genome is 88,060 bp with 60 genes in total; the overall GC content is 35.99%.

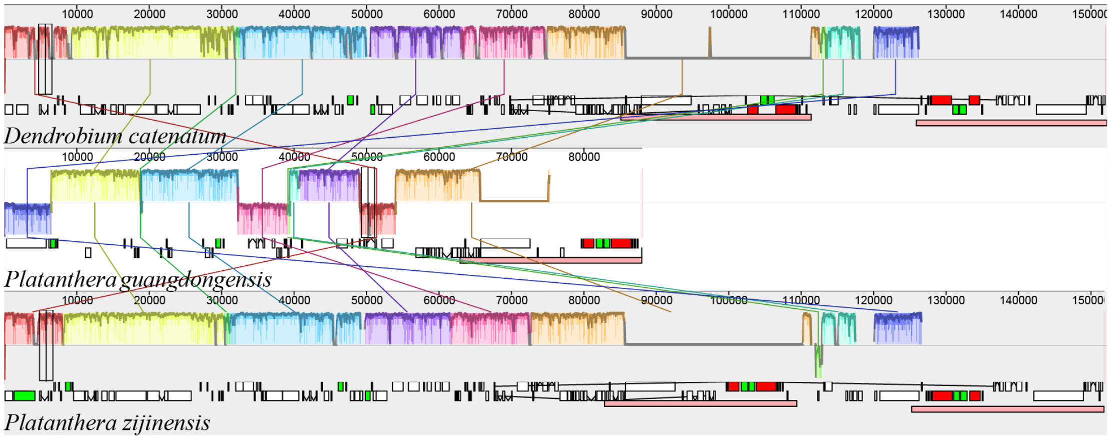

Supplementary Figure 13. Chloroplast genome collinearity mapping of *D. catenatum*, *P. guangdongensis*, and *P. zijinensis*.

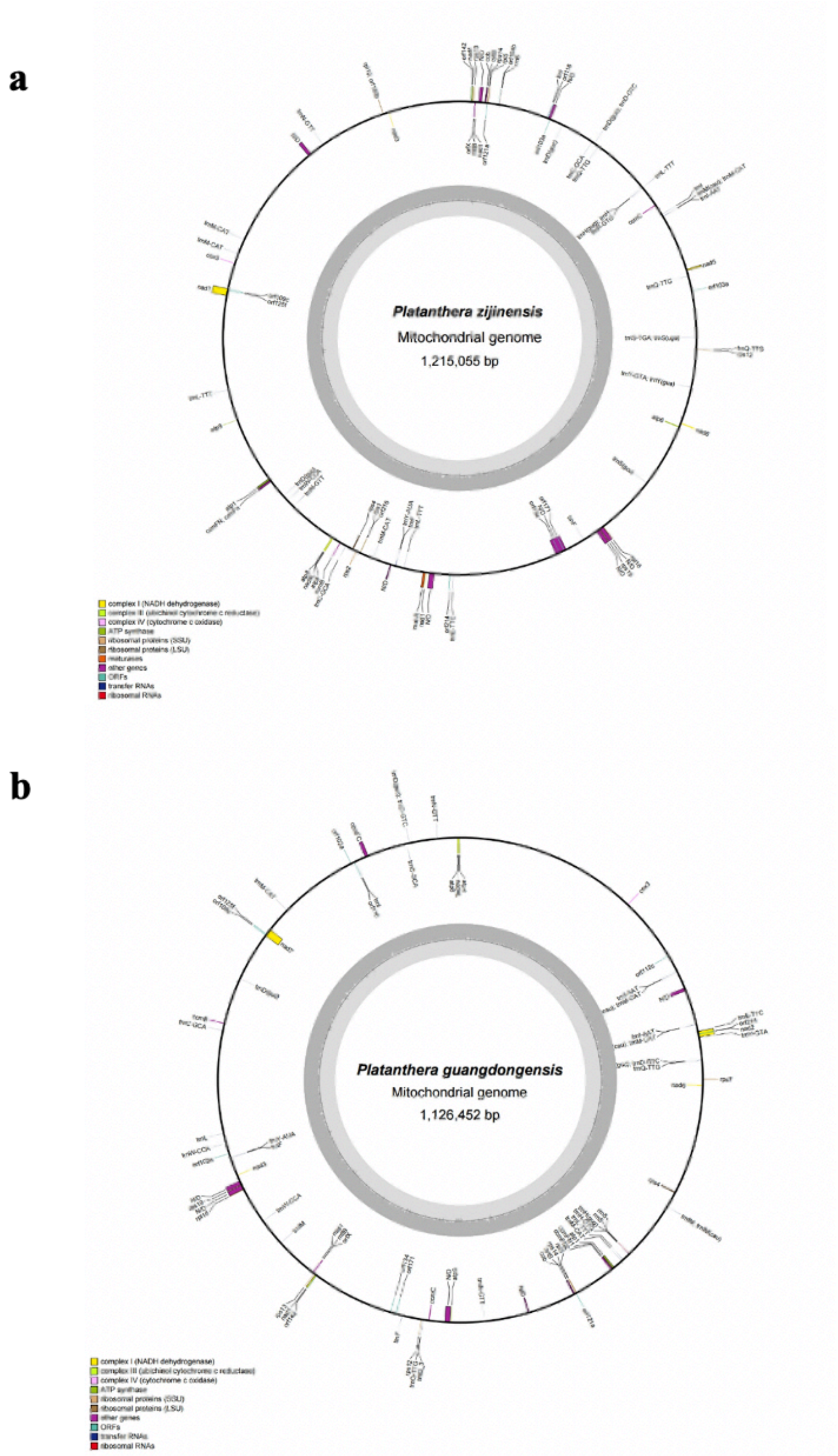

Supplementary Figure 14. Mitochondrial genome of *P. zijinensis* and *P. guangdongensis*. a. The mitochondrial genome size of *P. zijinensis* is 1,215kb, with 37 coding genes, 20 tRNA, and one rRNA. **b.** The mitochondrial genome size of *P. guangdongensis* is 1,126kb, with 33 coding genes, 19 tRNA, and one rRNA.

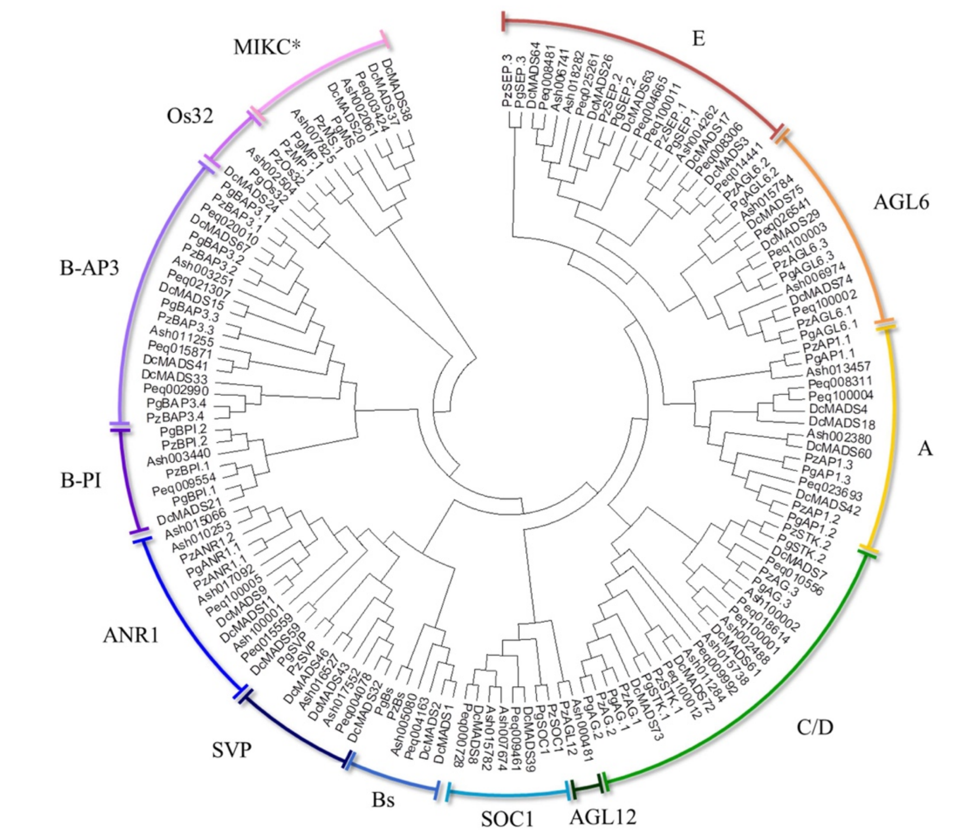

Supplementary Figure 15. Phylogenetic analysis of type II MADS-box genes in orchids. Phylogenetic analysis of type II MADS-box genes based on *P. guangdongensis*, *P. zijinensis*, *A. shenzhenica* (Ash), *D. catenatum* (Dc), and *Pha. equestris* (Pe).

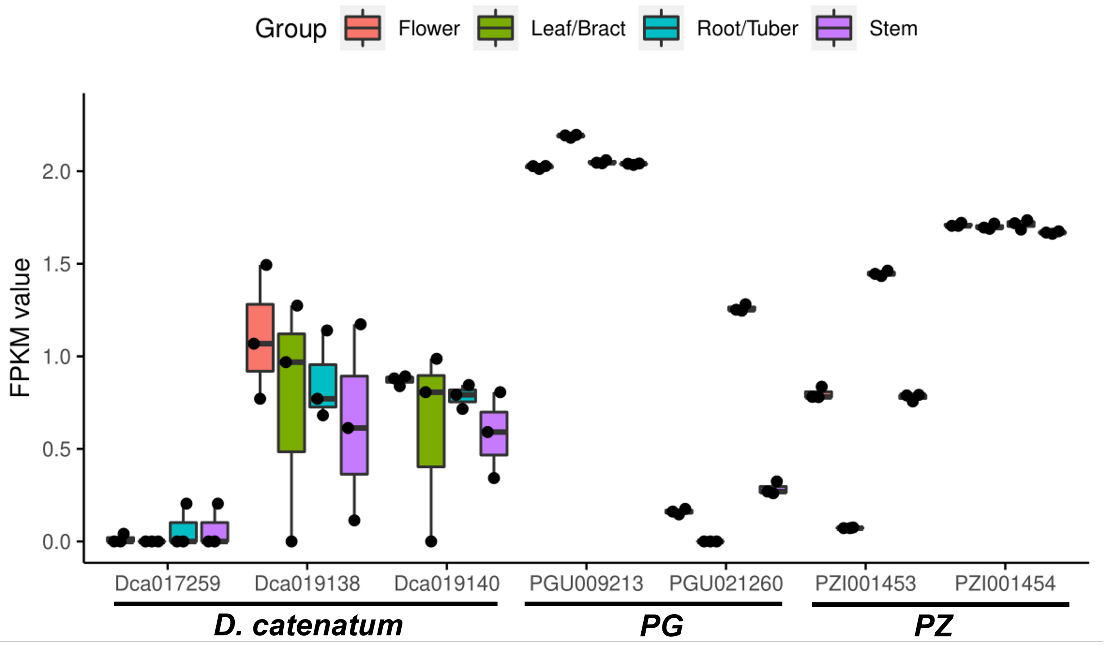

Supplementary Figure 16. Expression patterns of trehalase genes in various organs of autotrophic orchid *D. catenatum*, fully mycoheterotrophic orchid *P. guangdongensis* (PG), and partially mycoheterotrophic orchid *P. zijinensis* (PZ), which are performed in three replicates. The line in the middle of a box represents the median value and the up and bottom borders of the boxes denote the 75th and 25th percentiles, respectively. The upper and lower bars show the largest value within 1.5 times interquartile range above 75th percentile and the smallest value within 1.5 times interquartile range below 25th percentile, respectively. A dot shows the corresponding data points.

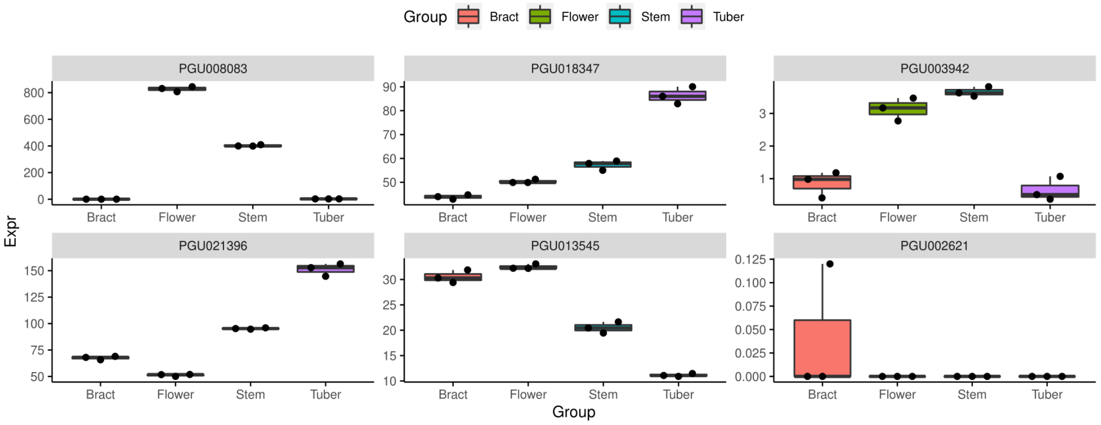

Supplementary Figure 17. Expression patterns of SUT genes in various organs of *P. guangdongensis*, which are performed in three replicates. The line in the middle of a box represents the median value and the up and bottom borders of the boxes denote the 75th and 25th percentiles, respectively. The upper and lower bars show the largest value within 1.5 times interquartile range above 75th percentile and the smallest value within 1.5 times interquartile range below 25th percentile, respectively. A dot shows the corresponding data points.

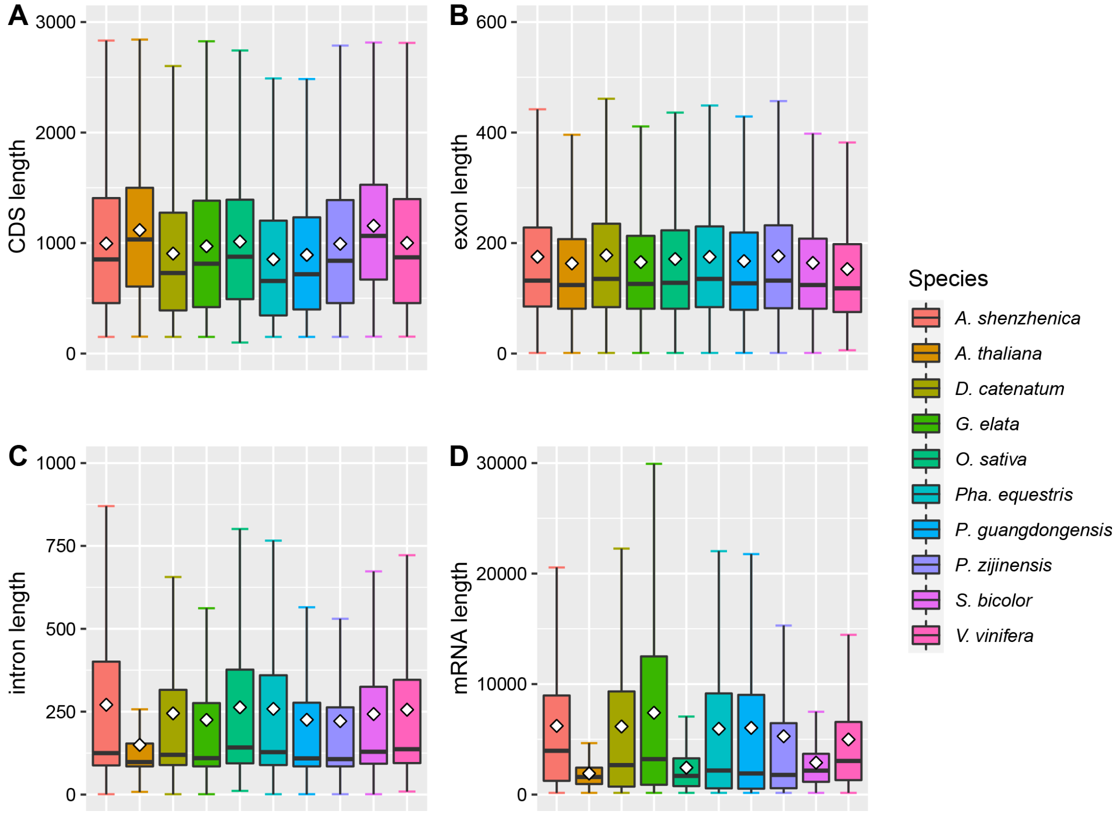

Supplementary Figure 18. The boxplot of gene elements length for ten plant species. The white rhombus on the box represents the means of the length. The detailed length was listed in **Supplementary Table 45.**

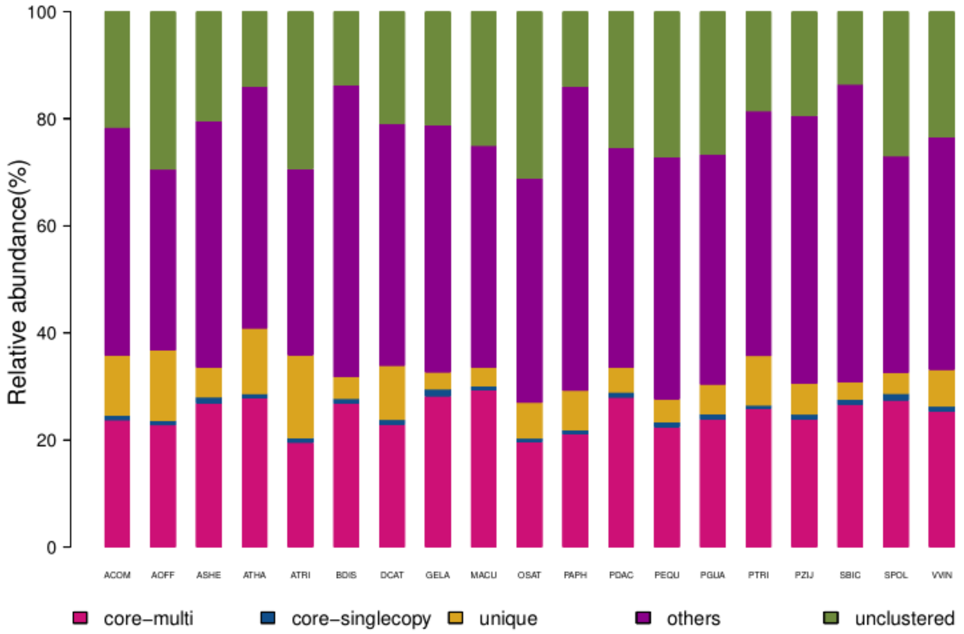

Supplementary Figure 19. Ortholog groups found in various plant species. Core-multi: genes that have orthologs in all other species and might have paralogs in species within one family. Core-single copy: genes that have orthologs in all other species and no other paralogs in this species within one family. Unique: genes for which only one family contains genes of this species. Other orthologs: genes that are not included in the other mentioned categories. Non-clustered genes: genes that are not clustered into any family. ACOM, *A. comosus*; ASHE, *A. shenzhenica*; AOFF, *A. officinalis*; ATHA, *Ar. thaliana*; ATRI, *Am. trichopoda*; BDIS, *B. distachyon*; DCAT, *D. catenatum*; EROS, *E. roseum*; GELA, *G. elata*; MACU, *M. acuminata*; OSAT, *O. sativa*; PDAC, *Pho. dactylifera*; PEQU, *Pha. equestris*; PGUA, *P. guangdongensis*; PTRI, *Po. trichocarpa*; PZIJ, *P. zijinensis*; SBIC, *S. bicolor*; SPOL, *Sp. polyrrhiza*; VVIN, *Vi. vinifera*.

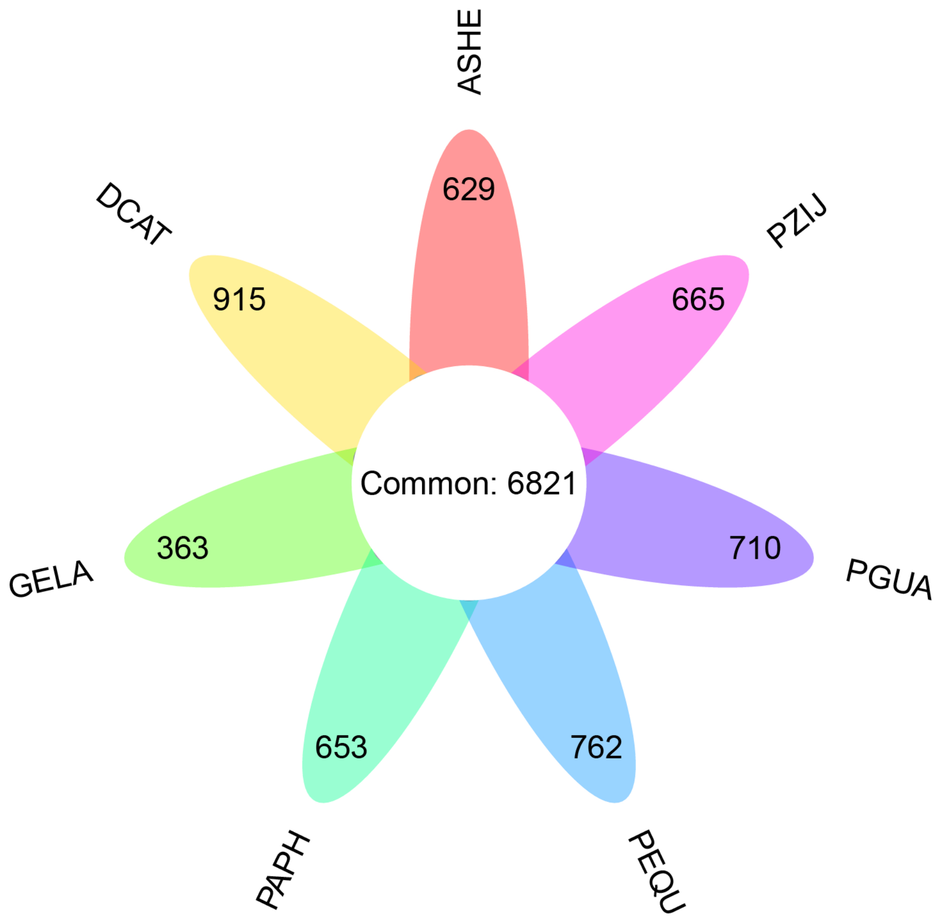

Supplementary Figure 20. A seven-way comparison of *A. shenzhenica* (ASHE), *D. catenatum* (DCAT), *G. elata* (GELA), *Pha. aphrodite* (PAPH), *Pha. equestris* (PEQU), *P. guangdongensis* (PGUA) and *P. zijinensis* (PZIJ) in Orchidaceae. A total of 6,821 gene families were shared by all taxa, with 710 and 665 gene families unique to *P. guangdongensis* and *P. zijinensis*, respectively, 915 in *D. catenatum*, and 363 in *G. elata*.

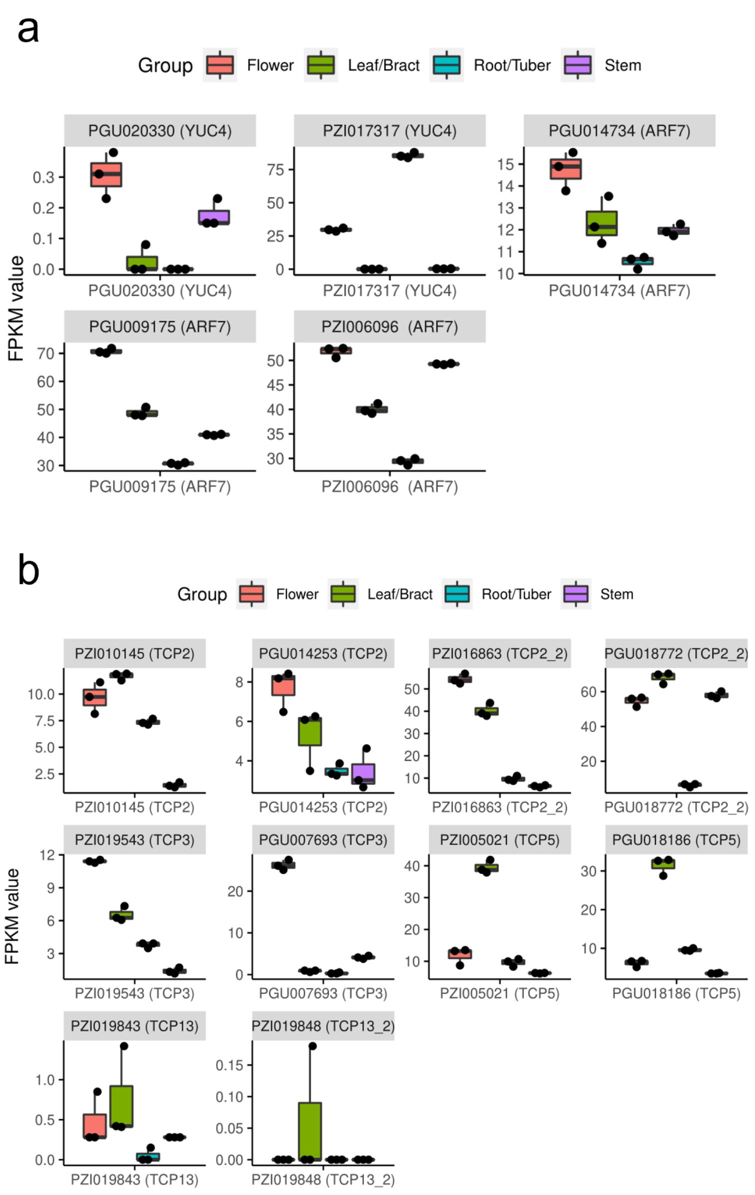

Supplementary Figure 21. Expression pattern of transcriptome analysis for leaf development-related genes. a. *YUC* and *ARF7* expression levels in *P. zijinensis* (PZJ) and *P. guangdongensis* (PGU). *YUC* was expressed in almost all tissues of *P. zijinensis*, whereas no expression was noted in *P. guangdongensis*. *P. guangdongensis* has two *ARF7* genes, whereas *P. zijinensis* has only one. **b.** TCP expression level in *P. zijinensis* and *P. guangdongensis*. *P. zijinensis* has six *TCP* genes, and *P. zijinensis* and *P. guangdongensis* have different expression levels in *TCP2*, which are performed in three replicates. The line in the middle of a box represents the median value and the up and bottom borders of the boxes denote the 75th and 25th percentiles, respectively. The upper and lower bars show the largest value within 1.5 times interquartile range above 75th percentile and the smallest value within 1.5 times interquartile range below 25th percentile, respectively. A dot shows the corresponding data point.

Supplementary Tables

Supplementary Table 1. The statistics of *P. zijinensis* sequencing raw data from Pacific Biosciences platforms.

ID	ZMWNUM	Total bases (Gb)	Total reads	Average length (bp)	Max length (bp)	N50 length (bp)
r54160_20190712_093131-1_G01	727,183	25.93	1,377,670	18,821.57	211,197	26,689
r54263_20190703_090130-1_D01	584,835	20.73	1,057,822	19,599.44	211,348	27,257
r54263_20190706_083155-1_G01	694,124	23.75	1,297,184	18,312.31	210,386	26,287
r54266_20190710_093754-1_B01	660,948	23.62	1,219,016	19,377.48	221,776	26,926
r54267_20190710_093813-1_C01	622,237	21.70	1,134,772	19,123.47	190,359	26,868
r54268_20190708_093835-1_F01	690,376	25.06	1,323,320	18,939.90	203,496	26,919
r54269_20190708_094938-1_G01	677,439	23.87	1,262,466	18,903.56	208,356	26,598
r64048_20190713_082126-1_A01	5,071,152	126.55	6,618,643	19,120.94	159,011	28,192
r64048_20190715_031525-1_A01	6,015,591	149.95	8,068,149	18,585.13	155,047	27,085
Total	-	441.16	23,359,042	18,886.49	221,776	27,267

Supplementary Table 2. The statistics of *P. guangdongensis* sequencing raw data from Pacific Biosciences platforms.

ID	ZMWNUM	Total bases (Gb)	Total reads	Average length (bp)	Max length (bp)	N50 length (bp)
r54254_20190514_085037-1_C01	710,566	12.21	1,312,476	9,304.96	87,046	16,860
r54254_20190515_083958-1_D01	626,440	8.97	910,747	9,848.73	96,340	16,697
r54254_20190515_083958-2_E01	735,998	10.01	1,054,511	9,491.23	82,712	15,882
r54266_20190516_092754-1_E01	712,758	21.06	1,830,262	11,504.34	193,579	19,002
r54266_20190518_094033-1_F01	690,430	18.82	1,670,219	11,269.93	215,838	18,739
r54266_20190520_084709-1_B01	702756	21.14	1,706,490	12,389.31	192,367	19,309
r54268_20190518_094350-1_G01	696,836	21.26	1,781,783	11,934.14	210,180	19,119
r54040_20190712_093002-1_F01	718,228	29.32	1,617,479	18,127.80	201,512	23,708
r54267_20190708_093608-1_E01	663,825	25.35	1,402,189	18,079.00	190,768	23,605
r54268_20190702_090150-1_F01	768,250	18.34	1,244,058	14,742.10	110,636	20,952
r54268_20190703_090404-1_B01	706,350	28.79	1,581,022	18,209.23	225,546	23,956
r54268_20190705_085134-1_G01	763,760	29.79	1,685,557	17,675.49	197,044	23,420
r54269_20190705_085250-1_F01	712,663	28.45	1,585,848	17,941.25	231,293	23,809
r54270_20190707_083441-1_G01	723,633	29.66	1,598,055	18,559.75	211,480	24,046
r64048_20190716_084325-1_A01	3,668,388	111.03	5,736,032	19,357.11	139,943	26,271
Total	-	414.201	26,716,728	15,503.95	231,293	22,824

Supplementary Table 3. Summary of the *P. zijinensis* and *P. guangdongensis* genomes' assembly.

		Contig		Scaffold	
		Size (bp)	Number	Size (bp)	Number
P. zijinensis	N90	670,212	2,146	670,212	2,146
	N80	944,401	1,626	944,401	1,626
	N70	1,204,585	1,234	1,204,585	1,234
	N60	1,462,795	917	1,462,795	917
	N50	1,766,898	658	1,766,898	658
	Longest	14,726,023	-	14,726,023	-
	Total Length	4,185,530,821	-	4,185,530,821	-
	Total Number(>=100bp)	-	3,363	-	3,363
	Total Number(>=2000bp)	-	3,363	-	3,363
	GC Content	0.434	-	0.434	-
P. guangdongensis	N80	682,374	1,916	682,374	1,916
	N70	954,322	1,399	954,322	1,399
	N60	1,219,324	1,009	1,219,324	1,009
	N50	1,567,289	704	1,567,289	704
	Longest	18,894,801	-	18,894,801	-
	Total Length	4,197,037,508	-	4,197,037,508	-
	Total Number(>=100bp)	-	5,632	-	5,632
	Total Number(>=2000bp)	-	5,632	-	5,632
	GC Content	0.444	-	0.444	-

Supplementary Table 4. BUSCO assessment of genome assembly for *P. zijinensis* and *P. guangdongensis*.

Species	Type	Number	Percentage
P. zijinensis	Complete BUSCOs（C）	1,431	88.66%
	Complete and single-copy BUSCOs (S)	1,336	82.78%
	Complete and duplicated BUSCOs (D)	95	5.89%
	Fragmented BUSCOs (F)	59	3.66%
	Missing BUSCOs (M)	124	7.68%
	Total BUSCO groups searched	1,614	-
P. guangdongensis	Complete BUSCOs（C）	1,163	72.06%
	Complete and single-copy BUSCOs (S)	1,029	63.75%
	Complete and duplicated BUSCOs (D)	134	8.30%
	Fragmented BUSCOs (F)	127	7.87%
	Missing BUSCOs (M)	324	20.07%
	Total BUSCO groups searched	1,614	-

Supplementary Table 5. Chromosome length (bp) of *P. zijinensis* and *P. guangdongensis*.

P. zijinensis		***P. guangdongensis***	
**Chromosome ID**	**Length(bp)**	**Chromosome ID**	**Length(bp)**
Chr01	288,559,838	Chr01	306,176,568
Chr02	288,230,612	Chr02	289,869,818
Chr03	259,891,334	Chr03	268,315,559
Chr04	243,465,763	Chr04	237,903,340
Chr05	217,914,638	Chr05	230,796,450
Chr06	213,067,135	Chr06	200,407,056
Chr07	201,669,873	Chr07	196,390,169
Chr08	201,248,329	Chr08	195,397,838
Chr09	192,346,260	Chr09	193,144,512
Chr10	191,405,230	Chr10	192,856,783
Chr11	187,582,923	Chr11	192,089,829
Chr12	181,146,792	Chr12	184,075,117
Chr13	180,270,012	Chr13	174,593,638
Chr14	176,476,062	Chr14	174,014,280
Chr15	175,863,887	Chr15	172,265,668
Chr16	175,754,357	Chr16	171,788,109
Chr17	175,677,710	Chr17	169,190,934
Chr18	172,175,136	Chr18	167,679,590
Chr19	161,383,232	Chr19	156,112,666
Chr20	157,638,113	Chr20	148,208,908
Chr21	144,783,085	Chr21	143,092,320

Supplementary Table 6. Summary of the *P. zijinensis* and *P. guangdongensis* genomes Hi-C assembly.

Species		Contig		Scaffold	
		Size (bp)	Number	Size (bp)	Number
P. zijinensis	N90	662,872	2,185	161,383,232	19
	N50	1,736,073	681	192,346,260	9
	Total Length	4,184,852,821	-	4,186,550,321	-
P. guangdongensis	N90	362,532	2,856	156,112,666	19
	N50	1,450,518	764	193,144,512	9
	Total Length	4,161,505,152	-	4,164,369,152	-

Supplementary Table 7. Categories of TEs predicted in *P. zijinensis*, *P. guangdongensis* and *G. elata* genomes

		RepBase TEs		TE Proteins		De novo		Combined TEs	
**Species**		**Length(bp)**	**% in Genome**	**Length(bp)**	**% in Genome**	**Length(bp)**	**% in Genome**	**Length(bp)**	**% in Genome**
	DNA	4,996,793	0.12	1,050,501	0.03	65,487,478	1.56	71,534,772	1.71
	LINE	1,578,911	0.04	443,274	0.01	21,621,402	0.52	23,643,587	0.56
	SINE	46,606	0.0011	0	0	1600	0.000038	48,206	0.0012
P. zijinensis	LTR	850,067,254	20.31	1,144,610,665	27.35	2,971,093,848	70.98	3,105,436,035	74.19
	LTR/Copia	426,528,277	10.19	580,166,597	13.86	1,335,154,123	31.90	1,422,324,008	33.98
	LTR/Gypsy	418,558,969	10.00	563,872,943	13.47	1,500,933,102	35.86	1,582,140,725	37.80
	Other	415	0.000010	0	0	0	0	415	0.000010
	Unknown	0	0	0	0	38,239,108	0.91	38,239,108	0.91
	Total	856,689,979	20.47	1,146,104,440	27.38	3,096,443,436	73.98	3,238,902,123	77.38
	DNA	2,919,630	0.069564	2,229,489	0.053121	90,468,666	2.155536	95,617,785	2.278221
	LINE	1,526,718	0.036376	995,739	0.023725	18,219,059	0.434093	20,741,516	0.494194
	SINE	64,580	0.001539	0	0	0	0	64,580	0.001539
P. guangdongensis	LTR	936,968,234	22.324514	1,153,597,873	27.486003	3,276,163,435	78.058951	3,297,634,853	78.570536
	LTR/Copia	498,706,193	11.882338	608,203,565	14.491259	1,435,667,013	34.206676	1,500,619,947	35.754266
	LTR/Gypsy	432,901,969	10.314465	545,368,605	12.994132	1,550,522,207	36.943254	1,573,200,149	37.483586
	Other	795	0.000019	0	0	0	0	795	0.000019
	Unknown	0	0	0	0	35,068,008	0.835542	35,068,008	0.835542
	Total	941,479,957	22.432012	1,156,823,101	27.562849	3,419,919,168	81.484122	3,449,127,537	82.180050
	DNA	17,593,554	1.66	216,595	0.02	43,345,030	4.09	47,093,040	4.44
	LINE	20,300,857	1.91	811,853	0.08	28,687,422	2.7	43,878,465	4.14
	SINE	7,750	0	0	0	121,164	0.01	128,645	0.01
G. elata	LTR	93,802,600	8.84	119,788,504	11.29	607,792,416	57.29	620,760,349	58.51
	Other	1,566	0	0	0	0	0	1,566	0
	Unknown	22,732	0	84	0	11,625,819	1.1	11,648,578	1.1
	Total	123,015,109	11.59	120,816,935	11.39	669,540,327	63.11	686,052,705	64.66

Supplementary Table 8. The subtypes of repeats for six orchid species (see separate files).

Supplementary Table 9. LTR insertion distribution of *P. zijinensis*.

Insertion time (Mya)	LTR number	LTR percent (%)	Cumulative percent (%)
0.0-0.1	3912	10.21	100.0
0.1-0.2	3963	10.34	89.79
0.2-0.3	3691	9.63	79.45[a]
0.3-0.4	3120	8.14	69.82
0.4-0.5	2507	6.54	61.68
0.5-0.6	2298	6.0	55.14
0.6-0.7	1840	4.8	49.14
0.7-0.8	1674	4.37	44.34
0.8-0.9	1649	4.3	39.97
0.9-1.0	1161	3.03	35.67
1.0-1.1	1289	3.36	32.64
1.1-1.2	1114	2.91	29.27
1.2-1.3	994	2.59	26.37
1.3-1.4	954	2.49	23.77
1.4-1.5	766	2.0	21.28
1.5-1.6	912	2.38	19.29
1.6-1.7	699	1.82	16.91
1.7-1.8	735	1.92	15.08
1.8-1.9	651	1.7	13.16
1.9-2.0	567	1.48	11.47
2.0-2.1	563	1.47	9.99
2.1-2.2	426	1.11	8.52
2.2-2.3	414	1.08	7.41
2.3-2.4	322	0.84	6.33
2.4-2.5	304	0.79	5.48
2.5-2.6	256	0.67	4.69
2.6-2.7	230	0.6	4.02
2.7-2.8	178	0.46	3.42
2.8-2.9	172	0.45	2.96
>2.9	962	2.51	2.51

[a]The percentage of insertion of *P. zijinensis* before 0.2 Mya is 79.45%.

Supplementary Table 10. LTR insertion distribution of *P. guangdongensis*.

Insertion time (Mya)	LTR number	LTR percent (%)	Cumulative percent (%)
0.0-0.1	217	0.6	100.0
0.1-0.2	885	2.47	99.4
0.2-0.3	1234	3.44	96.93
0.3-0.4	2100	5.85	93.49
0.4-0.5	2206	6.15	87.63
0.5-0.6	2420	6.75	81.48
0.6-0.7	2517	7.02	74.74
0.7-0.8	2428	6.77	67.72
0.8-0.9	2539	7.08	60.95[a]
0.9-1.0	2175	6.06	53.87
1.0-1.1	2187	6.1	47.81
1.1-1.2	1927	5.37	41.71
1.2-1.3	1657	4.62	36.34
1.3-1.4	1420	3.96	31.72
1.4-1.5	1288	3.59	27.76
1.5-1.6	1264	3.52	24.17
1.6-1.7	1041	2.9	20.65
1.7-1.8	907	2.53	17.75
1.8-1.9	775	2.16	15.22
1.9-2.0	645	1.8	13.06
2.0-2.1	646	1.8	11.26
2.1-2.2	492	1.37	9.46
2.2-2.3	467	1.3	8.09
2.3-2.4	329	0.92	6.79
2.4-2.5	322	0.9	5.87
2.5-2.6	277	0.77	4.97
2.6-2.7	271	0.76	4.2
2.7-2.8	194	0.54	3.44
2.8-2.9	196	0.55	2.9
>2.9	845	2.36	2.36

[a]The percentage of insertion of *P. guangdongensis* before 0.8Mya is 60.95%.

Supplementary Table 11. The prediction of gene structures of *P. zijinensis*, *P. guangdongensis* and *G. elata*.

Species	Gene set	Number	Average gene length (bp)	Average CDS length (bp)	Average exon per gene	Average exon length (bp)	Average intron length (bp)
	denovo/AUGUSTUS	270,300	3,372.18	720.59	2.18	330.46	2,246.09
	denovo/GlimmerHMM	194,383	19,235.5	914.68	4.93	185.37	4,656.54
	denovo/Genscan	113,289	10,045.67	594.28	2.98	199.51	4,776.65
	homo/*P. equestris*	27,579	15,046.22	830.92	3.31	251.02	6,153.47
	homo/*D. catenatum*	27,603	15,210.25	872.2	3.35	260.72	6,113.36
P. zijinensis	homo/*A. shenzhenica*	25,770	15,646.02	884.21	3.38	261.51	6,199.57
	homo/*G. elata*	24,351	15,712.72	871.46	3.46	252.2	6,044.28
	homo/*A. officinalis*	22,300	15,981.18	840.68	3.55	236.83	5,938.18
	homo/*A. comosus*	26,258	15,758.85	808.65	3.31	244.3	6,471.84
	RNA-seq	14,355	38,035.39	1,276.58	5.72	223.37	7,796.12
	MAKER	41,538	13,481.62	902.62	3.56	301.68	4,845.78
	Final	24,513	27,223.47	1,142.58	4.77	280.61	6,857.84
	denovo/AUGUSTUS	80,639	10,761.39	572.84	2.61	219.52	6,330.33
	denovo/GlimmerHMM	85,653	47,365.03	653.75	7.61	85.95	7,070.96
	denovo/Genscan	39,054	20,307.14	373.02	2.55	146.48	12,889.19
	homo/*P. equestris*	27,456	17,423.78	763.59	3.28	232.73	7,303.68
	homo/*D. catenatum*	27,205	17,936.26	794.4	3.34	237.9	7,328.08
	homo/*A. shenzhenica*	25,044	19,318.27	809.67	3.44	235.62	7,596.85
P. guangdongensis	homo/*G. elata*	23,657	19,502.75	820.35	3.55	231.14	7,328.78
	homo/*A. officinalis*	22,565	19,814.04	774.62	3.57	217.01	7,409.88
	homo/*A. comosus*	26,230	18,380.85	761.92	3.33	228.82	7,562.75
	RNA-seq	12,320	41,683.68	1,220.35	5.6	217.88	8,794.36
	MAKER	32,886	19,878.32	760.31	3.99	233.17	6,335.05
	Final	22,840	28,993.55	976.5	4.75	237.49	7,439.22
	After Hic	22559	29,014.56	980.89	4.76	237.86	7,412.71
	Augustus	21,936	13,514.25	884.15	3.97	222.61	4,250.07
	GlimmerHMM	55,024	17,429.62	561.02	3.83	146.59	5,966.72
	D. catenatum	22,201	8,433.42	877.26	3.64	240.77	2,858.41
	P. equestris	21,383	8,126.88	839.22	3.61	232.45	2,791.94
	A. shenzhenica	19,456	9,394.11	907.7	3.92	231.38	2,903.26
	A. officinalis	17,385	9,930.39	888.46	4.14	214.69	2,881.12
G. elata	*O. sativa*	18,788	8,149.08	797.42	3.63	219.54	2,792.90
	A. thaliana	16,118	10,187.20	908.57	4.16	218.58	2,939.32
	RNA-seq	12,186	19,373.64	1,092.56	4.79	228.23	3,354.41
	CEGMA	451	20,418.01	1,211.74	8.31	145.73	2,625.65
	MAKER	21,179	15,895.13	992.42	5.07	255.48	3,589.66
	Final set	18,019	15,982.61	1,091.86	5.18	253.51	3,331.50

Supplementary Table 12. Gene function annotation of *P. zijinensis* and *P. guangdongensis*.

Species	Values	Total	Nr	Swissprot	KEGG	KOG	TrEMBL	Interpro	GO	Overall
P. zijinensis	Number	25,511	21,908	16,346	16,695	16,311	21,919	20,106	4,229	22,237
	Percentage	-	85.88%	64.07%	65.44%	63.94%	85.92%	78.81%	16.58%	87.17%
P. guangdongensis	Number	21,725	18,441	12,010	13,500	13,092	18,284	15,609	3,404	18,698
	Percentage	-	84.88%	55.28%	62.14%	60.26%	84.16%	71.85%	15.67%	86.07%

Supplementary Table 13. Summary of ncRNA annotation of *P. zijinensis* and *P. guangdongensis*.

Species	Type		Copy(w)	Average length(bp)	Total length(bp)	% of genome
P. zijinensis	miRNA		31	139.70	4,331	0.000103
	tRNA		994	73.50	73,064	0.001745
	rRNA	rRNA	4,187	103.51	433,418	0.010353
		18S	44	1,176.7	51,778	0.001237
		28S	29	193.06	5,599	0.000134
		5.8S	21	153.76	3,229	0.000077
		5S	4,093	91.08	372,812	0.008905
	snRNA	snRNA	615	109.8	67,527	0.001613
		CD-box	531	105.98	56,278	0.001344
		HACA-box	10	115.8	1,158	0.000028
		splicing	74	136.36	10,091	0.000241
		scaRNA	0	0	0	0
P. guangdongensis	miRNA		33	124.52	4,109	0.000098
	tRNA		1,015	73.44	74,546	0.001774
	rRNA	rRNA	2,533	192.55	487,721	0.011608
		18S	307	912.08	280,008	0.006664
		28S	140	189.83	26,576	0.000633
		5.8S	66	153.15	10,108	0.000241
		5S	2,020	84.67	171,029	0.004071
	snRNA	snRNA	152	114.70	17434	0.000415
		CD-box	73	103.82	7,579	0.00018
		HACA-box	14	129.93	1,819	0.000043
		splicing	65	123.63	8,036	0.000191
		scaRNA	0	0.00	0	0

Supplementary Table 14. BUSCO assessment of genome annotation in six orchids.

Species	Type	Number	Percentage
P. zijinensis	Complete BUSCOs (C)	1,288	79.80%
	Complete and single-copy BUSCOs (S)	1,200	74.35%
	Complete and duplicated BUSCOs (D)	88	5.45%
	Fragmented BUSCOs (F)	101	6.26%
	Missing BUSCOs (M)	225	13.94%
	Total BUSCO groups searched	1,614	-
P. guangdongensis	Complete BUSCOs (C)	949	58.80%
	Complete and single-copy BUSCOs (S)	913	55.57%
	Complete and duplicated BUSCOs (D)	36	2.23%
	Fragmented BUSCOs (F)	177	10.97%
	Missing BUSCOs (M)	488	30.24%
	Total BUSCO groups searched	1,614	-
G. elata	Complete BUSCOs (C)	1,061	65.74%
	Complete and single-copy BUSCOs (S)	1,035	64.13%
	Complete and duplicated BUSCOs (D)	26	1.61%
	Fragmented BUSCOs (F)	103	6.38%
	Missing BUSCOs (M)	450	27.88%
	Total BUSCO groups searched	1,614	-
A. shenzhenica	Complete BUSCOs (C)	1,339	82.96%
	Complete and single-copy BUSCOs (S)	1,318	81.66%
	Complete and duplicated BUSCOs	21	1.30%
	Fragmented BUSCOs (F)	135	8.36%
	Missing BUSCOs (M)	140	8.67%
	Total BUSCO groups searched	1,614	-
Pha. equestris	Complete BUSCOs (C)	1,240	76.82%
	Complete and single-copy BUSCOs (S)	1,211	75.03%
	Complete and duplicated BUSCOs (D)	29	1.80%
	Fragmented BUSCOs (F)	197	12.21%
	Missing BUSCOs (M)	177	10.97%
	Total BUSCO groups searched	1,614	-
D. catenatum	Complete BUSCOs (C)	1,242	76.95%
	Complete and single-copy BUSCOs (S)	1,210	74.97%
	Complete and duplicated BUSCOs (D)	32	1.98%
	Fragmented BUSCOs (F)	176	10.90%
	Missing BUSCOs (M)	196	12.14%
	Total BUSCO groups searched	1,614	-

Supplementary Table 15. Statistics of syntenic analysis in *P. guangdongensis*, *P. zijinensis*, *Pha. aphrodite, V. planifolia* and *D. chrysotoxum*.

Species1	Species2	#Genes	#Collinear genes	Percentage (%)	#Collinear blocks	#MeanGene in blocks
P. guangdongensis	*P. guangdongensis*	22,559	911	4.04	66	11.8
P. zijinensis	*P. zijinensis*	24,513	857	3.50	68	8.1
P. guangdongensis	*P. zijinensis*	47,072	29,645	62.98	138	146.9
P. guangdongensis	*Pha. aphrodite*	42,232	18,518	43.85	450	26.4
P. zijinensis	*Pha. aphrodite*	44,186	19,428	43.97	485	25.8
P. guangdongensis	*D. chrysotoxum*	52,604	22,367	42.52	481	30.6
P. guangdongensis	*V. planifolia*	51,726	18,868	36.48	990	13.9
P. zijinensis	*D. chrysotoxum*	54,558	24,305	44.55	599	27.3
P. zijinensis	*V. planifolia*	53,680	19,664	36.63	1,024	14.4

Supplementary Table 16. Summary of orthologous gene families in 19 sequenced plant species.

Species	Genes	Unclustered genes	Clustered genes	Families	Unique families	Unique families genes	Common families	Common families genes	Single copy	Average genes per family
A. comosus	27,024	5,841	21,183	12,939	777	3,043	3,607	6,645	234	1.637
A. officinalis	27,375	8,071	19,304	11,831	827	3,590	3,607	6,471	234	1.632
A. shenzhenica	20,560	4,200	16,360	11,780	328	1,145	3,607	5,754	234	1.389
A. thaliana	26,637	3,714	22,923	12,438	776	3,250	3,607	7,633	234	1.843
A. trichopoda	25,933	7,615	18,318	12,082	942	4,003	3,607	5,296	234	1.516
B. distachyon	26,415	3,632	22,783	14,738	375	1,070	3,607	7,320	234	1.546
D. catenatum	26,791	5,603	21,188	13,663	678	2,717	3,607	6,367	234	1.551
G. elata	18,019	3,823	14,196	10,530	213	573	3,607	5,312	234	1.348
M. acuminata	34,241	8,574	25,667	12,505	479	1,209	3,607	10,290	234	2.053
O. sativa	35,402	11,034	24,368	15,766	907	2,374	3,607	7,181	234	1.546
Pha. aphrodite	28,910	4,046	24,864	13,717	414	2,121	3,607	6,327	234	1.813
Pho. dactylifera	23,890	6,079	17,811	10,800	368	1,086	3,607	6,912	234	1.649
Pha. equestris	26,471	7,206	19,265	13,985	467	1,147	3,607	6,169	234	1.378
P. guangdongensis	22,559	6,012	16,547	12,014	470	1,248	3,607	5,610	234	1.377
Po. trichocarpa	40,984	7,614	33,370	14,054	1,239	3,789	3,607	10,849	234	2.374
P. zijinensis	24,513	4,764	19,749	12,539	487	1,418	3,607	6,072	234	1.575
S. bicolor	27,160	3,691	23,469	15,093	330	913	3,607	7,476	234	1.555
S. polyrhiza	18,357	4,959	13,398	10,020	241	713	3,607	5,266	234	1.337
V. vinifera	25,328	5,953	19,375	12,536	595	1,741	3,607	6,651	234	1.546

Supplementary Table 17. GO enrichment analysis for missing gene families in *S. polyrhiza.*

GO ID	GO Term	GO Class	Pvalue	AdjustedPv	x1	x2	n	N	GOlevl
GO:0003824	catalytic activity	MF	1.08E-10	2.26E-07	621	8,744	1,530	26,471	2
GO:0008152	metabolic process	BP	3.63E-09	7.55E-06	725	10,658	1,530	26,471	2
GO:0005488	binding	MF	1.49E-08	3.09E-05	750	11,160	1,530	264,71	2
GO:0043169	cation binding	MF	2.68E-08	5.59E-05	227	2,771	1,530	26,471	4
GO:0046872	metal ion binding	MF	3.17E-08	6.59E-05	225	2,747	1,530	26,471	5
GO:0046914	transition metal ion binding	MF	5.13E-08	0.000107	151	1,692	1,530	26,471	6
GO:0071704	organic substance metabolic process	BP	5.18E-08	0.000108	579	8,361	1,530	26,471	3
GO:0044699	single-organism process	BP	6.90E-08	0.000144	901	13,857	1,530	26,471	2
GO:0016787	hydrolase activity	MF	1.19E-07	0.000248	272	3,502	1,530	26,471	3
GO:0009055	electron carrier activity	MF	1.34E-07	0.000278	59	497	1,530	26,471	2
GO:0044238	primary metabolic process	BP	2.71E-07	0.000564	559	8,123	1,530	26,471	3
GO:0044710	single-organism metabolic process	BP	9.48E-07	0.001973	310	4,178	1,530	26,471	3
GO:0009987	cellular process	BP	1.32E-06	0.002747	873	13,556	1,530	26,471	2
GO:0016568	chromatin modification	BP	1.10E-05	0.022848	10	33	1,530	26,471	6
GO:0042393	histone binding	MF	1.26E-05	0.026279	8	21	1,530	26,471	4
GO:0005515	protein binding	MF	1.78E-05	0.037066	275	3,772	1,530	26,471	3
GO:0005507	copper ion binding	MF	2.05E-05	0.042664	18	102	1,530	26,471	7

Supplementary Table 18. GO enrichment analysis for missing gene families in *Pho. dactylifera.*

GO ID	GO Term	GO Class	Pvalue	AdjustedPv	x1	x2	n	N	GOlevl
GO:0016407	acetyltransferase activity	MF	2.46E-13	5.25E-10	22	57	1,530	26,471	6
GO:0008080	N-acetyltransferase activity	MF	3.30E-11	7.04E-08	17	41	1,530	2,6471	7
GO:0016747	transferase activity, transferring acyl groups other than amino-acyl groups	MF	1.95E-07	0.000414	29	172	1,530	26,471	5
GO:0016746	transferase activity, transferring acyl groups	MF	4.98E-06	0.010612	32	233	1,530	26,471	4
GO:1901360	organic cyclic compound metabolic process	BP	8.37E-06	0.017822	319	4,427	1,530	26,471	4
GO:0044710	single-organism metabolic process	BP	9.03E-06	0.019239	303	4,178	1,530	26,471	3
GO:0044699	single-organism process	BP	1.03E-05	0.021868	882	13,857	1,530	26,471	2
GO:0003824	catalytic activity	MF	1.23E-05	0.026188	582	8,744	1,530	26,471	2
GO:0006807	nitrogen compound metabolic process	BP	2.14E-05	0.045514	350	4,973	1,530	26,471	3

Supplementary Table 19. GO enrichment analysis for missing gene families in *P. guangdongensis*.

GO ID	GO Term	GO Class	Pvalue	AdjustedPv	x1	x2	n	N	GOlevl
GO:0016491	oxidoreductase activity	MF	4.00E-08	6.69E-05	141	2418	982	26,471	3
GO:0009654	photosystem II oxygen evolving complex	CC	1.34E-07	0.000223	7	13	982	26,471	3
GO:0015979	photosynthesis	BP	1.35E-07	0.000225	16	88	982	26,471	4
GO:0009523	photosystem II	CC	7.01E-07	0.001173	8	22	982	26,471	4
GO:0009521	photosystem	CC	1.11E-06	0.001858	11	48	982	26,471	3
GO:0044436	thylakoid part	CC	3.52E-06	0.005891	12	64	982	26,471	4
GO:0042651	thylakoid membrane	CC	7.47E-06	0.012497	8	29	982	26,471	4
GO:0044699	single-organism process	BP	9.55E-06	0.015973	580	13,857	982	26,471	2
GO:0055114	oxidation-reduction process	BP	2.12E-05	0.035419	123	2,296	982	26,471	4
GO:0019898	extrinsic component of membrane	CC	2.24E-05	0.037393	6	17	982	26,471	3
GO:0051260	protein homooligomerization	BP	2.66E-05	0.044499	4	6	982	26,471	7

Supplementary Table 20. GO enrichment analysis for missing gene families in *G. elata*.

GO ID	GO Term	GO Class	Pvalue	AdjustedPv	x1	x2	n	N	GOlevl
GO:0015979	photosynthesis	BP	1.87E-17	3.16E-14	28	88	1,127	26,471	4
GO:0009765	photosynthesis, light harvesting	BP	1.99E-13	3.36E-10	10	11	1,127	26,471	5
GO:0009521	photosystem	CC	4.02E-13	6.79E-10	18	48	1,127	26,471	3
GO:0044436	thylakoid part	CC	1.07E-12	1.80E-09	20	64	1,127	26,471	4
GO:0009654	photosystem II oxygen evolving complex	CC	4.79E-12	8.08E-09	10	13	1,127	26,471	3
GO:0009507	chloroplast	CC	7.77E-12	1.31E-08	11	17	1,127	26,471	6
GO:0034357	photosynthetic membrane	CC	8.00E-12	1.35E-08	19	63	1,127	26,471	3
GO:0051537	2 iron, 2 sulfur cluster binding	MF	1.61E-11	2.72E-08	10	14	1,127	26,471	5
GO:0009523	photosystem II	CC	3.64E-10	6.13E-07	11	22	1,127	26,471	4
GO:0042651	thylakoid membrane	CC	8.87E-10	1.50E-06	12	29	1,127	26,471	4
GO:1990204	oxidoreductase complex	CC	1.03E-09	1.73E-06	14	42	1,127	26,471	3
GO:0051536	iron-sulfur cluster binding	MF	1.98E-09	3.34E-06	17	67	1,127	26,471	4
GO:0019684	photosynthesis, light reaction	BP	5.52E-09	9.32E-06	11	27	1,127	26,471	5
GO:0016491	oxidoreductase activity	MF	8.48E-08	0.000143	156	2,418	1,127	26,471	3
GO:0015035	protein disulfide oxidoreductase activity	MF	1.04E-07	0.000176	14	58	1,127	26,471	6
GO:0019898	extrinsic component of membrane	CC	1.82E-07	0.000306	8	17	1,127	26,471	3
GO:0010277	chlorophyllide a oxygenase [overall] activity	MF	3.27E-06	0.005515	4	4	1,127	26,471	6
GO:0042440	pigment metabolic process	BP	4.21E-06	0.007101	8	24	1,127	26,471	4
GO:0016020	membrane	CC	5.21E-06	0.00879	496	9,976	1,127	26,471	2
GO:0009522	photosystem I	CC	5.24E-06	0.00884	7	18	1,127	26,471	4
GO:0003824	catalytic activity	MF	6.46E-06	0.010898	441	8,744	1,127	26,471	2
GO:0019062	virion attachment to host cell	BP	7.49E-06	0.01264	38	412	1,127	26,471	6
GO:0045454	cell redox homeostasis	BP	1.54E-05	0.025919	14	86	1,127	26,471	4
GO:0004960	thromboxane receptor activity	MF	1.86E-05	0.031346	17	123	1,127	26,471	8

Supplementary Table 21. Photosynthesis-related KEGG pathway and genes in six orchids.

	ko00196 gene	ko00196 KOs	ko00195 gene nuclear	ko00195 KOs nuclear	ko00195 gene chloroplast	ko00195 KOs chloroplast	ko00195 genes Total	ko00195 KOs Total
Pha. aphrodite	13	11	61	36	29	29	90	54
Pha. equestris	11	9	58	38	29	29	87	53
D. catenatum	16	11	53	31	28	28	81	50
G. elata	0	0	6	4	0	0	6	4
P. guangdongensis	6	6	55	25	3	3	58	27
P. zijinensis	15	9	94	44	30	30	124	54
A. shenzhenica	13	11	44	33	30	30	74	53
A. officinalis	18	12	37	21	27	27	64	46
B. distachyon	18	12	81	42	29	29	110	54
O. sativa	15	12	114	43	30	30	144	53
S. bicolor	17	12	51	33	29	29	80	54
A. comosus	20	12	62	31	30	30	92	51
Pho. dactylifera	18	10	30	17	32	30	62	47
M. acuminata	24	12	64	28	28	28	92	53
S. polyrhiza	17	11	27	18	28	28	55	46
Po. trichocarpa	26	12	129	43	30	30	159	54
A. thaliana	22	12	46	26	30	30	76	55
V. vinifera	17	11	43	30	30	30	73	52
A. trichopoda	21	12	50	32	30	30	80	55

ko00195: Photosynthesis. https://www.kegg.jp/kegg-bin/show_pathway?ko00195

ko00196: Photosynthesis - antenna proteins. https://www.kegg.jp/kegg-bin/show_pathway?ko00196

Supplementary Table 22. Chloroplast genes of *P. zijinensis* and *P. guangdongensis*.

Category for genes	Group of genes	Name of genes (*P. zijinensis*)	Name of genes (*P. guangdongensis*)
Self-replication	Ribosomal RNAs	*rrn4.5, rrn5, rrn16, rrn23*	*rrn4.5, rrn5, rrn16, rrn23*
	Transfer RNAs	$trnA^{UGC}$, $trnC^{GCA}$, $trnD^{GUC}$, $trnE^{UUC}$, $trnF^{GAA}$, $trnfM^{CAU}$, $trnG^{GCC}$, $trnG^{UCC}$, $trnH^{GUG}$, $trnI^{CAU}$, $trnI^{GAU}$, $trnK^{UUU}$, $trnL^{CAA}$, $trnL^{UAA}$, $trnL^{UAG}$, $trnM^{CAU}$, $trnN^{GUU}$, $trnP^{UGG}$, $trnQ^{UUG}$, $trnR^{ACG}$, $trnR^{UCU}$, $trnS^{GCU}$, $trnS^{GGA}$, $trnS^{UGA}$, $trnT^{GGU}$, $trnT^{UGU}$, $trnV^{GAC}$, $trnV^{UAC}$, $trnW^{CCA}$, $trnY^{GUA}$	$trnA^{UGC}$, $trnC^{GCA}$, $trnD^{GUC}$, $trnE^{UUC}$, $trnF^{GAA}$, $trnfM^{CAU}$, $trnG^{GCC}$, $trnG^{UCC}$, $trnH^{GUG}$, $trnI^{CAU}$, $trnI^{GAU}$, $trnL^{CAA}$, $trnL^{UAA}$, $trnL^{UAG}$, $trnM^{CAU}$, $trnN^{GUU}$, $trnP^{UGG}$, $trnQ^{UUG}$, $trnR^{ACG}$, $trnR^{UCU}$, $trnS^{GCU}$, $trnS^{GGA}$, $trnS^{UGA}$, $trnT^{UGU}$, $trnV^{GAC}$, $trnW^{CCA}$, $trnY^{GUA}$
	Small Ribosomal protein	*rps2, rps3, rps4, rps7, rps8, rps11, rps12, rps14, rps15, rps16, rps18, rps19*	*rps2, rps3, rps4, rps7, rps8, rps11, rps14, rps15, rps16, rps18, rps19*
	Large Ribosomal protein	*rpl2, rpl14, rpl16, rpl20, rpl22, rpl23, rpl32, rpl33, rpl36*	*rpl2, rpl14, rpl16, rpl20, rpl22, rpl23, rpl32, rpl33, rpl36*
	RNA polymerase	*rpoA, rpoB, rpoC1, rpoC2*	$rpoA^{\Psi}$
Genes for photosynthesis	Photosystem I	*psaA, psaB, psaC, psaI, psaJ*	$psaA^{\Psi}$, $psaB^{\Psi}$, *psaI*
	Photosystem II	*psbA, psbB, psbC, psbD, psbF, psbH, psbI, psbJ, psbK, psbL, psbM, psbN, psbT, psbZ*	$psbC^{\Psi}$, $psbD^{\Psi}$, $psbZ^{\Psi}$
	Cytochrome b/f complex	*petA, petB, petD, petG, petL, petN, psbE*	$petB^{\Psi}$, $petD^{\Psi}$, *petN*
	ATP synthase	*atpA, atpB, atpE, atpF, atpH, atpI*	$atpA^{\Psi}$, $atpB^{\Psi}$, $atpE^{\Psi}$, $atpF^{\Psi}$, $atpH^{\Psi}$, $atpI^{\Psi}$
	ATP-dependent protease subunit p gene	*clpP*	*clpP*
	RuBisCO large subunit	*rbcL*	$rbcL^{\Psi}$
	NADH dehydrogenase	$ndhA^{\Psi}$, *ndhB, ndhC*, $ndhD^{\Psi}$, *ndhE*, $ndhF^{\Psi}$, *ndhG*, $ndhI^{\Psi}$, $ndhJ^{\Psi}$, *ndhK*	$ndhB^{\Psi}$, $ndhJ^{\Psi}$, $ndhK^{\Psi}$
Other genes	Maturase	*matK*	*matK*

	Envelop membrane protein	*cemA*	
	Subunit of acetyl-CoA-carboxylase	*accD*	*accD*
	c-type cytochrome synthesis ccsA gene	*ccsA*	
	Translation initiation factor IF-1	*infA*	*infA*
Genes of unknown function	Hypothetical chloroplast reading frames	*ycf1, ycf2, ycf3, ycf4*	*ycf1, ycf2, ycf4*

Note: gene$^{\Psi}$ means Pseudogene.

Supplementary Table 23. GO enrichment analysis for missing gene families in *P. zijinensis*.

GO ID	GO Term	GO Class	Pvalue	AdjustedPv	x1	x2	n	N	GOlevl
GO:0044464	cell part	CC	7.15E-07	0.00109	215	6,908	617	26,471	2
GO:0043170	macromolecule metabolic process	BP	2.61E-06	0.003987	206	6,673	617	26,471	4
GO:0005622	intracellular	CC	5.15E-06	0.007847	161	4,996	617	26,471	3
GO:0019538	protein metabolic process	BP	6.27E-06	0.009558	110	3,126	617	26,471	4
GO:0005737	cytoplasm	CC	1.99E-05	0.030367	63	1,571	617	26,471	4
GO:0044238	primary metabolic process	BP	2.16E-05	0.032899	237	8,123	617	26,471	3
GO:0010467	gene expression	BP	2.23E-05	0.033973	134	4,103	617	26,471	5
GO:0044260	cellular macromolecule metabolic process	BP	3.10E-05	0.047303	175	5,699	617	26,471	4
GO:0016482	cytoplasmic transport	BP	3.17E-05	0.048416	10	86	617	26,471	5

Supplementary Table 24. GO enrichment analysis for missing gene families in *A. officinalis.*

GO ID	GO Term	GO Class	Pvalue	AdjustedPv	x1	x2	n	N	GOlevl
GO:0044260	cellular macromolecule metabolic process	BP	9.40E-07	0.001417	238	5,699	836	26,471	4
GO:0043170	macromolecule metabolic process	BP	2.55E-06	0.003835	269	6,673	836	26,471	4
GO:0065003	macromolecular complex assembly	BP	3.18E-05	0.047849	48	817	836	26,471	5

Supplementary Table 25. Significantly contracted gene families on the branch leading to the divergence between *P. zijinensis* and *P. guangdongensis* (see separate file).

Supplementary Table 26. Significantly expanded gene families on the branch leading to the divergence between *P. zijinensis* and *P. guangdongensis* (see separate file).

Supplementary Table 27. Significantly contracted gene families on the branch leading to *P. zijinensis* (see separate file).

Supplementary Table 28. Significantly expanded gene families on the branch leading to *P. zijinensis* (see separate file).

Supplementary Table 29. Significantly contracted gene families on the branch leading to *P. guangdongensis* (see separate file).

Supplementary Table 30. Significantly expanded gene families on the branch leading to *P. guangdongensis* (see separate file).

Supplementary Table 31. Number of genes related to leaf initiation and development in the genomes of *A. thaliana* and orchids*.

Category	*A. thaliana*	*A. shenzhenica*	*Pha. equestris*	*D. catenatum*	*P. zijinensis*	*P. guangdongensis*	*G. elata*
PLT3/5/7	3	1	3	1	2	0	1
PIN	8	5	6	5	7	1	3
PIN1	1	3	3	2	3	1	1
PINOID (PID)	1	1	1	3	1	1	2
ARF7/ARF19	2	1	1	4	1	1	1
YUC1/YUC4	2	1	1	1	1	1	0
Class I KNOX	4	3	3	5	3	2	2
AS1	1	1	1	1	1	1	1
AS2	1	3	3	5	1	1	2
ARF3/ARF4	2	1	2	1	1	1	1
KAN	4	5	4	4	5	3	3
CUC1/CUC2	2	4	5	2	1	1	1
Yabby	6	5	4	6	3	3	3
Class II CIN-TCP	8	7	9	10	6	4	3
RAX	3	3	5	5	3	3	3
GRF	9	8	8	9	11	9	5
GIF	3	1	1	2	1	1	2
WOX1/WOX3	2	0	0	2	2	2	2
SEU/SLK	4	2	3	4	3	3	2
LUG/LUH	2	1	2	2	2	2	1
Phytochrome	5	3	3	4	3	2	1
Cryptochrome	3	2	3	3	1	0	0
Phototropin	2	2	2	2	3	1	1

ARF: auxin-responsive factor; AS: ASYMMETRIC LEAVES; Class I KNOX: class-I KNOT-TED-like homeobox (STM, BP/KNAT1, KNAT2, KNAT6); CUC: CUP-SHAPED COTYLEDON; GIF: GRF-INTERACTING FACTOR; GRF: GROWTH-REGULATING FACTOR; KAN: KANADI; LUG: LEUNIG; LUH: LUG-HOMOLOG; PIN: PIN-FORMED; PLT: PLETHORA; RAX: REGULATOR OF AXILLARY MERISTEMS; SEU: SEUSS; SLK: SEU-like; WOX: WUSCHEL-RELATED HOMEOBOX; YUC: YUCCA

*genes ID see **Supplementary Table 46.**

Supplementary Table 32. Transcription factor genes involved in root development.

TF genes	*P. zijinensis*	*P. guangdongensis*	Family	Functions	References
BODENLOS (BDL)	PZI024281	PGU019826	AUX/IAA	Root meristem establishment	Hamann *et al*. (1999)[14]
	PZI011080	PGU014458			
	PZI013537				
SHOOTROOT (SHR)	PZI019997	PGU022539	GRAS	Root radial patterning	Benfey *et al.* (1993)[15]
SCARECROW (SCR)	PZI014099	PGU000238	GRAS	Root radial patterning; meristem maintenance	Scheres *et al*. (1995)[16]
TRANSPARENT TESTA GLABRA1 (TTG1)	PZI014628	PGU021885	WD40	Production of seed mucilage and anthocyanin pigments; epidermal cell patterning	Galway *et al*. (1994)[17] Walker *et al*. (1999)[18]
	PZI011377	PGU012877			
GLABRA3	PZI014668	PGU013160	bHLH	Epidermal cell patterning	Bernhardt *et al.* (2003)[19]
ENHANCER OF GLABRA3 (EGL3)	PZI014668	PGU013160	bHLH	Epidermal cell patterning	Bernhardt *et al.* (2003)[19]
WEREWOLF (WER)	PZI008325	PGU017605	MYB	Epidermal cell patterning	Lee and Schiefelbein (1999)[20]
	PZI000966	PGU008889			
	PZI017413	PGU011604			
CAPRICE (CPC)	PZI002055	not found	MYB	Epidermal cell patterning	Wada *et al*. (1997)[21]
TRIPTYCHON (TRY)					Schellmann *et al*. (2002)[22]
ENHANCER OF TRY AND CPC (ETC1)					Kirik *et al.* (2004)[23]

Supplementary Table 33. List of 46 MADS-box genes identified in *P. zijinensis*

Gene ID	Gene Name	ORF (bp)	Protein length (aa)	Type	Subfamily	Pseudogene
PZI013095	PzAG.1	708	235	MIKC^c	C/D	
PZI010938	PzAG.2	744	247	MIKC^c	C/D	
PZI006132	PzAG.3	705	234	MIKC^c	C/D	
PZI024426	PzSTK.1	753	250	MIKC^c	C/D	
PZI050004	PzSTK.2	681	226	MIKC^c	C/D	
PZI004437	PzBAP3.1	675	224	MIKC^c	B-AP3	
PZI022518	PzBAP3.2	678	225	MIKC^c	B-AP3	
PZI022957	PzBAP3.3	687	228	MIKC^c	B-AP3	
PZI050002	PzBAP3.4	654	217	MIKC^c	B-AP3	
PZI014560	PzBPI.1	636	211	MIKC^c	B-PI	
PZI004516	PzBPI.2	633	210	MIKC^c	B-PI	
PZI050003	PzBs	642	213	MIKC^c	Bs	
PZI005708	PzOs32	531	176	MIKC^c	OsMADS32	
PZI004828	PzSOC1	612	203	MIKC^c	SOC1	
PZI009972	PzSVP	603	200	MIKC^c	SVP	
PZI004642	PzAGL12	636	211	MIKC^c	AGL12	
PZI019581	PzANR1.1	696	231	MIKC^c	ANR1	
PZI010148	PzANR1.2	735	244	MIKC^c	ANR1	
PZI014789	PzAGL6.1	723	240	MIKC^c	AGL6	
PZI013076	PzAGL6.2	732	243	MIKC^c	AGL6	
PZI012696	PzAGL6.3	693	230	MIKC^c	AGL6	
PZI012079	PzAP1.1	741	246	MIKC^c	AP1	
PZI021295	PzAP1.2	750	249	MIKC^c	AP1	
PZI050001	PzAP1.3	732	243	MIKC^c	AP1	
PZI050005	PzSEP.1	735	244	MIKC^c	SEP	
PZI050006	PzSEP.2	732	243	MIKC^c	SEP	
PZI050007	PzSEP.3	726	241	MIKC^c	SEP	
PZI019012	PzMP.1	1,119	372	MIKC*	P	
PZI016288	PzMP.2	201	66	MIKC*	P	√
PZI020325	PzMS.1	1,011	336	MIKC*	S	
PZI020324	PzMS.2	1,293	430	MIKC*	S	√
PZI002440	PzMA.1	786	261	Type I	Mα	
PZI002483	PzMA.2	969	322	Type I	Mα	
PZI001747	PzMA.3	654	217	Type I	Mα	
PZI002441	PzMA.4	660	219	Type I	Mα	
PZI003343	PzMA.5	690	229	Type I	Mα	
PZI004140	PzMA.6	747	248	Type I	Mα	
PZI003345	PzMA.7	525	174	Type I	Mα	
PZI016009	PzMA.8	567	188	Type I	Mα	
PZI050008	PzMA.9	375	124	Type I	Mα	√
PZI001713	PzMA.10	354	117	Type I	Mα	

PZI019012	PzMA.11	972	323	Type I	Mα
PZI019880	PzMA.12	327	108	Type I	Mα
PZI007808	PzMC.1	471	156	Type I	Mγ
PZI007772	PzMC.2	684	227	Type I	Mγ
PZI050009	PzMC.3	366	121	Type I	Mγ

Supplementary Table 34. List of 47 MADS-box genes identified in *P. guangdongensis*

Gene ID	Gene Name	ORF (bp)	Protein length (aa)	Type	Subfamily	Pseudogene
PGU022789	PgAG.1	708	235	MIKC^c	C/D	
PGU020655	PgAG.2	843	280	MIKC^c	C/D	
PGU050001	PgAG.3	705	234	MIKC^c	C/D	
PGU018555	PgSTK.1	753	250	MIKC^c	C/D	
PGU001801	PgSTK.2	681	226	MIKC^c	C/D	
PGU011068	PgBAP3.1	675	224	MIKC^c	B-AP3	
PGU018698	PgBAP3.2	678	225	MIKC^c	B-AP3	
PGU019537	PgBAP3.3	684	227	MIKC^c	B-AP3	
PGU018266	PgBAP3.4	654	217	MIKC^c	B-AP3	
PGU006449	PgBPI.1	636	211	MIKC^c	B-PI	
PGU007229	PgBPI.2	633	210	MIKC^c	B-PI	
PGU014928	PgBs	717	238	MIKC^c	Bs	
PGU007237	PgOs32	600	199	MIKC^c	OsMADS32	
PGU002100	PgSOC1	612	203	MIKC^c	SOC1	
PGU004803	PgSVP	591	196	MIKC^c	SVP	
PGU001807	PgAGL12	267	88	MIKC^c	AGL12	√
PGU013474	PgANR1.1	696	231	MIKC^c	ANR1	
PGU050002	PgANR1.2	186	61	MIKC^c	ANR1	√
PGU008178	PgAGL6.1	723	240	MIKC^c	AGL6	
PGU021046	PgAGL6.2	732	243	MIKC^c	AGL6	
PGU002567	PgAGL6.3	696	231	MIKC^c	AGL6	
PGU050003	PgAP1.1	741	246	MIKC^c	AP1	
PGU013507	PgAP1.2	723	240	MIKC^c	AP1	
PGU016558	PgAP1.3	729	242	MIKC^c	AP1	
PGU016678	PgSEP.1	735	244	MIKC^c	SEP	
PGU018088	PgSEP.2	732	243	MIKC^c	SEP	
PGU010081	PgSEP.3	213	70	MIKC^c	SEP	
PGU006472	PgMP.1	1,023	340	MIKC*	P	
PGU011879	PgMP.2	201	66	MIKC*	P	√
PGU022296	PgMS	1,017	338	MIKC*	S	
PGU004531	PgMA.1	636	211	Type I	Mα	
PGU015179	PgMA.2	864	287	Type I	Mα	
PGU012136	PgMA.3	621	206	Type I	Mα	
PGU004532	PgMA.4	768	255	Type I	Mα	
PGU017676	PgMA.5	690	229	Type I	Mα	
PGU001885	PgMA.6	774	257	Type I	Mα	
PGU000760	PgMA.7	690	229	Type I	Mα	
PGU010479	PgMA.8	333	110	Type I	Mα	
PGU011598	PgMA.10	660	219	Type I	Mα	
PGU006472	PgMA.11	936	311	Type I	Mα	
PGU001756	PgMA.12	708	235	Type I	Mα	

PGU015396	PgMA.13	618	205	Type I	Mα	
PGU005410	PgMA.14	690	229	Type I	Mα	
PGU018662	PgMA.15	702	233	Type I	Mα	
PGU011841	PgMC.1	495	164	Type I	Mγ	
PGU011840	PgMC.2	321	106	Type I	Mγ	
PGU011522	PgMC.3	183	60	Type I	Mγ	√

Supplementary Table 35. Trehalase genes in sequenced plant genomes

Species	Cluster2514 - Trehalase
A. comosus	Aco013872
A. officinalis	evm.model.AsparagusV1_10.956
A. shenzhenica	Ash017765
A. thaliana	AT4G24040.1
A. trichopoda	evm_27.TU.AmTr_v1.0_scaffold00069.51
B. distachyon	Bradi3g31410.1
D. catenatum	Dca016005, Dca017749, Dca017750
G. elata	Gel001910, Gel008696, Gel012431, Gel014770
M. acuminata	GSMUA_Achr4P24850_001
O. sativa	Os10t0521000-01
Pha. aphrodite	PAXXG329540-mRNA1, PAXXG368690-mRNA1
Pho. dactylifera	PDK_30s879561g002
Pha. equestris	Peq014361
P. guangdongensis	PGU009213, PGU021260
Po. trichocarpa	Potri.001G087100.1, Potri.003G143900.1, Potri.003G144000.1
P. zijinensis	PZI001453, PZI001454
S. bicolor	Sb01g031280.1
S. polyrhiza	Spipo0020S28070.1
V. vinifera	GSVIVT01019610001

Supplementary Table 36. Number of genes related to nutrient transport and assimilation in the genomes of *A. thaliana* and orchids.

Category	*A. thaliana*	*P. zijinensis*	*P. guangdongensis*	*G. elata*	*Pha. equestris*	*D. catenatum*
AMT	6	3	2	1	3	6
NRT1[a]	17	21	14	13	19	20
NRT2	7	1	0	0	1	1
GLN	6	5	3	2	4	3
GDH	3	3	2	2	3	4
NIA	2	1	0	0	1[b]	1[b]
NIR	1	1	1	0	1	1
PHT1	9	5	5	2	4	7
PHT2	1	1	0	0	1	1

[a]: There are 53 members in Arabidopsis NRT1/PTR family. Seventeen out of them have been demonstrated to encode nitrate transporters. Only these 17 genes were used as queries to search orthologues in the orchid genomes[24-27]. [b]: Two fragment sequences were blasted to the C- and N-terminus of NIA, respectively. AMT: ammonium transporter family (AMT1;1-5/AMT2;1); GLN: glutamine synthase (GLN1;1-5/GLN2); GDH: glutamate dehydrogenase (GDH1/2/3); NIA: nitrate reductase (NIA1/2); NIR: nitrite reductase (NIR1); NRT1: low-affinity nitrate transporter family (exception of NRT1.1); NRT2: high-affinity nitrate transporter family. PHT: phosphate transporter family.

Supplementary Table 37. Sequences of trehalase genes in *C. goeringii* (see separate file).

Supplementary Table 38. Length distribution of gene elements in sequenced plants.

Species	Protein coding gene number	Average gene length (bp)	Average CDS length (bp)	Average exon per gene	Average exon length (bp)	Average intron length (bp)
P. zijinensis	24,513	27,223.47	1,142,58	4.77	280.60	6,857.84
P. guangdongensis	22,539	29,014.56	980.89	4.76	237.86	7,412.71
G. elata	18,019	15,982.61	1,091.86	5.18	253.51	3,331.50
A. shenzhenica	20,560	7,920.65	1,115.40	5.14	288.56	1,554.72
Pha. equestris	26,471	11,891.64	938.18	4.21	222.81	3,127.52
D. catenatum	26,791	10,832.91	1,004.40	4.28	234.68	2,714.53
Z. mays	38,510	3,965.59	1,101.62	4.54	242.75	637.83
S. bicolor	27,159	2,942.10	1,261.01	4.85	259.9	436.44
O. sativa	40,745	2,439.30	1,117.21	4.18	266.99	415.17
P. heterocycla	31,987	4,244.98	1,210.22	5.28	229.03	440.95
A. thaliana	26,637	1,909.57	1,242.78	5.23	237.5	157.54

Supplementary Table 39. Primers used in this study.

Gene ID	Species	Forward primer (5'->3')	Reverse primer (5'->3')
C232459_g1_i2	*C. goeringii*	AGTAACAACGACTGCGAGTATG	CGACAACCGAACCTGAGTTAT
C249696_g1_i4	*C. goeringii*	GTGGCTTGACTACTGGCTTAG	CCAATTCCACACGGGTATGA
c241934_g2_i1	*C. goeringii*	TTCTTCTCGTCACCATCAACTC	ACGTCTGCATCCTTGTCTTC
c241934_g3_i1	*C. goeringii*	GAAGCTCCCTGAGCATGTATC	CGTCTTCTCCTTCCCAATTTCT
Dca017701	*D. catenatum*	GGAGTTCATGCCTTTCTCCTTA	ACATTTGGGAGAGCAACATAGA
Dca000062	*D. catenatum*	AAGCTCGGGTACACACATTC	GAAGGCGAAGTGAGCCATTA
Dca006498	*D. catenatum*	CAAGGCTTCCCTTCGACTAAA	GGAGAATGGCATGAACTCTACA
Dca026994	*D. catenatum*	GTATTGGGTTTCCTGTTTGGATTAG	GTTCTTCTTCACCGCCTTCT
Dca021512	*D. catenatum*	CATCAGCATTGAGAACCCTTTG	GACTTCTTCCGGTACACCTTATAG
Dca006499	*D. catenatum*	GCAAGCTTAGCAGAGGGTAATA	CTAGGCTCATCTCATTGTTGGT
Dca024473	*D. catenatum*	CTCCTGCTGCCAAACATACT	CAACCTCAGGTCCCACTTTATC
Dca011010	*D. catenatum*	CATCTTCCTCAACGTCGCTATG	ACACAGATCCACCCGAGTATAA
Dca019140	*D. catenatum*	CAGAGACTGGTTGGGACTTTAG	GCTATGTCAAGCTCCACCTTAT
Dca019138	*D. catenatum*	GAAGTCCCTTCCTGCGTTAATA	GCGGCTCAAAGAATGTTTATGT
Dca017259	*D. catenatum*	GCTCTCGGTGGATGAGTAATG	GCTATGTCAAGCTCCACCTTAT
Ubiqutin	*D. catenatum*	CCGGATCAGCAAAGGTTGA	AAGATTTGCATCCCTCCCC

*Sequences of trehalase genes in *C. goeringii* are provided in **Supplementary Table 37**.

Supplementary Table 40. GO enrichment terms of significantly expanded gene families of *P. guangdongensis* (see separate file).

Supplementary Table 41. Mitochondrial genes of *P. zijinensis* and *P. guangdongensis*.

NO.	Gene type	*P. guangdongensis*	*P. zijinensis*
1	ATP synthase protein	*atp1*	*atp1*
2		*atp4*	*atp4*
3			*atp6*
4		*atp8*	*atp8*
5		*atp9*	*atp9*
6	heme exporter protein	*ccmB*	*ccmB*
7	cytochrome c biogenesis	*ccmC*	*ccmC*
8	cytochrome c maturation	*ccmFc*	*ccmFc*
9		*ccmFN*	*ccmFN*
10	cytochrome b	*cob*	*cob*
11	cytochrome c oxidase	*cox1*	*cox1*
12		*cox2*	*cox2*
13		*cox3*	*cox3*
14	cytochrome b	*cytB*	*cytB*
16	Maturase		*mat-R*
17	Trimethylamine methyltransferase	*mttB*	*mttB*
18	NADH dehydrogenase	*nad1*	*nad1*
		nad2	
19		*nad3*	*nad3*
20			*nad4*
21		*nad4L*	*nad4L*
22		*nad5*	*nad5*
23		*nad6*	*nad6*
24		*nad7*	*nad7*
25	ribosomal protein		*rpl10*
26		*rpl16*	*rpl16*
27		*rpl2*	*rpl2*
28		*rpl5*	*rpl5*
			rps1
29		*rps10*	*rps10*
30		*rps12*	*rps12*
31		*rps13*	*rps13*
32		*rps14*	*rps14*
33		*rps19*	*rps19*
			rps2
34		*rps3*	*rps3*
35		*rps4*	*rps4*
36		*rps7*	
38	Open reading frame	*orf103a*	*orf103a*

			orf104b
39		*orf109c*	*orf109c*
40		*orf112c*	
41		*orf116*	*orf116*
42		*orf121a*	*orf121a*
43		*orf125f*	*orf125f*
44		*orf134*	*orf134*
45		*orf142*	*orf142*
46		*orf171*	*orf171*
47		*orf214*	*orf214*
48			*orf216*
49		*orfX*	*orfX*
50	ribosomal RNA	*rrn5*	*rrn5*
51	transfer RNA	*trnC*^{GCA}	*trnC*^{GCA}
52		*trnD*^{GUC}	*trnD*^{GUC}
53		*trnE*^{TTC}	*trnE*^{TTC}
54		*trnF*^{GAA}	*trnF*^{GAA}
55		*trnfM*^{CAU}	*trnfM*^{CAU}
56		*trnH*^{GUG}	*trnH*^{GUG}
57		*trnH*^{GTG}	*trnH*^{GTG}
58		*trnI*^{CAU}	*trnI*^{CAU}
59		*trnI*^{AAT}	*trnI*^{AAT}
60		*trnK*^{UUU}	*trnK*^{UUU}
61		*trnL*	
		trnL^{TTT}	*trnL*^{TTT}
62		*trnM*^{CAU}	*trnM*^{CAU}
63		*trnM*^{CAT}	*trnM*^{CAT}
64		*trnN*^{GTT}	*trnN*^{GTT}
65		*trnQ*^{TTG}	*trnQ*^{TTG}
66			*trnS*^{GCU}
67			*trnS*^{TGA}
68		*trnW*^{CCA}	*trnW*^{CCA}
69		*trnY*^{AUA}	*trnY*^{AUA}
70		*trnY*^{GTA}	*trnY*^{GTA}

Supplementary Table 42. W value of other genes except for photosynthetic homologs of *P. guangdongensis* and *P. zijinensis* (see separate file).

Supplementary Table 43. W value of photosynthetic homologs of *P. guangdongensis* and *P. zijinensis* (see separate file).

Supplementary Table 44. W value of each branch after the divergence of *P. guangdongensis* and *P. zijinensis* using *A. shenzhenica* as outgroup (see separate file).

Supplementary Table 45. Gene element length for ten plant species (see separate file).

Supplementary Table 46. Gene IDs for leaf initiation and development (see separate file).

Supplementary References

1. Graham, S. W., Lam, V. K. Y. & Merckx, V. S. F. T. Plastomes on the edge: the evolutionary breakdown of mycoheterotroph plastid genomes. *New Phytol.* **214**, 48–55 (2017).
2. Barrett, C. F. & Davis, J. I. The plastid genome of the mycoheterotrophic *Corallorhiza striata* (Orchidaceae) is in the relatively early stages of degradation. *Am. J. Bot.* **99**, 1513–1523 (2012).
3. Estiati, A. & T., T. K. The ribosomal protein S7 gene is transcribed and edited in sugar beet mitochondria. *Physiol. Plant.* **102**, 325–327 (1998).
4. Zhuo, D. & Bonen, L. Characterization of the S7 ribosomal protein gene in wheat mitochondria. *Mol. Gen. Genet.* **236**, 395–401 (1993).
5. Cusimano, N. & Wicke, S. Massive intracellular gene transfer during plastid genome reduction in nongreen Orobanchaceae. *New Phytol.* **210**, 680–693 (2016).
6. Moon, J. & Hake, S. How a leaf gets its shape. *Curr. Opin. Plant Biol.* **14**, 24–30 (2011).
7. Byrne, M. E. Making leaves. *Curr. Opin. Plant Biol.* **15**, 24–30 (2012).
8. Bar, M. & Ori, N. Leaf development and morphogenesis. *Dev.* **141**, 4219–4230 (2014).
9. Ichihashi, Y. & Tsukaya, H. Behavior of leaf meristems and their modification. *Front. Plant Sci.* **6**, 1–8 (2015).
10. Prasad, K. *et al*. *Arabidopsis* PLETHORA transcription factors control phyllotaxis. *Curr. Biol.* **21**, 1123–1128 (2011).
11. Pinon, V., Prasad, K., Grigg, S. P., Sanchez-Perez, G. F. & Scheres, B. Local auxin biosynthesis regulation by PLETHORA transcription factors controls phyllotaxis in *Arabidopsis*. *Proc. Natl. Acad. Sci. U. S. A.* **110**, 1107–1112 (2013).
12. Křeček, P. *et al*. The PIN-FORMED (PIN) protein family of auxin transporters. *Genome Biol.* **10**, 1–11 (2009).
13. Yoshida, S., Mandel, T. & Kuhlemeier, C. Stem cell activation by light guides plant organogenesis. *Genes Dev.* **25**, 1439–1450 (2011).
14. Hamann, T., Mayer, U. & Jürgens, G. The auxin-insensitive *bodenlos* mutation affects

primary root formation and apical-basal patterning in the *Arabidopsis* embryo. *Development* **126**, 1387–1395 (1999).

15. Benfey, P. N. *et al*. Root development in *Arabidopsis*: four mutants with dramatically altered root morphogenesis. *Development* **70**, 57–70 (1993).
16. Scheres, B. *et al.* Mutations affecting the radial organisation of the *Arabidopsis* root display specific defects throughout the embryonic axis. *Development* **121**, 53–62 (1995).
17. Galway, M. E. *et al.* The *TTG* gene is required to specify epidermal cell fate and cell patterning in the *Arabidopsis* root. *Dev. Biol.* **166**, 740–754 (1994).
18. Walker, A. R. *et al*. The *TRANSPARENT TESTA GLABRA1* locus, which regulates trichome differentiation and anthocyanin biosynthesis in *Arabidopsis*, encodes a WD40 repeat protein. *Plant Cell* **11**, 1337–1349 (1999).
19. Bernhardt, C. *et al.* The bHLH genes *GLABRA3* (*GL3*) and *ENHANCER OF GLABRA3* (*EGL3*) specify epidermal cell fate in the *Arabidopsis* root. *Development* **130**, 6431–6439 (2003).
20. Lee, M. M. & Schiefelbein, J. WEREWOLF, a MYB-related protein in *Arabidopsis*, is a position-dependent regulator of epidermal cell patterning. *Cell* **99**, 473–483 (1999).
21. Wada, T., Tachibana, T., Shimura, Y. & Okada, K. Epidermal cell differentiation in *Arabidopsis* determined by a *Myb* homolog, *CPC*. *Science*. **277**, 1113–1116 (1997).
22. Schellmann, S. *et al. TRIPTYCHON* and *CAPRICE* mediate lateral inhibition during trichome and root hair patterning in *Arabidopsis*. *EMBO J.* **21**, 5036–5046 (2002).
23. Kirik, V., Simon, M., Huelskamp, M. & Schiefelbein, J. The *ENHANCER OF TRY AND CPC1* gene acts redundantly with *TRIPTYCHON* and *CAPRICE* in trichome and root hair cell patterning in *Arabidopsis*. *Dev. Biol.* **268**, 506–513 (2004).
24. Zhang, G. Q. *et al.* The *Dendrobium catenatum* Lindl. genome sequence provides insights into polysaccharide synthase, floral development and adaptive evolution. *Sci. Rep.* **6**, 19029 (2016).
25. Zhang, G. Q. *et al.* The *Apostasia* genome and the evolution of orchids. *Nature* **549**, 379–383 (2017).

26. Cai, J. *et al.* The genome sequence of the orchid *Phalaenopsis equestris*. *Nat. Genet.* **47**, 65–72 (2015).

27. Yuan, Y. *et al.* The *Gastrodia elata* genome provides insights into plant adaptation to heterotrophy. *Nat. Commun.* **9**, 1615 (2018).
