# Supplementary material for: Genomes of leafy and leafless Platanthera orchids illuminate the evolution of mycoheterotrophy
Source: Nat Plants. 2022 Apr 21;8(4):373–88. doi: 10.1038/s41477-022-01127-9 (PMC9023349; doi:10.1038/s41477-022-01127-9)
Supplement: Supplementary file 2 — Reporting Summary [file 41477_2022_1127_MOESM2_ESM.pdf]

nature portfolio

Corresponding author(s): Zhong-Jian Liu

Last updated by author(s): Mar 2, 2022

Reporting Summary

Nature Portfolio wishes to improve the reproducibility of the work that we publish. This form provides structure for consistency and transparency in reporting. For further information on Nature Portfolio policies, see our Editorial Policies and the Editorial Policy Checklist.

Statistics

For all statistical analyses, confirm that the following items are present in the figure legend, table legend, main text, or Methods section.

n/a	Confirmed	
☐	☒	The exact sample size (*n*) for each experimental group/condition, given as a discrete number and unit of measurement
☐	☒	A statement on whether measurements were taken from distinct samples or whether the same sample was measured repeatedly
☐	☒	The statistical test(s) used AND whether they are one- or two-sided<br>*Only common tests should be described solely by name; describe more complex techniques in the Methods section.*
☒	☐	A description of all covariates tested
☒	☐	A description of any assumptions or corrections, such as tests of normality and adjustment for multiple comparisons
☐	☒	A full description of the statistical parameters including central tendency (e.g. means) or other basic estimates (e.g. regression coefficient) AND variation (e.g. standard deviation) or associated estimates of uncertainty (e.g. confidence intervals)
☐	☒	For null hypothesis testing, the test statistic (e.g. *F*, *t*, *r*) with confidence intervals, effect sizes, degrees of freedom and *P* value noted<br>*Give P values as exact values whenever suitable.*
☐	☒	For Bayesian analysis, information on the choice of priors and Markov chain Monte Carlo settings
☒	☐	For hierarchical and complex designs, identification of the appropriate level for tests and full reporting of outcomes
☒	☐	Estimates of effect sizes (e.g. Cohen's *d*, Pearson's *r*), indicating how they were calculated

Our web collection on statistics for biologists contains articles on many of the points above.

Software and code

Policy information about availability of computer code

Data collection	Total RNA was extracted from using RNAprep Pure Plant Kit (Tiangen Biotech) following the manufacturer's instructions. Total RNA was qualified and quality-checked using a Nano Drop and Agilent 2100 bioanalyzer (Thermo Fisher Scientific, MA, USA). Libraries were constructed using the mRNA-Seq Prep Kit (Illumina) and then sequenced by the Illumina HiSeq 4000 platform.
Data analysis	Genome size of P. zijinensis and P. guangdongensis were esitimated by jellyfish v2.1.4 and genomeScope v2.0. We used CANU to correct the Pacbio subreads, and used flye v2.4.2-release to assemble the genome. LACHESIS was used to construct the scaffold clusters into 21 chromosome. The OrthoMCL groups table and the time tree were analyzed via the Cafe v3 software. Phylogenomic dating was conducted in PAML v4 MCMCTree. Identification of orthologues was performed first via reciprocal BLASTP with E values < 1×10–5.

For manuscripts utilizing custom algorithms or software that are central to the research but not yet described in published literature, software must be made available to editors and reviewers. We strongly encourage code deposition in a community repository (e.g. GitHub). See the Nature Portfolio guidelines for submitting code & software for further information.

Data

Policy information about availability of data

All manuscripts must include a data availability statement. This statement should provide the following information, where applicable:

- Accession codes, unique identifiers, or web links for publicly available datasets
- A description of any restrictions on data availability
- For clinical datasets or third party data, please ensure that the statement adheres to our policy

Genome sequences and whole-genome assemblies have been submitted to the National Center for Biotechnology Information (NCBI) database under BioProject PRJNA739531.

Field-specific reporting

Please select the one below that is the best fit for your research. If you are not sure, read the appropriate sections before making your selection.

☒ Life sciences ☐ Behavioural & social sciences ☐ Ecological, evolutionary & environmental sciences

For a reference copy of the document with all sections, see nature.com/documents/nr-reporting-summary-flat.pdf

Life sciences study design

All studies must disclose on these points even when the disclosure is negative.

Sample size	The sequenced individual P. zijinensis and P. guangdongensis were collected from natural wild individuals without any specific selection. The detail information was written in "method" in the main text.
Data exclusions	No data exclusions in this manuscript.
Replication	There biological replications were performed for RNA-seq and methylome sequencing.
Randomization	No randomization in this manuscript as samples were not allocated into experimental groups.
Blinding	No blinding in this manuscript as the data were not allocated into groups

Reporting for specific materials, systems and methods

We require information from authors about some types of materials, experimental systems and methods used in many studies. Here, indicate whether each material, system or method listed is relevant to your study. If you are not sure if a list item applies to your research, read the appropriate section before selecting a response.

Materials & experimental systems

n/a	Involved in the study	
☒	☐	Antibodies
☒	☐	Eukaryotic cell lines
☒	☐	Palaeontology and archaeology
☒	☐	Animals and other organisms
☒	☐	Human research participants
☒	☐	Clinical data
☒	☐	Dual use research of concern

Methods

n/a	Involved in the study	
☒	☐	ChIP-seq
☒	☐	Flow cytometry
☒	☐	MRI-based neuroimaging
